# Direct Pd-Catalyzed β‑C(sp^3^)–H Hydroxylation of Aliphatic Carboxylic Acids

**DOI:** 10.1021/acs.orglett.5c00614

**Published:** 2025-06-11

**Authors:** Sourjya Mal, Tianxiao Xu, Manuel van Gemmeren

**Affiliations:** † Otto Diels-Institut für Organische Chemie, Christian-Albrechts Universität zu Kiel, Otto-Hahn-Platz 4, 24118 Kiel, Germany

## Abstract

β-Hydroxy carbonyl scaffolds are of paramount importance due to their presence in a variety of natural products and bioactive molecules. In this study, we present a direct palladium-catalyzed β-C­(sp^3^)–H hydroxylation of aliphatic carboxylic acids. The reported method employs convenient and readily available TBHP as an oxidant and provides an efficient, one-step route to a diverse range of β-hydroxy acids. Key aspects include the compatibility of this method with challenging α-non-quaternary carboxylic acids as substrates and excellent functional group tolerance. This newly developed method is expected to prove useful for generating compound libraries relevant to multiple disciplines.

## Graphical abstract

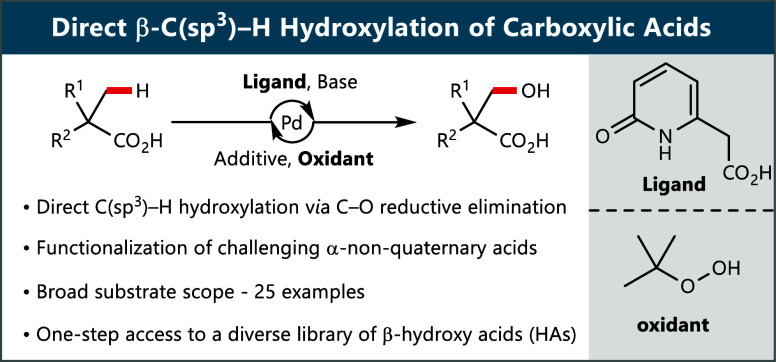

Hydroxyl groups are the most prevalent functional group in nature^1,2^ and feature a highly versatile reactivity. Their widespread occurrence in agrochemicals, natural products,^3^ and bioactive compounds^4^ illustrates the key influence the incorporation of hydroxyl group exerts on the biological and chemical properties of molecular frameworks. While functional group interconversion approaches^5^ enable the introduction of hydroxyl group into organic molecules, they remain limited by the reliance on suitable prefunctionalized starting materials. Consequently, the direct oxidation of chemically inert C­(sp^3^)–H bonds represents an attractive, single-step alternative. In this regard, transition-metal-catalyzed C­(sp^3^)–H functionalization reactions have emerged as a promising approach. The homolytic cleavage of C­(sp^3^)–H bonds via a hydrogen atom transfer (HAT) event, mimics the functional properties of cytochrome P450 to a certain extent and thus typically enables the C–H hydroxylation of weaker tertiary (3°) and secondary (2°) C­(sp^3^)–H bonds, as the reactivity and selectivity is governed by the bond dissociation energies (BDEs) of the respective C–H bonds.^6^ Complementary substrate scopes and regioselectivities (preference for 1 °C­(sp^3^)–H over 2° or 3 °C­(sp^3^)–H) are expected for reactions proceeding through a C–H activation followed by oxidation. This reactivity profile has been studied extensively for the hydroxylation of aromatic C­(sp^2^)–H bonds using various directing groups (DGs).^7^ However, the use of this strategy for the hydroxylation of C­(sp^3^)–H bonds has mostly remained limited to the use of amines as substrates.^8^ β-Hydroxy acids are widespread structural motifs in natural products^9^ and bioactive compounds,^10^ also serving as versatile building blocks in organic synthesis (Scheme 1A). Notably, hydroxy fatty acids (HFAs) represent an important class of lipids that have found tremendous applications in multiple disciplines, ranging from lubricants,^11^ surfactants,^12^ and cosmetics^13^ to synthetic precursors in polymer chemistry.^14^ Additionally, HFAs are used as precursors for a range of signaling molecules.^10b,15^ The importance of these scaffolds renders a synthesis through a directed C­(sp^3^)–H bond activation/hydroxylation highly attractive complementing for example the direct aldol reaction of carboxylic acids, which primarily yields β-(2°)­hydroxy acids.^16^ Related state-of-the-art methods to access hydroxy acids rely on indirect routes (Scheme 1B), such as the conversion of carboxylic acid moiety into a stronger directing group (DG), followed by C­(sp^3^)–H hydroxylation and the removal of the DG.^17^ Alternatively, the direct palladium-catalyzed-C­(sp^3^)–H activation of free carboxylic acids has been used to generate intermediates such as β-lactones^18^ or β-acetoxylated proucts,^19^ which are then subjected to hydrolysis. Although these protocols give access to the target compounds, a direct β-C­(sp^3^)–H hydroxylation of a broad range of carboxylic acids, especially the notoriously challenging^20^ α-non-quaternary carboxylic acids which typically exhibit poor reactivity in analogous transformations,^18,19a^ would be more convenient and efficient in terms of step- and atom-economy, but has remained elusive until date. Building upon our prior experience in the C­(sp^3^)–H activation of carboxylic acids,^21^ we herein report the development of a protocol for the direct synthesis of a wide range of hydroxy acids from their respective acids in a single step.

Prompted by our recent study where the suitable combination of oxidant and ligand facilitated challenging C–F reductive elimination via the formation of high-valent Pd­(IV),^22^

**Scheme 1:**
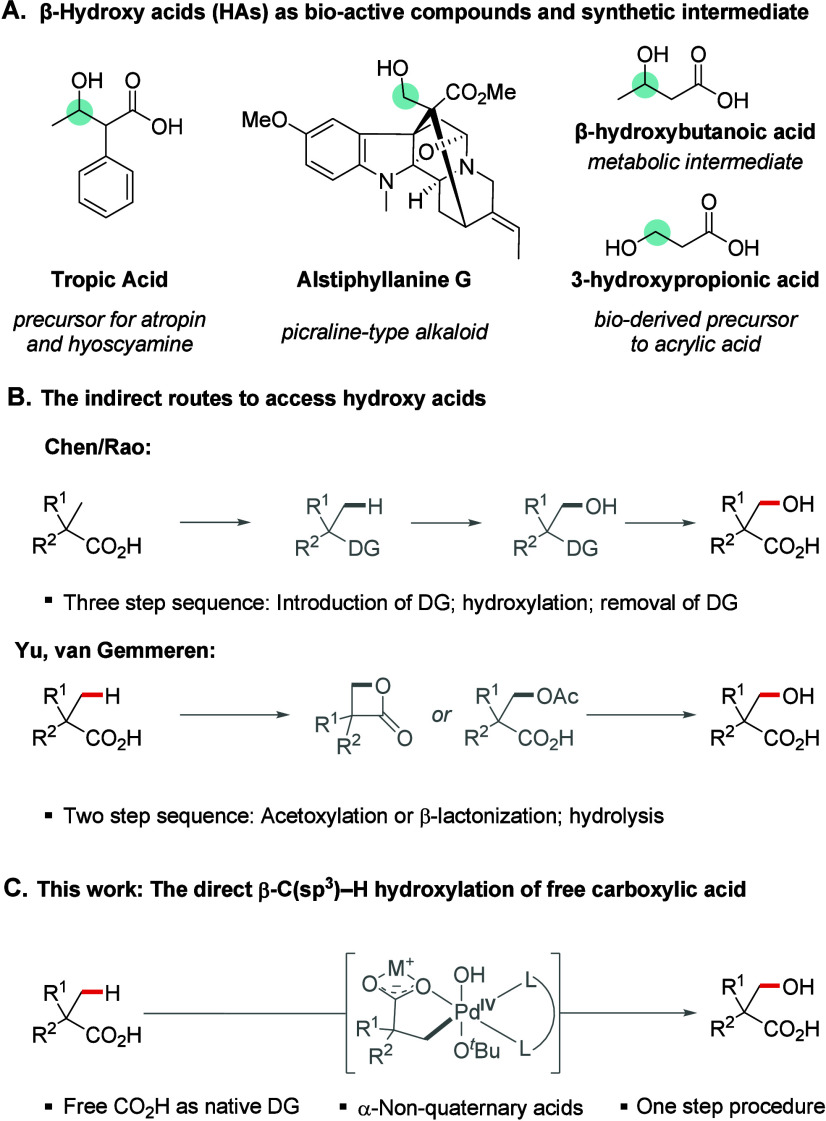
(A) Importance of β-Hydroxy Acids as Bio-active Compounds and Synthetic Intermediates, (B) Literature Precedent, and (C) Aim of This Work

we envisioned that a similar approach could be used to induce C–O reductive elimination (RE) and afford the desired product. Based on this reasoning, we initiated our optimization studies with 2-methyl valeric acid as a model substrate. Initial screening of various ligands and oxidants identified TBHP and a pyridone-derived ligand **L1** as the most promising combination (see Supporting Information for further details).^7b,7c^ After identifying the best reaction conditions, we re-evaluated some of the most important findings as deviations from the optimized reaction conditions (Table 1). Ligand **L1** delivered 48% yield (Table 1), while in the absence of either this ligand, base, or TBHP no product formation was observed, highlighting the crucial role of these components for the desired reactivity. The omission of DMF substantially reduced the yield, suggesting that its beneficial effect is likely due to its participation as a labile ligand,^23^ which has been described in literature to stabilize otherwise coordinatively unsaturated high-valent Pd­(IV) intermediates and/or facilitate ligand exchange processes, particularly in the catalyst regeneration step. Replacing TBHP with *^t^* BuOO*^t^* Bu yielded no product, suggesting that an oxidant with an −OH group is essential. Switching to *m*-CPBA or sodium percarbonate did not improve the yield, indicating the beneficial effect exerted by the oxidant bearing -*^t^* BuO fragment. Next, we re-evaluated the choice of ligand by probing variations to ligand motifs that gave promising results during our optimization studies. The introduction of methyl group(s) on the side chain (**L2**, 39% and **L3**, 35%) proved to be detrimental. Ligands leading to five-membered ring chelation of the Pd-catalyst resulted in poor yields (**L4**, 12% and **L5**, 30%). Similarly, six-membered chelating ligands with a β-alanine-derived backbone continued to provide moderate yields (**L6**, 20% and **L7**, 35%). Despite the extensive structural fine-tuning including the CMD-promoting amide groups (see SI for more details), no further improvement of the yield was observed.

**Table 1:**
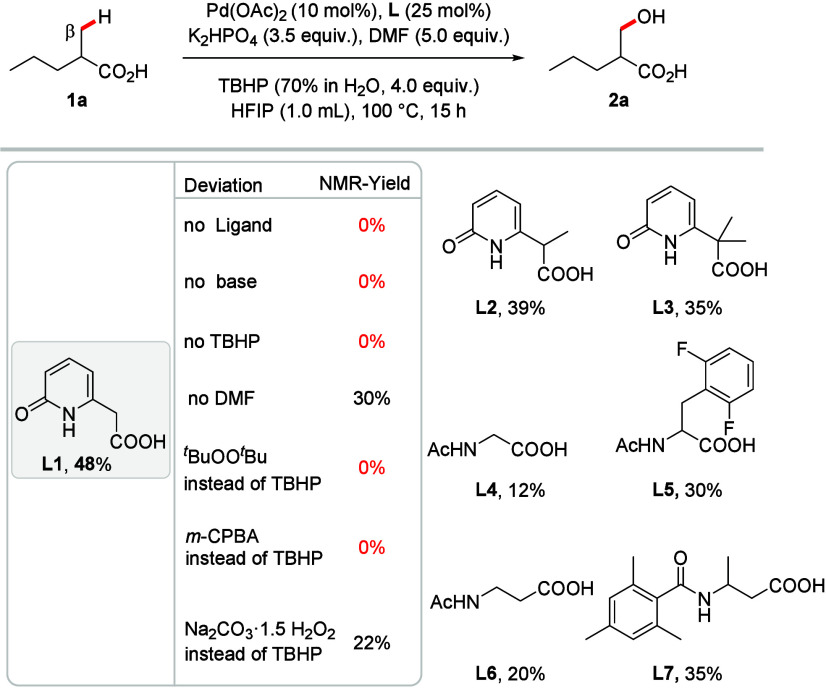
Optimization of the Reaction Conditions^a^

a Reactions were conducted on a 0.1 mmol scale. Yields were determined by ^1^H NMR analysis of the crude reaction mixture using CH_2_Br_2_ as an internal standard. Mass balance predominantly accounted for by unconverted starting material.

Considering the strong coordinating ability of the hydroxylated or acetoxylated product to Pd,^19b,24^ we examined whether product poisoning might be the reason for incomplete conversions. Interestingly, when the hydroxylation of **1a** was conducted, in the presence of **2t**, the yield and conversion were markedly reduced (see SI for more details). This implies that the reaction is slowed due to a competing coordination of the product to the catalyst and therefore unavoidably halts before reaching full conversion. Having reached moderate yet synthetically useful yields, we considered that the ease, as compared to multistep approaches and syntheses based on functional group interconversions, with which our method can generate compound libraries from easily accessible and structurally diverse starting materials will render our method useful for the scientific community.

Having identified suitable reaction conditions, we investigated the substrate scope of our method (Scheme 2). Commercially available linear and branched aliphatic acids were first tested under our reaction conditions and provided satisfactory yields of the respective hydroxy acids **2b** (44%), **2c** (42%), **2d** (44%), and **2e** (42%). Substrates bearing cyclic substituents on the side chain, including three- (**2f**, 48%), four- (**2g**, 45%), and five- (**2h**, 52%) membered rings were well tolerated. A range of synthetically useful functional groups on the side chain, such as fluoro (**2i**, 44%), chloro (**2j**, 43%), trifluoromethyl (**2k**, 41%), and protected hydroxyl groups (**2l**, 51% and **2m**, 48%), were found to be compatible with our reaction conditions. Product **2n** (45%) with a phenyl group in the side chain was obtained in useful yield. We furthermore evaluated a series of aryl-containing substrates featuring reactive benzylic C–H bonds in the proximity of the carboxylic acid moiety. Interestingly, a wide range of substituents including electron-rich arenes such as in **2o** (40%), and **2p** (30%) as well as electron-poor arenes such as in **2q** (36%), **2r** (42%), and **2s** (39%) were well tolerated, albeit giving the desired products in moderate yields. These results highlight the utility of our protocol for the hydroxylation of a wide range of α-non-quaternary carboxylic acids. We furthermore explored the applicability of our protocol to the hydroxylation of α-quaternary carboxylic acids.

**Scheme 2:**
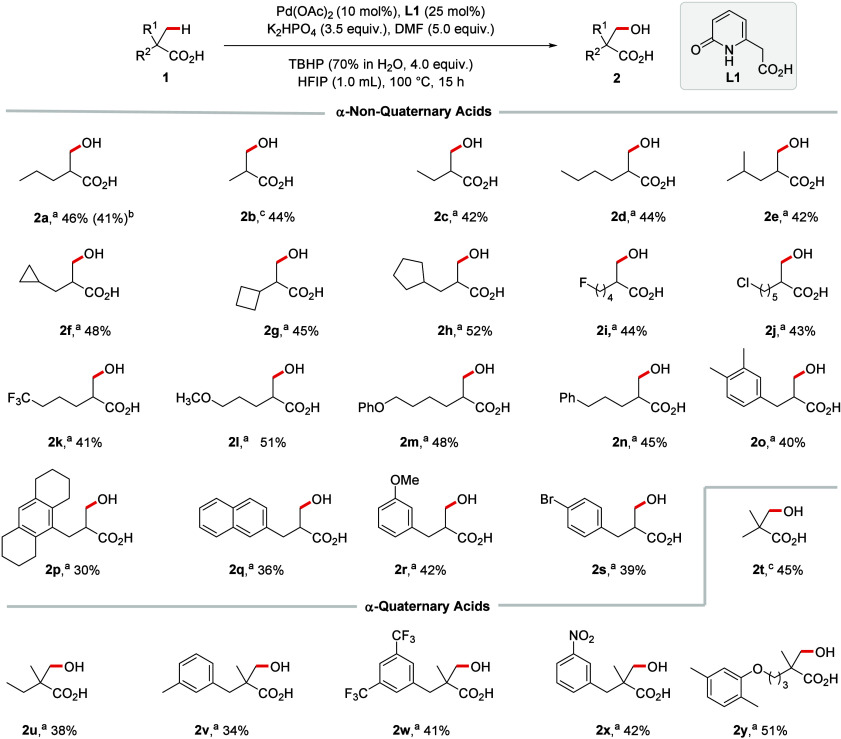
Substrate Scope of Direct β-C­(sp^3^)—H Hydroxylation of Aliphatic Acids^d^

a Isolated after conversion to the respective benzyl ester.
b The reaction was performed on a 1.0 mmol scale.
c Isolated after conversion to the respective naphthalen-2-ylmethyl ester. Mass balance predominantly accounted for by unconverted starting material.
d Reactions were conducted on a 0.2 mmol scale.

Commercially available pivalic acid and 2,2-dimethyl butyric acid delivered the respective products **2t** (45%) and **2u** (38%) in useful yields. Aryl-containing substrates bearing methyl (**2v**, 34%), trifluoromethyl (**2w**, 41%), and nitro (**2x**, 42%) substituents were found to be compatible. Finally, our protocol was applied to gemfibrozil, an oral lipid-lowering drug, and afforded **2y** in 51% yield. Notably, exclusive monoselectivity was observed for α-quaternary carboxylic acids (**2t**-**2u**). This is presumably due to the fact that when the already hydroxylated products coordinate to Pd, they act as chelating ligands via the carboxylate and hydroxy groups, which places the remaining methyl groups out of reach for the catalyst preventing a second C–H activation on the same substrate and thereby suppressing an iterative hydroxylation.^19b^ Presumably for the same reason, chelating ligands (**L2** and **L3**) bearing methyl group(s) at the α-position did not lead to the formation of their respective β-hydroxylated products.

In accordance with literature precedents,^25^ we propose that our transformation proceeds via a Pd­(II)/Pd­(IV) catalytic cycle, as shown in Scheme 3. First, the coordination of external ligand (**L1**) to Pd­(OAc)_2_ generates the active catalyst. The following C–H activation step is accelerated by both the countercation (K^+^) and the ligand and occurs via a concerted metalation–deprotonation (CMD) mechanism,^25a^ where the ligand functions as an internal base to produce **Int-1**. Considering the well-documented ability of peroxides to oxidize Pd­(II) to Pd­(IV),^25b–25d^ **Int-1** is expected to undergo a rate-limiting oxidative addition with TBHP, leading to the formation of **Int-2**.^25e^ From this stage, two plausible pathways could lead to the desired product: a direct C–O reductive elimination (RE) induced by *^t^* BuO^8a^ or an S_N_2-like reaction in which H_2_O/HO^–^ attacks the carbon center bound to the high-valent Pd­(IV) species,^25h^ both of these processes would result in the immediate formation of desired product (*path A*). To probe whether a direct RE or an S_N_2-like reaction are operative, **1a** was subjected to the otherwise optimized reaction conditions but using anhydrous TBHP (in decane) instead of aqueous TBHP, leading to a virtually identical reaction outcome as judged by the ^1^H NMR-yield. Furthermore, the addition of a nucleophile such as MeOH did not result in the formation of analogous β-methoxy acid (see SI for more details). This strongly speaks against the involvement of an S_N_2-like process occurring at the high-valent Pd­(IV) species,^25h^ which should be influenced by the presence/absence of external water or other nucleophilic species. This implies that the OH-ligands formed in the coordination sphere of Pd from TBHP during the oxidative addition are subsequently introduced into the product via RE and act as the primary source of the −OH group in this reaction.

**Scheme 3:**
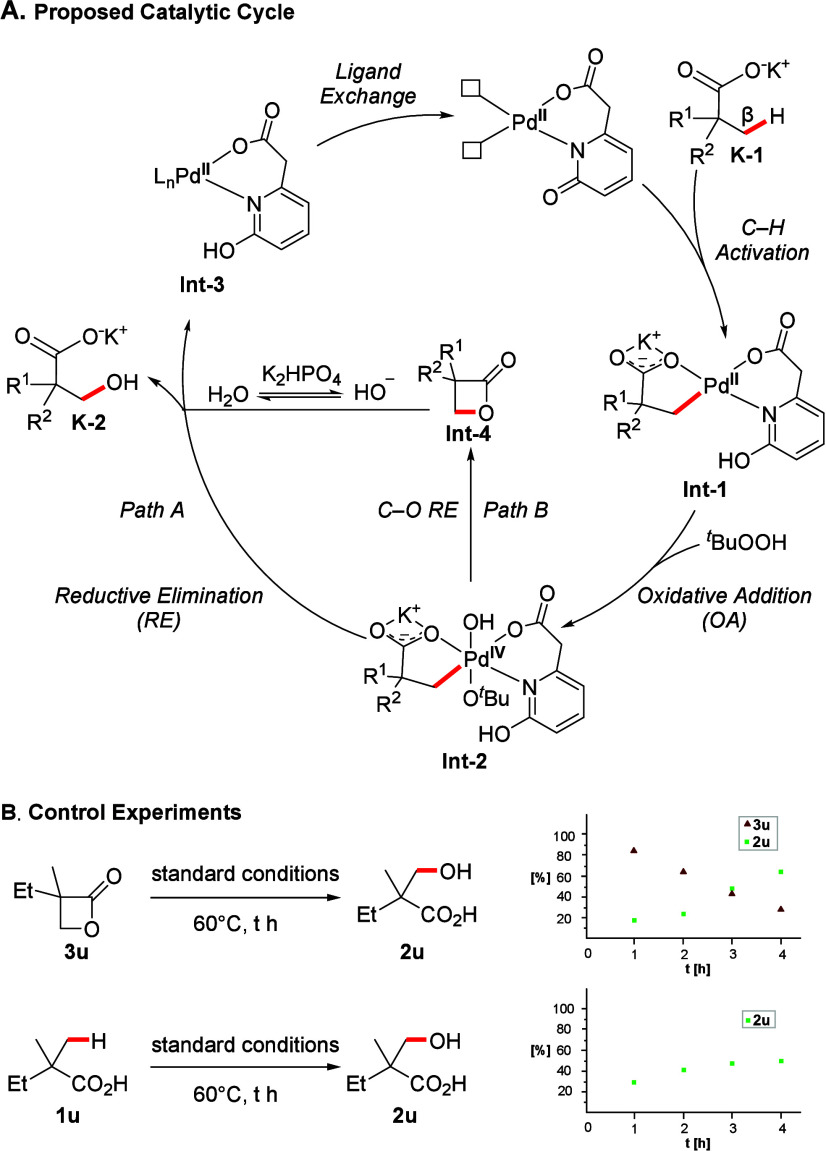
Proposed Catalytic Cycle and Mechanistic Experiments^a^

a Reactions Conducted on a 0.1 mmol Scale and Yields and Conversions Determined by ^1^H NMR Spectroscopic Analysis of the Crude Reaction Mixture Using CH_2_Br_2_ as an Internal Standard

A further alternative that needed to be considered was that **Int-2** could undergo a C–O RE to form a β-lactone **Int-4**,^18,25e^ which would then undergo a subsequent S_N_2 reaction with H_2_O/HO^–^ to afford **2**. We thus performed several control experiments to elucidate which one of these pathways is operative. When β-lactone **3u** was exposed to the standard conditions, **2u** (73%) was obtained (see SI for more details), with nearly complete consumption of the starting material. The observed outcome is expected as both base (K_2_HPO_4_) and H_2_O are known to facilitate such ring opening or hydrolysis processes at elevated temperatures.^26^ However, the decomposition of the β-lactone to the final product does not implicate its involvement as reaction intermediate. We thus proceeded to identify milder reaction conditions under which the β-lactone would remain at least partially stable. The decomposition of **3u** at 60 °C revealed that more than 80% of **3u** could still be detected after 1 h and even after 4 h 26% of the starting material could still be detected, while the remaining material had mostly been converted to product **2u** (62%, Scheme 3B). When we applied these modified reaction condition substrate **1u**, we observed 30% of **2u** after 1 h and 46% of **2u** after 4h while only minute traces of β-lactone **3u** were detected (<2%). This clearly demonstrates that **2u** cannot have formed via **3u** as an intermediate, since the relative rates of product formation and decomposition of **3u** would cause a substantial accumulation of the β-lactone were it to be a reaction intermediate, thus allowing us to rule out *path B* and providing support for a direct formation of the hydroxy acid products through RE (*path A*).

In summary, we have developed a protocol that enables a direct β-C­(sp^3^)–H hydroxylation of aliphatic carboxylic acids. The use of convenient and inexpensive TBHP, along with suitable choice of reagents were crucial in establishing this protocol. The reported synthetic method was shown to be effective for challenging α-non-quaternary carboxylic acids and displays considerable functional group tolerance, enabling the efficient access to a wide range of β-hydroxy acids in a single step. Given the broad applicability of β-hydroxy acids across various fields, we anticipate that the new compound libraries that become accessible through our method will be relevant for various fields.

## Supplementary Material



## Data Availability

All underlying data supporting this work are available in the article and its Supporting Information.

## Notes

Experimental procedures and characterization of the compounds obtained (PDF)
