# Supplementary material for: Direct Pd-Catalyzed β‑C(sp3)–H Hydroxylation of Aliphatic Carboxylic Acids
Source: Org Lett. 2025 Jun 11;27(25):6551–6. doi: 10.1021/acs.orglett.5c00614 (PMC12210258; doi:10.1021/acs.orglett.5c00614)
Supplement: Supplementary file 1 [file ol5c00614_si_001.pdf]

The Direct Pd-Catalyzed β-C(sp^3)–H Hydroxylation of Aliphatic Carboxylic Acids

Sourjya Mal, [‡,a] Tianxiao Xu, [‡,a] and Manuel van Gemmeren*[,a]

[a]Otto Diels-Institut für Organische Chemie, Christian-Albrechts Universität zu Kiel, Otto-Hahn-Platz 4, 24118 Kiel (Germany).

[‡] These authors contributed equally to this work

Content

1. General Information 2

2. Preparation of Ligands 4

3. Optimization of the Hydroxylation Reaction 8

4. Synthesis of Starting Materials 16

5. Scope Studies of the Hydroxylation Reaction 17

6. Preliminary Mechanistic Investigations 33

7. References 38

8. NMR Spectra 43

1. General Information

Solvents, Reagents and Techniques

All reactions were conducted in oven-dried glassware (100 °C). Reaction temperatures refer to the temperature of the aluminum-block or oil bath surrounding the reaction vessel. Commercially available chemicals were obtained from ABCR, Acros Organics, BLD-pharm, Alfa Aesar, Deutero, Eurisotop, Fluorochem, Sigma Aldrich, or TCI Europe and used as received. HFIP was purchased from Fluorochem and used as received. Solvents used for column chromatography were distilled prior to use.

Chromatography

Analytical thin layer chromatography (TLC) was performed on silica gel ALUGRAM Xtra SIL G/UV254 plates (Macherey-Nagel) or aluminum oxide 150 F254, neutral plates (Merck). Compounds were visualized by ultraviolet light (254 nm or 366 nm) or by staining with $KMnO_4$ (1 g $KMnO_4$, 6 g K_2CO_3 and 0.1 g KOH in 100 mL of H_2O) or bromocresol green (40 mg bromocresol green in 100 mL EtOH; addition of $0.1M_{(aq.)}$ NaOH until the blue color appears in the solution) and developed with a heat gun if necessary. Flash chromatography was performed on silica gel 60M (0.04-0.063 mm) or aluminum oxide (aluminum oxide 90, neutral, activity level 1) with a positive nitrogen overpressure.

Nuclear Magnetic Resonance (NMR) Spectroscopy

^{1}H, ^{13}C, and ^{19}F NMR spectra were recorded at 25 °C on a Bruker AvanceNeo 500 or a Bruker Avance 600 device. ^{13}C NMR were recorded using broad band proton decoupling unless reported otherwise. Chemical shifts (δ) are given relative to tetramethylsilane (TMS) and using the residual solvent peaks for calibration. ^{19}F-NMR spectra are externally referenced with CCl_3F.Chemical shifts are reported with two decimal numbers (^{1}H) or one decimal number (^{13}C, ^{19}F), a second decimal number is provided if needed to distinguish two clearly separated signals that would otherwise be rounded to the same chemical shift. Data is reported in the following order: Chemical shift (multiplicity [s = singlet, d = doublet, t = triplet, q = quartet, quint = quintet, hept = septet, m = multiplet, br = broad signal], coupling constant (*J*, Hz) and integration). All NMR-spectra were processed using MestReNova.

General remarks for the scope entries: Some scope entries were found to contain traces of cyclohexane (δ = 1.43 ppm, originating from a contamination of the pentane used during purification), which could not be removed completely due to the volatility of the respective

products upon extended exposure to reduced pressure. In all of these cases we verified that, the purity of the compounds described remains above 95%.

Infrared spectroscopy (IR)

IR-spectroscopy was performed on a Perkin Elmer ATR spectrometer. Samples were measured neat. The wave numbers (ν) of recorded IR-signals are reported in cm^{-1}.

Mass Spectrometry (MS)

High resolution mass spectra (HRMS) were recorded on a Jeol AccuTOF (EI) or a ThermoFisher Orbitrap (ESI) device.

2. Preparation of Ligands

L8-L11 were synthesized using the previously reported protocol from our laboratory.[1, 2]

2-(6-hydroxypyridin-2-yl)acetic acid (L1)

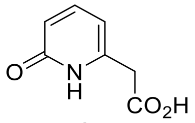

L1

Similar to a procedure by Yu and coworkers,[3] 2,6- difluoropyridine (1.15 g, 10.0 mmol) and acetonitrile (1.23 g, 30.0 mmol) were used. The target compound **L1** was obtained as a colorless solid (0.9 g, 5.9 mmol, 59%).

^{1}H-NMR (500 MHz, DMSO-d6): δ = 12.04 (br, 2H), 7.35 (dd, *J* = 9.2, 6.7 Hz, 1H), 6.20 (dd, *J* = 9.2, 1.1 Hz, 1H), 6.07 (dd, *J* = 6.8, 1.0 Hz, 1H), 3.50 (s, 2H). ppm

^{13}C-NMR (126 MHz, DMSO-d6): δ = 170.5, 162.9, 142.9, 140.8, 117.6, 105.6, 38.0 ppm.

HRMS (ESIpos) m/z: $[M +H]^+$ Calcd for $C_7H_8NO_3$ 154.0499, Found 154.0496.

IR (cm^{-1}): 3707, 2981, 1707, 1618, 1541, 1275, 1260, 1054, 1032, 1012, 764.

2-(6-hydroxypyridin-2-yl)propanoic acid

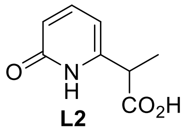

L2

Similar to a procedure by Yu and coworkers,[3] 2,6- difluoropyridine (862 mg, 7.50 mmol) and propanenitrile (275 mg, 5.00 mmol) were used. The target compound **L2** was obtained as a gray solid (292 mg, 1.75 mmol, 35%).

^{1}H NMR (600 MHz, MeOD): δ = 7.55 (dd, *J* = 9.1, 7.0 Hz, 1H), 6.43 (dd, *J* = 9.1, 1.0 Hz, 1H), 6.34 – 6.32 (m, 1H), 3.71 (q, *J* = 7.3 Hz, 1H), 1.51 (d, *J* = 7.3 Hz, 3H) ppm.

^{13}C NMR (151 MHz, MeOD): δ = 174.9, 166.2, 149.5, 143.7, 118.7, 106.6, 44.2, 16.9 ppm.

HRMS (EI) m/z: $[M]^+$ Calcd for $C_8H_9NO_3$ 167.0582, Found 167.0581.

IR (cm^{-1}): 2950, 1701, 1596, 1540, 1261, 1046, 1033, 998, 834.

2-(2-nitrobenzamido)acetic acid (L8)

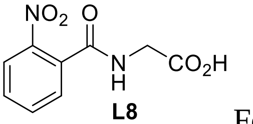

L8

Following the general procedure, 2-nitrobenzoyl chloride (1.3 g, 7.0 mmol) and methyl glycinate hydrochloride (1.1 g, 8.4 mmol) the target compound **L8** was obtained as a colorless solid (1.1 g, 4.9 mmol, 70%).

^{1}H NMR (500 MHz, $(CD_3)_2CO$): δ = 8.10 (s, 1H), 8.04 – 8.01 (m, 1H), 7.83 – 7.78 (m, 1H), 7.73 – 7.69 (m, 2H), 4.16 (d, J = 5.9 Hz, 2H) ppm.

^{13}C NMR (126 MHz, $(CD_3)_2CO$): δ = 171.0, 166.9, 148.5, 134.2, 133.4, 131.7, 129.9, 125.0, 41.7 ppm.

HRMS (ESIpos) m/z: $[M +H]^+$ Calcd for $C_9H_9N_2O_5$ 225.0506, Found 225.0504.

IR (cm^{-1}): 3680, 3279, 2981, 1721, 1648, 1523, 1260, 1054, 1033, 1012, 764.

2-(2-(trifluoromethyl)benzamido)acetic acid (L9)

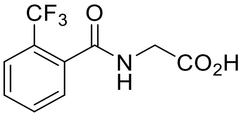

L9

Following the general procedure, 2-(trifluoromethyl)benzoyl chloride **(**1.5 g, 7.0 mmol) and methyl glycinate hydrochloride (1.1 g, 8.4 mmol) the target compound **L9** was obtained as a colorless solid (1.5 g, 6.1 mmol, 87%).

^{1}H NMR (500 MHz, $(CD_3)_2CO$): δ = 7.86 (s, 1H), 7.79 – 7.76 (m, 1H), 7.74 – 7.71 (m, 1H), 7.68 – 7.62 (m, 2H), 4.15 (d, J = 5.9 Hz, 2H) ppm.

^{13}C NMR (126 MHz, $(CD_3)_2CO$): δ = 171.1, 168.4, 137.2, 133.1, 130.7, 129.6, 127.8, 127.1 (q, J = 5 Hz), 124.8 (q, J = 273.0 Hz), 41.7 ppm.

HRMS (ESIneg) m/z: $[M-H]^-$ calcd for $C_{10}H_7O_3NF_3$ 246.0383, Found 246.0383.

IR (cm^{-1}): 3267, 1748, 1625, 1560, 1412, 1314, 1182, 1112, 1070, 1032, 874, 766.

3-(2,6-difluorophenyl)-2-(2-(trifluoromethyl)benzamido)propanoic acid (L10)

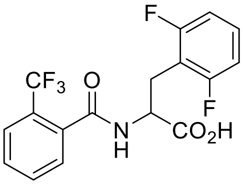

L10 Following the general procedure, 2-(trifluoromethyl)benzoyl chloride (650 mg, 3.10 mmol) and methyl 2-Amino-3-(2,6-difluorophenyl) propanoate (470 mg, 3.70 mmol) the target compound **L10** was obtained as a colorless solid (750 mg, 2.00 mmol, 64%). A mixture of pentane and EtOAc (50:50 - 60:40 v/v) acidified with HCOOH (0.2 mL for 100 mL of solvent mixture) was used as eluent for silica gel column chromatography.

^{1}H NMR (500 MHz, $(CD_3)_2CO$): δ = 7.89 (d, *J* = 8.3 Hz, 1H), 7.75 – 7.71 (m, 1H), 7.70 – 7.65 (m, 1H), 7.65 – 7.60 (m, 1H), 7.50 – 7.46 (m, 1H), 7.39 – 7.31 (m, 1H), 7.04 – 6.95 (m, 1H), 5.06 – 5.01 (m, 1H), 3.39 (dd, *J* = 13.9, 5.9 Hz, 1H), 3.24 (dd, *J* = 13.9, 9.2 Hz, 1H) ppm

^{13}C NMR (126 MHz, $CDCl_3$): δ = 172.3, 167.9, 163.8 (d, *J* = 8.4 Hz), 161.8 (d, *J* = 8.4 Hz), 137.0, 132.9, 130.7, 130.0 (q, *J* = 5 Hz), 129.4, 127.8, 127.1, 124.6 (q, *J* = 273.0 Hz) 112.1-111.9 (m), 52.5, 25.7 ppm.

HRMS (ESIpos) m/z: $[M+H]^+$ Calcd for $C_{17}H_{13}F_5NO_3$ 374.0810, Found 374.0802.

IR (cm^{-1}): 3268, 1710, 1654, 1544, 1471, 1315, 1265, 1176, 1035, 978, 784.

2-(2-nitrobenzamido)-3-phenylpropanoic acid (L11)

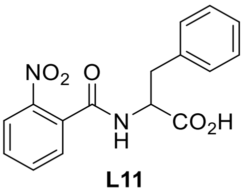

L11 Following the general procedure, 2-nitrobenzoyl chloride (1.3 g, 7.0 mmol) and methyl L-phenylalaninate hydrochloride (1.8 g, 8.4 mmol) the target compound **L11** was obtained as a colorless solid (1.3 g, 4.3 mmol, 61%).

^{1}H NMR (500 MHz, $(CD_3)_2CO$): δ = 8.00 (m, 1H), 7.77 – 7.65 (m, 2H), 7.52 (d, *J* = 7.5 Hz, 1H), 7.36 (m, 2H), 7.33 – 7.28 (m, 2H), 7.24 (m, 1H), 4.95 (m, 1H), 3.32 (dd, *J* = 14.1, 5.4 Hz, 2H), 3.18 (dd, *J* = 14.1, 8.2 Hz, 2H) ppm.

^{13}C NMR (126 MHz, $(CD_3)_2CO$): δ = 172.6, 166.3, 148.5, 138.1, 134.1, 133.4, 131.5, 130.2, 129.9, 129.2, 127.5, 124.9, 54.6, 38.1 ppm.

HRMS (ESIpos) m/z: $[M +H]^+$ Calcd for $C_{16}H_{15}N_2O_5$ 315.0975, Found 315.0970.

IR (cm^{-1}): 3381, 3005, 1746, 1624, 1527, 1402, 1349, 1266, 1184, 856, 756.

2-(6-hydroxypyridin-2-yl)-N-(methylsulfonyl)acetamide (L12)

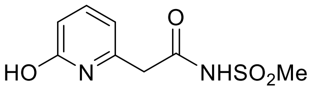

L12

Similar to a procedure by our group[2]. 2-(6-methoxypyridin-2-yl)acetic acid(0.84g, 5.0 mmol) and methanesulfonamide(0.52g, 5.5 mmol) were used, and then hydrolysed by using known procedure. The target compound **L12** was obtained as a colorless solid (0.89 g, 3.9 mmol, 78%).

^{1}H-NMR (500 MHz, DMSO-d6): δ = 11.93 (s, 1H), 11.55 (s, 1H), 7.36 (dd, *J* = 9.1, 6.7 Hz, 1H), 6.22 (d, *J* = 9.1 Hz, 1H), 6.07 (s, 1H), 3.56 (s, 2H), 3.24 (s, 3H). ppm

^{13}C-NMR (126 MHz, DMSO-d6): δ = 168.8, 163.3, 141.2, 118.7, 106.2, 41.5, 6.8, 0.6 ppm.

HRMS (ESIpos) m/z: $[M+H]^+$ Calcd for $C_8H_{11}N_2O_4S$ 231.0434, Found 231.0429.

IR (cm^{-1}): 3136, 1689, 1671, 1634, 1465, 1356, 1331, 1135, 973, 870, 778.

3. Optimization of the Hydroxylation Reaction

General Procedure for the Optimization Reactions:

An oven dried 10 mL Schlenk tube was charged with $Pd(OAc)_2$, ligand, base, oxidant, carboxylic acid (0.1 mmol) and HFIP. The vessel was transferred to a preheated aluminum block and the reaction mixture was stirred while heating at the indicated temperature. After the indicated time, the reaction mixture was allowed to cool to room temperature. Formic acid (0.1 mL) was added to the reaction mixture and it was filtered over a pad of Celite®. The residue was washed with CH_2Cl_2 (25-30 mL). All volatiles were removed under reduced pressure. Dibromomethane (17.4 mg, 0.100 mmol) and $CDCl_3$ (0.8 mL) were used to prepare a sample for NMR analysis. All yields during the optimization studies were determined by ^{1}H-NMR analysis of the crude reaction mixture using CH_2Br_2 as internal standard.

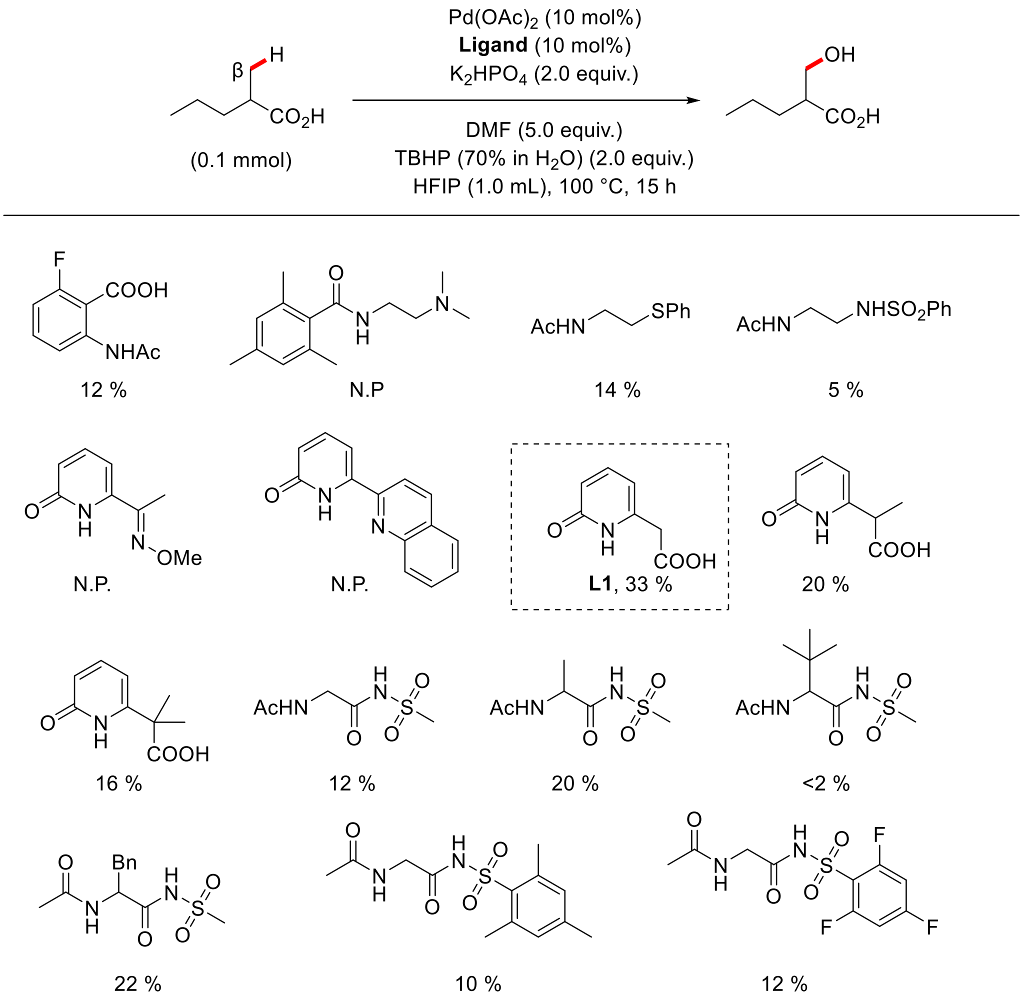

Scheme S1: Preliminary screening of different ligand classes

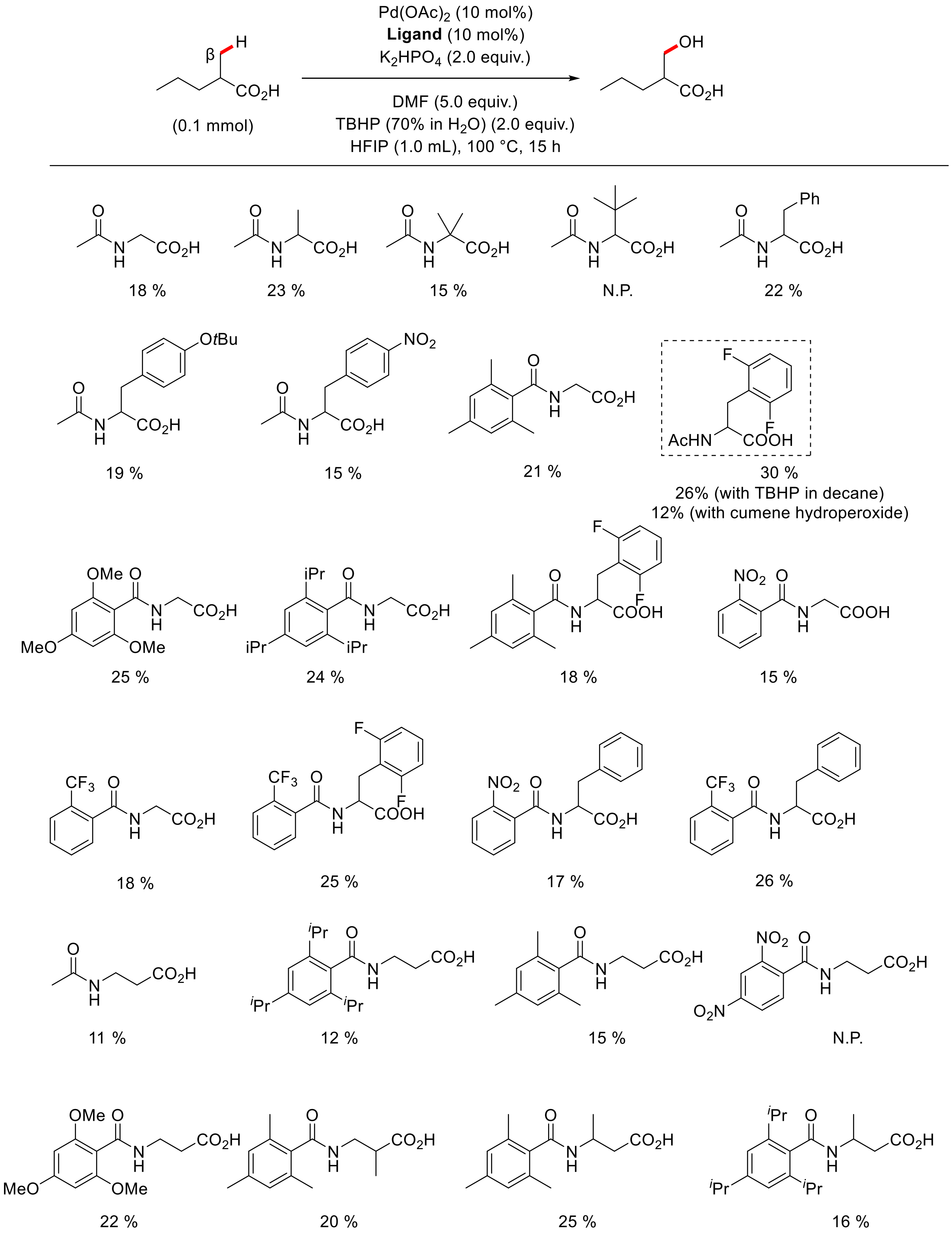

Scheme S2: Preliminary screening of amino acid derivatives

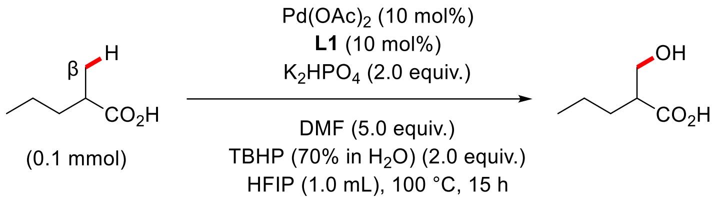

Entry	Variation	NMR-Yield (%)
1.	As above	33
2.	w/o $Pd(OAc)_2$	N.P
3.	w/o TBHP	N.P
4.	w/o K_2HPO_4	N.P
5.	w/o DMF	25
6.	DMF (7.5 equiv)	32
7.	DMF (2.5 equiv)	29

Scheme S3: Control experiments with L1

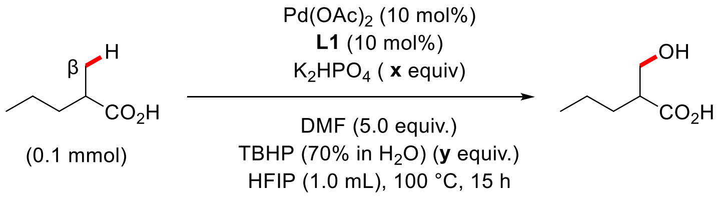

Entry	**x** (equiv)	**y** (equiv)	NMR-Yield (%)
1.	1.5	2.0	30
2.	2.0	2.0	33
3.	2.5	2.0	35 (conv. 62%)
4.	3.0	2.0	35 (conv. 58%)
5.	3.5	2.0	35 (conv. 56%)
6.	3.5	3.0	36
7.	3.5	4.0	38 (conv. 63%)
8.	3.5	5.0	40 (conv. 73%)

Scheme S4: Optimization of base and oxidant amount with L1

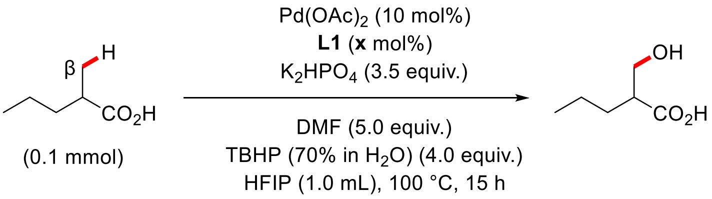

Entry	**x** (mol%)	NMR-Yield (%)
1.	10	38
2.	15	43
3.	20	45
4.	25	48
5.	30	46

Scheme S5: Screening of ligand (L1) loading

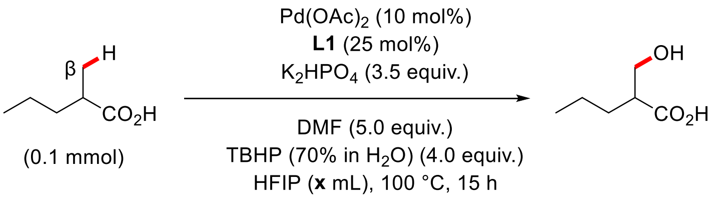

Entry	**x** (mL)	NMR-Yield (%)
1.	0.75	40
2.	1.0	48
3.	1.25	42
4.	1.5	40
5.	2.0	36

Scheme S6: Screening of solvent volume with L1

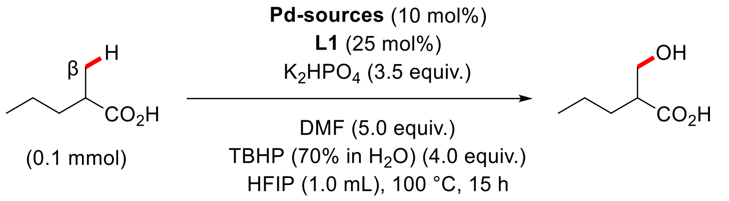

Entry	Pd-sources	NMR-Yield (%)
1.	$Pd(OAc)_2$	48
2.	$Pd(CF_3CO_2)_2$	42
3.	$Pd(CH_3CN)_2Cl_2$	42
4.	$PdCl_2$	43
5.	$Pd(acac)_2$	42

Scheme S7: Screening of different Pd-sources with L1

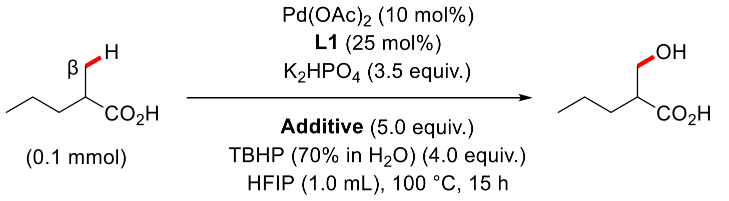

Entry	Additive	NMR-Yield (%)
1.	w/o	37
2.	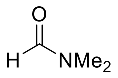	48
3.	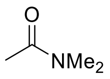	35
4.	CH_3CN	32
5.	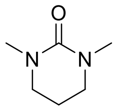	41

Scheme S8: Screening of additives with L1

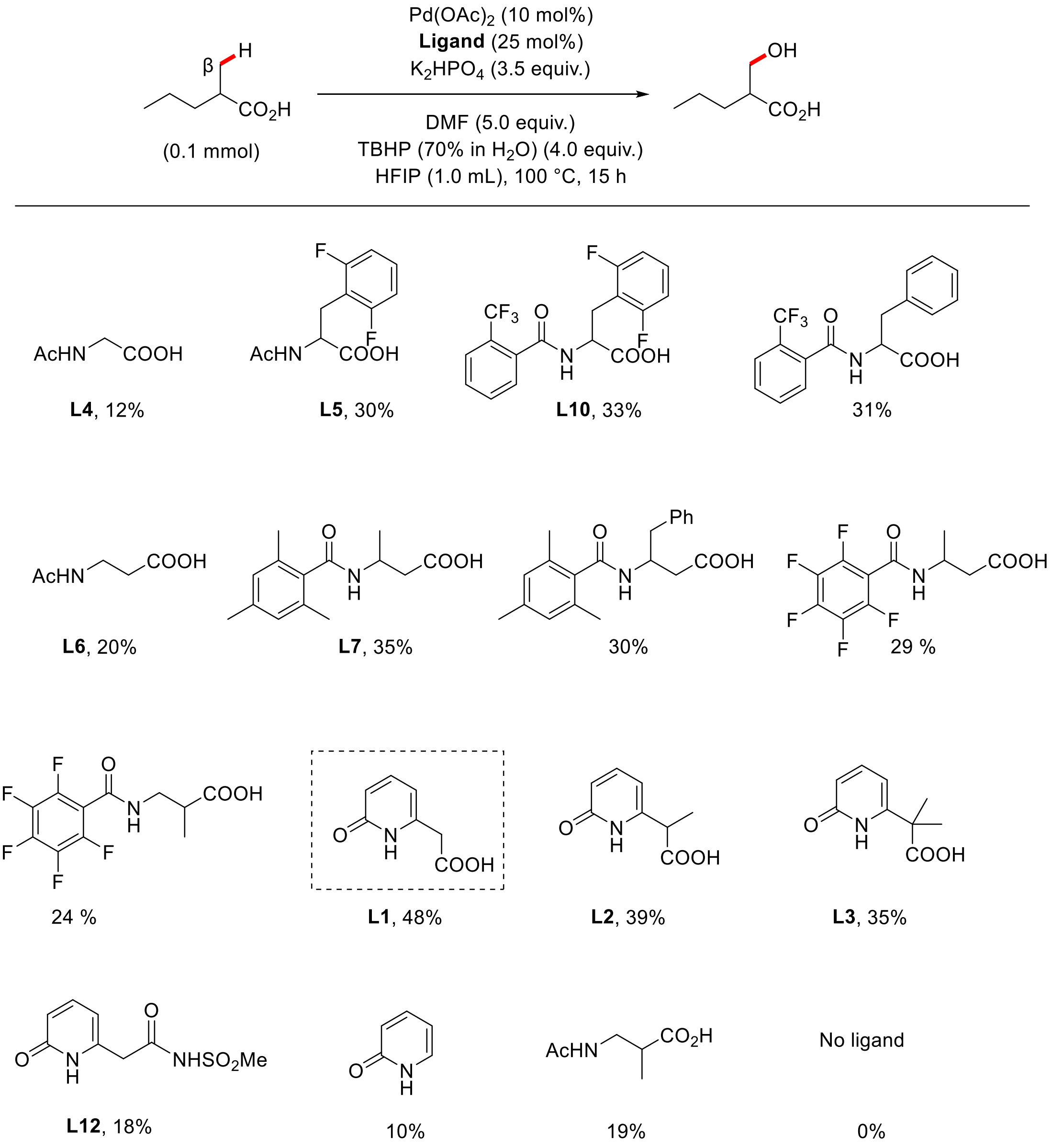

Scheme S9: Re-screening of ligands under the newly optimized conditions

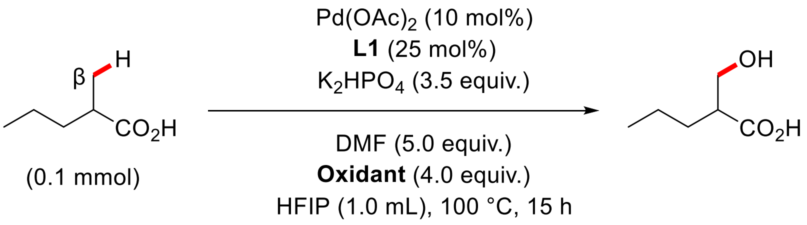

Entry	Oxidant	NMR-Yield (%)
1.	no oxidant	0
2.	TBHP (70% in H_2O)	48
3.	TBHP (in decane)	45
4.	^{t}BuOO^{t}Bu	0
5.	*m*-CPBA	0
6.	$Na_2CO_3 \cdot 1.5\ H_2O_2$	22
7.	H_2O_2 (50 wt% in H_2O)	13
8.	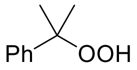 (CMHP)	28
9.	Oxone	0

Scheme S10: Re-screening of oxidants under the newly optimized conditions

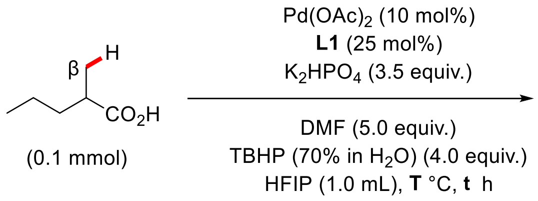

T (°C)	t (h)	NMR-Yield (%)
T = 80 °C	t = 24 h	45
	t = 48 h	44
	t = 72 h	43
T = 90°C	t = 24 h	41
	t = 48 h	45
	t = 72 h	46
T = 100 °C	t = 15 h	48
	t = 48 h	46
T = 110 °C	t = 15 h	43
	t = 48 h	43
T = 120 °C	t = 15 h	40
	t = 48 h	38

Scheme S11: Temperature and time screening with L1

List of challenging substrates

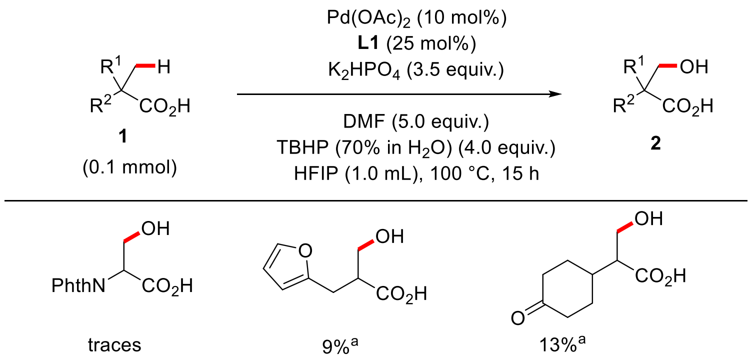

[a] ^{1}H NMR yields of corresponding benzyl ester were determined using CH_2Br_2 as an internal standard.

4. Synthesis of Starting Materials

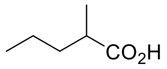

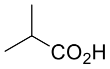

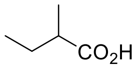

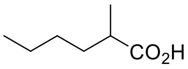

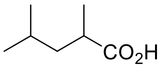

1a **1b** **1c** **1d** **1e**

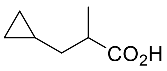

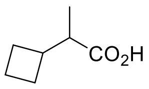

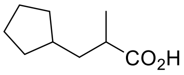

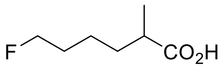

1f **1g** **1h** **1i**

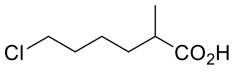

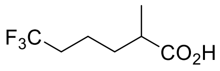

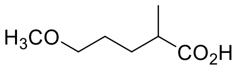

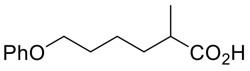

1j **1k** **1l** **1m**

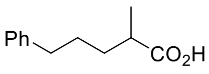

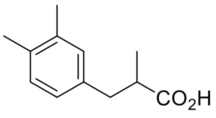

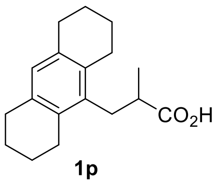

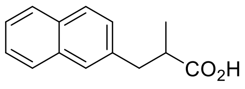

1n **1o** **1p** **1q**

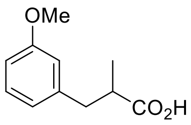

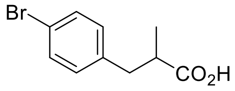

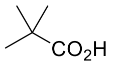

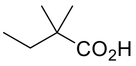

1r **1s** **1t** **1u**

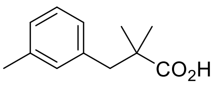

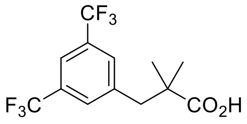

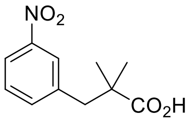

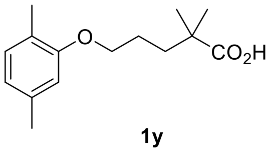

1v **1w** **1x** **1y**

Aliphatic carboxylic acids were obtained commercially available or synthesized following the literature procedure.[4-7]

6-fluoro-2-methylhexanoic acid

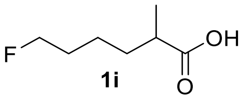

1i Following the literature reported procedure[8], using ^{n}BuLi (6.70 mL, 2.5M solution in hexane, 16.8 mmol, 2.1 equiv.), ${}^{i}Pr_2NH$ (2.50 mL, 17.6 mmol, 2.2 equiv.), propionic acid (0.59 g, 8.0 mmol, 1 equiv.), and 1-bromo-4-fluorobutane (1.5 g, 9.6 mmol, 1.1 equiv.) the target compound **1i** was obtained as a colorless oil (0.95 g, 6.4 mmol, 81%).

^{1}H NMR (500 MHz, $CDCl_3$): δ = 4.49 (t, *J* = 6.1 Hz, 1H), 4.40 (t, *J* = 6.0 Hz, 1H), 2.54 – 2.42 (m, 1H), 1.77 – 1.66 (m, 3H), 1.54 – 1.38 (m, 3H), 1.20 (d, *J* = 7.0 Hz, 3H) ppm.
^{13}C NMR (126 MHz, $CDCl_3$): δ = 183.1, 84.0 (d, *J* = 164.6 Hz), 39.4, 33.2, 30.4 (d, *J* = 19.8 Hz), 23.1 (d, *J* = 5.5 Hz), 17.0 ppm.

^{19}F NMR (471 MHz, $CDCl_3$): δ = -218.9 (s).

HRMS (ESI) m/z: $[M+H]^+$ Calcd for $C_7H_{14}FO_2$ 149.0977, Found 149.0977.

IR (cm^{-1}): 3696, 2965, 1700, 1414, 1372, 1261, 1225, 1055, 1033, 1010, 939, 764, 749.

7-chloro-2-methylheptanoic acid

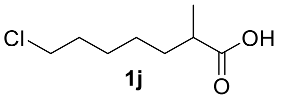

1j Following the literature reported procedure[8], using ^{n}BuLi (6.70 mL, 2.5M solution in hexane, 16.8 mmol, 2.1 equiv.), ${}^{i}Pr_2NH$ (2.50 mL, 17.6 mmol, 2.2 equiv.), propionic acid (0.59 g, 8.0 mmol, 1 equiv.), and 1-bromo-5-chloropentane (1.6 g, 8.8 mmol, 1.1 equiv.) the target compound **1j** was obtained as a colorless oil (1.0 g, 5.6 mmol, 70%).

^{1}H NMR (600 MHz, $CDCl_3$): δ = 3.53 (t, *J* = 6.7 Hz, 2H), 1.80 – 1.75 (m, 2H), 2.50 – 2.43 (m, 1H), 1.74 – 1.67 (m, 1H), 1.49 – 1.42 (m, 3H), 1.39 – 1.33 (m, 2H), 1.19 (d, *J* = 7.0 Hz, 3H) ppm.

^{13}C NMR (151 MHz, $CDCl_3$): δ = 183.3, 45.1, 39.4, 33.4, 32.5, 26.9, 26.6, 17.0 ppm.

HRMS (ESIpos) m/z: $[M+H]^+$ Calcd for $C_8H_{16}ClO_2$ 179.0838, Found 179.0839.

IR (cm^{-1}): 3696, 2964, 2869, 1699, 1455, 1415, 1277, 1223, 1055, 1032, 1012, 749, 652.

6,6,6-trifluoro-2-methylhexanoic acid

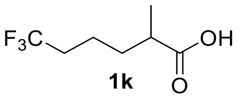

1k

Following the literature reported procedure[8], using ^{n}BuLi (6.70 mL, 2.5M solution in hexane, 16.8 mmol, 2.1 equiv.), $^{i}Pr_2NH$ (2.50 mL, 17.6 mmol, 2.2 equiv.), propionic acid (0.59 g, 8.0 mmol, 1 equiv.), and 4-bromo-1,1,1-trifluorobutane (1.8 g, 9.6 mmol, 1.1 equiv.) the target compound **1k** was obtained as a colorless oil (0.97 g, 5.3 mmol, 66%).

^{1}H NMR (500 MHz, $CDCl_3$): δ = 2.54 – 2.45 (m, 1H), 2.15 – 2.06 (m, 2H), 1.81 – 1.71 (m, 1H), 1.66 – 1.58 (m, 2H), 1.56 – 1.48 (m, 1H), 1.22 (d, *J* = 7.0 Hz, 3H) ppm.
^{13}C NMR (126 MHz, $CDCl_3$): δ = 182.7, 127.1 (q, *J* = 276.5 Hz), 39.2, 33.8 (q, *J* = 28.7 Hz), 32.5, 19.8 (d, *J* = 3.4 Hz), 16.9 ppm.

^{19}F NMR (471 MHz, $CDCl_3$): δ = -66.9 (s) ppm.

HRMS (EI) m/z: $[M]^+$ Calcd for $C_7H_{11}F_3O_2$ 184.0711, Found 184.0710.

IR (cm^{-1}): 3680, 2873, 2967, 1702, 1465, 1392, 1276, 1257, 1151, 1131, 1054, 1032, 749.

2-methyl-3-(1,2,3,4,5,6,7,8-octahydroanthracen-9-yl)propanoic acid

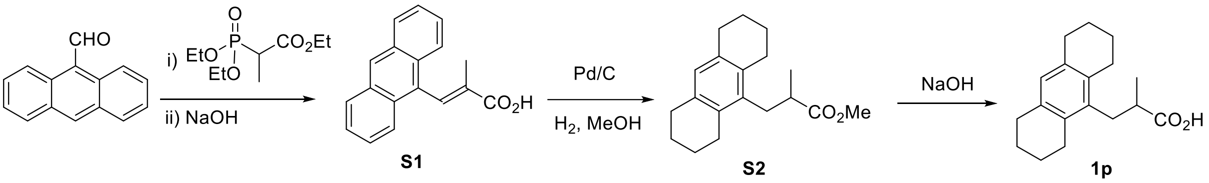

A Horner–Wadsworth–Emmons reaction[9] [using anthracene-9-carbaldehyde (2.0 g, 10 mmol, 1 equiv.), ethyl 2-(diethoxyphosphoryl)propanoate (3.20 g, 13.5 mmol, 1.35 equiv.) and NaH (315 mg, 13.1 mmol, 1.35 equiv.] and subsequent hydrolysis[2] afforded **S1** as a colorless solid (2.0 g, 7.6 mmol, 78%).[10]

Hydrogenation[11] of **S1** (530 mg, 2.02 mmol, 1 equiv.) in MeOH (20 mL) under H_2 atmosphere (50 bar) at 65 °C, led to the formation of **S2,** which was subsequently hydrolyzed[2] to afford the target compound **1p** as a colorless solid. (315 mg, 1.20 mmol, 60%).

^{1}H NMR (600 MHz, $CDCl_3$): δ = 7.77 (s, 1H), 3.56 (dd, *J* = 13.9, 5.1 Hz, 1H), 3.35 – 3.30 (m, 1H), 3.27 – 3.17 (m, 9H), 2.34 – 2.22 (m, 8H), 1.67 (d, *J* = 6.9 Hz, 3H) ppm.
^{13}C NMR (151 MHz, $CDCl_3$): δ = 182.5, 135.8, 134.8, 133.5, 128.9, 39.4, 31.3, 30.1, 27.1, 23.9, 23.0, 16.3 ppm.

HRMS (EI) m/z: $[M]^+$ Calcd for $C_{18}H_{24}O_2$ 272.1776, Found 272.1775.

IR (cm^{-1}): 2956, 2934, 1700, 1491, 1472, 1293, 1284, 1261, 1226, 941, 759, 749.

5. Scope Studies of the Hydroxylation Reaction

General Procedure A:

An oven dried 10 mL Schlenk tube was charged with $Pd(OAc)_2$ (4.5 mg, 0.020 mmol, 10 mol%), **L1** (7.6 mg, 0.050 mmol, 25 mol%), K_2HPO_4 (122 mg, 700 µmol, 3.5 equiv.), DMF (77 µL, 1.0 mmol, 5 equiv), TBHP (70% in H_2O, 116 µL, 800 µmol, 4 equiv.) carboxylic acid **1** (0.200 mmol, 1.0 equiv.) and HFIP (2.0 mL). The reaction mixture was stirred at 100 °C for 15 h in a preheated metal block. The reaction mixture was allowed to cool to rt and formic acid (0.2 mL) was added. The mixture was filtered through a pad of Celite® using CH_2Cl_2 (30 mL) to complete the elution and the volatiles were removed under reduced pressure. Cs_2CO_3 (425 mg, 1.30 mmol, 6.5 equiv.), NaI (15 mg, 0.10 mmol, 0.50 equiv.), benzyl bromide (118 µL, 1.00 mmol, 5.0 equiv.) and acetone (5 mL) were added and the resulting the mixture was stirred at rt for 24 h. The mixture was filtered through a pad of Celite® using CH_2Cl_2 (20 mL) as eluent and all volatiles were removed under reduced pressure. The resulting mixture was diluted with CH_2Cl_2 (10 mL) and was washed with H_2O (4× 20 mL), the organic layer was concentrated under reduced pressure and subsequent purification by silica gel column chromatography afforded the desired product in the form of benzyl ester.

Benzyl 2-(hydroxymethyl)pentanoate

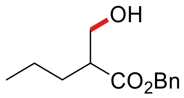

2a-Bn Following the general procedure **A**, using **1a** (23.2 mg) and pentane/Et_2O (65:35 v/v) as an eluent, the target compound **2a-Bn** was obtained as a colorless oil (20.7 mg, 93.0 µmol, 46%).

^{1}H-NMR (600 MHz, $CDCl_3$): δ = 7.38-7.31 (m, 5H), 5.17 (d, *J* = 12.5 Hz, 1H), 5.15 (d, *J* = 12.5 Hz, 1H), 3.80-3.73 (m, 2H), 2.67-2.62 (m, 1H), 1.68 – 1.61 (m, 1H), 1.55-1.48 (m, 1H), 1.38 – 1.32 (m, 2H), 0.90 (t, *J* = 7.3 Hz, 3H) ppm.

^{13}C-NMR (151 MHz, $CDCl_3$): δ = 175.4, 136.0, 128.7, 128.4, 128.2, 66.5, 63.2, 47.5, 30.7, 20.5, 14.1 ppm.

HRMS (ESIpos) m/z: $[M+H]^+$ Calcd for $C_{13}H_{19}O_3$ 223.1328, Found 223.1325.

Naphthalen-2-ylmethyl 3-hydroxy-2-methylpropanoate

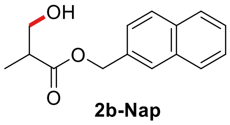

2b-Nap Following the general procedure **A**, using **1b** (17.6 mg), 2-(bromomethyl)naphthalene (220 mg, 1.00 mmol, 5 equiv.), and pentane/Et_2O (85:15 v/v) as an eluent, the target compound **2b-Nap** was obtained as a colorless oil (21.6 mg, 88.4 µmol, 44%).

^{1}H-NMR (600 MHz, $CDCl_3$): δ = 7.87 – 7.83 (m, 3H), 7.83 – 7.82 (m, 1H), 7.52 – 7.48 (m, 2H), 7.46 (dd, *J* = 8.4, 1.7 Hz, 1H), 5.33 (d, *J* = 1.5 Hz, 2H), 3.80 – 3.71 (m, 2H), 2.80 – 2.72 (m, 1H), 1.23 (d, *J* = 7.3 Hz, 3H) ppm.

^{13}C-NMR (151 MHz, $CDCl_3$): δ = 175.6, 133.3 (2C), 133.2, 128.6, 128.1, 127.9, 127.4, 126.5, 126.5, 125.8, 66.7, 64.7, 42.0, 13.6 ppm.

HRMS (EI) m/z: $[M]^+$: Calcd for $C_{15}H_{16}O_3$ 244.1099, Found 244.1097.

Benzyl 2-(hydroxymethyl)butanoate

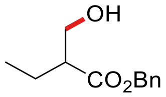

2c-Bn Following the general procedure **A**, using **1c** (20.4 mg) and pentane/Et_2O (70:30 v/v) as an eluent, the target compound **2c-Bn** was obtained as a colorless oil (17.6 mg, 84.6 µmol, 42%).

^{1}H-NMR (500 MHz, $CDCl_3$): δ = 7.38 – 7.32 (m, 5H), 5.19 (d, *J* = 12.4 Hz, 1H), 5.15 (d, *J* = 12.4 Hz, 1H), 3.84 – 3.74 (m, 2H), 2.59-2.54 (m, 1H), 1.76 – 1.58 (m, 2H), 0.94 (t, *J* = 7.5 Hz, 3H) ppm.

^{13}C-NMR (126 MHz, $CDCl_3$): δ = 175.3, 136.0, 128.7, 128.4, 128.3, 66.5, 62.9, 49.1, 21.8, 11.8 ppm.

HRMS (ESIpos) m/z: $[M+H]^+$ Calcd for $C_{12}H_{17}O_3$ 209.1172, Found 209.1173.

Benzyl 2-(hydroxymethyl)hexanoate

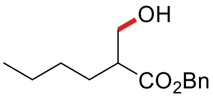

2d-Bn Following the general procedure **A**, using **1d** (26.0 mg) and pentane/Et_2O (60:40 v/v) as an eluent, the target compound **2d-Bn** was obtained as a colorless oil. (21.1 mg, 88.9 µmol, 44 %).

^{1}H-NMR (500 MHz, $CDCl_3$): δ = 7.38 – 7.32 (m, 5H), 5.18 (d, *J* = 12.4 Hz, 1H), 5.15 (d, *J* = 12.4 Hz, 1H), 3.80-3.72 (m, 2H), 2.65-2.60 (m, 1H), 1.71 – 1.62 (m, 1H), 1.59 – 1.51 (m, 1H), 1.35 – 1.24 (m, 4H), 0.87 (t, *J* = 7.5 Hz, 3H) ppm.

^{13}C-NMR (126 MHz, $CDCl_3$): δ = 175.4, 136.0, 128.7, 128.4, 128.3, 66.5, 63.3, 47.7, 29.4, 28.3, 22.7, 14.0 ppm.

HRMS (ESIpos) m/z: $[M+NH_4]^+$ Calcd for $C_{14}H_{24}O_3N$ 254.1750, Found 254.1751.

Benzyl 2-(hydroxymethyl)-4-methylpentanoate

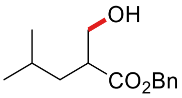

2e-Bn Following the general procedure **A**, using **1e** (26.0 mg) and pentane/Et_2O (60:40 v/v) as an eluent, the target compound **2e-Bn** was obtained as a colorless oil (19.8 mg, 0.88 mmol, 42%).

^{1}H-NMR (500 MHz, $CDCl_3$): δ = 7.35 (m, 5H), 5.16 (s, 2H), 3.75 (t, *J* = 5.4 Hz, 2H), 2.72 (m, 1H), 2.12 – 2.05 (m, 1H), 1.64 – 1.53 (m, 2H), 1.38 – 1.30 (m, 1H), 0.91 (d, *J* = 6.3 Hz, 3H), 0.88 (d, *J* = 6.3 Hz, 3H).

^{13}C-NMR (126 MHz, $CDCl_3$): δ = 175.7, 136.0, 128.7, 128.4, 128.2, 66.5, 63.7, 45.9, 37.6, 26.0, 22.7, 22.5 ppm.

HRMS (ESIpos) m/z: $[M+NH_4]^+$ Calcd for $C_{14}H_{24}NO_3$ 254.1750, Found 254.1744.

Benzyl 3-cyclopropyl-2-(hydroxymethyl)propanoate

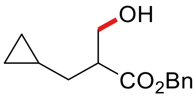

2f-Bn Following the general procedure **A**, using **1f** (25.6 mg) and pentane/Et_2O (60/30 v/v) as an eluent, the target compound **2f-Bn** was obtained as colorless oil (22.5 mg, 0.96 mmol, 48%).

^{1}H-NMR (500 MHz, $CDCl_3$): δ = 7.40 – 7.30 (m, 5H), 5.17 (s, 2H), 3.92 – 3.77 (m, 2H), 2.76 (m, 1H), 2.17 (t, J = 6.4 Hz, 1H), 1.60 – 1.48 (m, 2H), 0.73 – 0.64 (m, 1H), 0.49 – 0.36 (m, 2H), 0.09 – 0.00 (m, 2H) ppm.

^{13}C-NMR (126 MHz, $CDCl_3$): δ= 175.3, 135.9, 128.7, 128.4, 128.3, 66.6, 63.1, 48.2, 33.7, 8.9, 4.9, 4.6 ppm.

HRMS (ESIpos) m/z: $[M+NH_4]^+$ Calcd for $C_{14}H_{22}NO_3$ 252.1594, Found 252.1588.

Benzyl 2-cyclobutyl-3-hydroxypropanoate

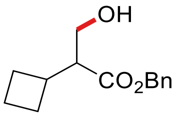

2g-Bn Following the general procedure **A**, using **1g** (22.8 mg) and pentane/Et_2O (60/30 v/v) as an eluent, the target compound **2g-Bn** was obtained as a colorless oil (21.1 mg,0.9 mmol, 45%).

^{1}H-NMR (500 MHz, $CDCl_3$): δ = 7.40 – 7.29 (m, 5H), 5.15 (q, J = 12.4 Hz, 2H), 3.71 (d, J = 5.4 Hz, 2H), 2.66 – 2.50 (m, 2H), 2.13 – 1.95 (m, 3H), 1.90 – 1.72 (m, 4H) ppm.

^{13}C-NMR (126 MHz, $CDCl_3$): δ = 174.5, 136.0, 128.7, 128.4, 128.3, 66.5, 61.5, 53.8, 34.8, 27.6, 27.4, 18.6 ppm.

HRMS (ESIpos) m/z: $[M+NH_4]^+$ Calcd for $C_{14}H_{22}NO_3$ 252.1594, Found 252.1585.

Benzyl 3-cyclopentyl-2-(hydroxymethyl)propanoate

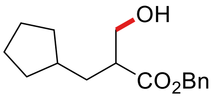

2h-Bn Following the general procedure **A**, using **1h** (31.2 mg) and pentane/Et_2O (60/30 v/v) as an eluent, the target compound **2h-Bn** was obtained as a colorless oil (27.2 mg, 1.0 mmol, 52%).

^{1}H-NMR (500 MHz, $CDCl_3$): δ = 7.40 – 7.28 (m, 5H), 5.21 – 5.12 (m, 2H), 3.82 – 3.72 (m, 2H), 2.73 – 2.64 (m, 1H), 2.10 (t, *J* = 6.3 Hz, 1H), 1.84 – 1.67 (m, 4H), 1.65 – 1.43 (m, 5H), 1.11 – 1.00 (m, 2H) ppm.

^{13}C-NMR (126 MHz, $CDCl_3$): δ = 175.6, 136.0, 128.7, 128.4, 128.3, 66.5, 63.6, 47.2, 38.0, 34.9, 32.8(3), 32.7(8), 25.1(9), 25.1(7) ppm.

HRMS (ESIpos) m/z: $[M+NH_4]^+$ Calcd for $C_{16}H_{26}NO_3$ 280.1907, Found 280.1901.

Benzyl 6-fluoro-2-(hydroxymethyl)hexanoate

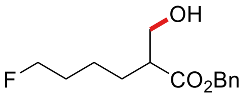

2i-Bn Following the general procedure **A**, using **1i** (29.6 mg) and pentane/Et_2O (60:40 v/v) as an eluent, the target compound **2i-Bn** was obtained as a colorless oil (22.5 mg, 82.3 µmol, 44%).

^{1}H-NMR (500 MHz, $CDCl_3$): δ = 7.38 – 7.32 (m, 5H), 5.19 (d, *J* = 12.3 Hz, 1H), 5.15 (d, *J* = 12.3 Hz, 1H), 4.39 (dt, *J* = 47.3, 6.0 Hz, 2H), 3.83 – 3.73 (m, 2H), 2.67 – 2.61 (m, 1H), 1.73 – 1.57 (m, 4H), 1.46 – 1.40 (m, 2H) ppm.

^{13}C-NMR (126 MHz, $CDCl_3$): δ = 175.1, 135.9, 128.7, 128.5, 128.3, 83.8 (d, *J* = 164.8), 66.6, 63.1, 47.6, 30.4 (d, *J* = 19.8 Hz), 28.2, 23.1 (d, *J* = 5.4 Hz) ppm.

^{19}F NMR (471 MHz, $CDCl_3$): δ = – 219.0 (s) ppm.

HRMS (ESIpos) m/z: $[M+NH_4]^+$ Calcd for $C_{14}H_{23}FO_3N$ 272.1656, Found 272.1655.

Benzyl 6-chloro-2-(hydroxymethyl)hexanoate

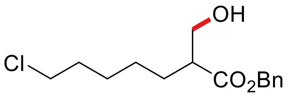

2j-Bn Following the general procedure **A**, using **1j** (32.9 mg) and pentane/Et_2O (60:40 v/v) as an eluent, the target compound **2j-Bn** was obtained as a colorless oil (23.2 mg, 85.8 µmol, 43%).

^{1}H-NMR (500 MHz, CDCl$_3$): δ = 7.38 – 7.33 (m, 5H), 5.19 (d, *J* = 12.3 Hz, 1H), 5.14 (d, *J* = 12.3 Hz, 1H), 3.81 – 3.72 (m, 2H), 3.48 (t, *J* = 6.7 Hz, 2H), 2.65-2.60 (m, 1H), 1.76 – 1.63 (m, 3H), 1.60 – 1.52 (m, 1H), 1.43 – 1.39 (m, 2H), 1.35 – 1.28 (m, 2H) ppm.

^{13}C-NMR (126 MHz, CDCl$_3$): δ = 175.2, 135.9, 128.8, 128.5, 128.4, 66.6, 63.2, 47.6, 45.0, 32.4, 28.4, 26.8, 26.5 ppm.

HRMS (ESIneg) m/z: $[M-H]^-$ Calcd for $C_8H_{14}O_3{}^{35}Cl$ 193.0631, Found 193.0630. The reported mass represents the respective acid **2j** resulted from the hydrolysis of **2j-Bn**.

Benzyl 6,6,6-trifluoro-2-(hydroxymethyl)hexanoate

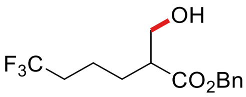

2k-Bn Following the general procedure **A**, using **1k** (36.8 mg) and pentane/Et_2O (65:35 v/v) as an eluent, the target compound **2k-Bn** was obtained as a colorless oil (23.9 mg, 82.4 µmol, 41%).

^{1}H-NMR (500 MHz, CDCl$_3$): δ = 7.40 – 7.33 (m, 5H), 5.19 (d, *J* = 12.3 Hz, 1H), 5.17 (d, *J* = 12.3 Hz, 1H), 3.80 – 3.77 (m, 2H), 2.66 – 2.59 (m, 1H), 2.10-2.01 (m, 2H), 1.79 – 1.70 (m, 1H), 1.68 – 1.54 (m, 3H) ppm.

^{13}C-NMR (126 MHz, CDCl$_3$): δ = 174.6, 135.7, 128.8, 128.6, 128.4, 127.0 (q, *J* = 276.4 Hz), 66.8, 63.0, 47.3, 33.7 (q, *J* = 28.6 Hz), 27.6, 19.9 (q, *J* = 3.2 Hz) ppm.

^{19}F NMR (471 MHz, CDCl$_3$): δ = – 66.8 (s) ppm.

HRMS (ESIpos) m/z: $[M+NH_4]^+$ Calcd for $C_{14}H_{21}F_3O_3N$ 308.1468, Found 308.1468.

Benzyl 2-(hydroxymethyl)-5-methoxypentanoate

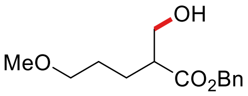

2l-Bn Following the general procedure **A**, using **1l** (29.2 mg) pentane/Et_2O (60/30 v/v) as an eluent, the target compound **2l-Bn** was obtained as a colorless oil (25.7 mg, 1.0 mmol, 51%).

^{1}H-NMR (500 MHz, $CDCl_3$): δ = 7.39 – 7.30 (m, 5H), 5.16 (s, 2H), 3.82 – 3.74 (m, 2H), 3.38 – 3.32 (m, 2H), 3.30 (s, 3H), 2.69 – 2.60 (m, 1H), 1.78 – 1.56 (m, 5H) ppm.

^{13}C-NMR (126 MHz, $CDCl_3$): δ = 175.1, 135.9, 128.7, 128.4, 128.3, 72.4, 66.6, 63.2, 58.7, 47.4, 27.3, 25.2 ppm.

HRMS (ESIpos) m/z: $[M+H]^+$ Calcd for $C_{14}H_{21}O_4$ 253.1434, Found 253.1429.

Benzyl 2-(hydroxymethyl)-5-phenoxypentanoate

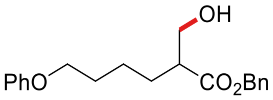

2m-Bn Following the general procedure **A**, using **1m** (44.5 mg) and pentane/Et_2O (60:40 v/v) as an eluent, the target compound **2m-Bn** was obtained as a colorless oil (31.7 mg, 96.5 µmol, 48%).

^{1}H-NMR (500 MHz, $CDCl_3$): δ = 7.37 – 7.31 (m, 5H), 7.30 – 7.26 (m, 2H), 6.95-6.92 (m, 1H), 6.89 – 6.85 (m, 2H), 5.19 (d, J = 12.3 Hz, 1H), 5.16 (d, J = 12.3 Hz, 1H), 3.91 (t, J = 6.4 Hz, 2H), 3.84 – 3.75 (m, 2H), 2.71 – 2.64 (m, 1H), 1.81 – 1.71 (m, 3H), 1.67-1.60 (m, 1H), 1.53-1.46 (m, 2H) ppm.

^{13}C-NMR (126 MHz, $CDCl_3$): δ = 175.2, 159.1, 135.9, 129.5, 128.7, 128.5, 128.3, 120.7, 114.6, 67.5, 66.6, 63.2, 47.6, 29.3, 28.3, 24.0 ppm.

HRMS (ESIpos) m/z: $[M+NH_4]^+$ Calcd for $C_{20}H_{28}O_4N$ 346.2012, Found 346.2010.

Benzyl 2-(hydroxymethyl)-5-phenylpentanoate

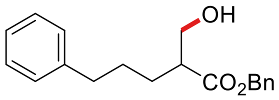

2n-Bn Following the general procedure **A**, using **1n** (38.4 mg) and pentane/Et_2O (60/30 v/v) as an eluent, the target compound **2n-Bn** was obtained as a colorless oil (26.8 mg, 0.9 mmol, 45%).

^{1}H-NMR (500 MHz, $CDCl_3$): δ = 7.39 – 7.30 (m, 5H), 7.28 – 7.23 (m, 2H), 7.19 – 7.15 (m, 1H), 7.13 – 7.08 (m, 2H), 5.21 – 5.10 (m, 2H), 3.83 – 3.68 (m, 2H), 2.69 – 2.64 (m, 1H), 2.59 (t, J = 7.3 Hz, 2H), 2.13 (s, 1H), 1.72 – 1.54 (m, 4H) ppm.

^{13}C-NMR (126 MHz, $CDCl_3$): δ = 175.2, 141.9, 135.9, 128.7, 128.4(9), 128.4(7), 128.3(9), 128.3, 126.0, 66.6, 63.2, 47.5, 35.8, 29.1, 28.2 ppm.

HRMS (ESIpos) m/z: $[M+H]^+$ Calcd for $C_{19}H_{23}O_3$ 299.1642, Found 299.1636.

Benzyl 2-(3,4-dimethylbenzyl)-3-hydroxypropanoate

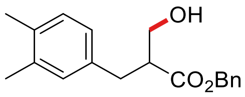

2o-Bn Following the general procedure **A**, using **1o** (38.4 mg) and pentane/Et_2O (60/30 v/v) as an eluent, the target compound **2o-Bn** was obtained as a colorless oil (23.8 mg, 0.8 mmol, 40%).

^{1}H-NMR (500 MHz, $CDCl_3$): δ = 7.36 – 7.31 (m, 3H), 7.27 – 7.23 (m, 2H), 7.02 (d, J = 7.6 Hz, 1H), 6.93 (d, J = 2.0 Hz, 1H), 6.89 (dd, J = 7.6, 1.9 Hz, 1H), 5.17 – 5.06 (m, 2H), 3.80 – 3.69 (m, 2H), 2.96 (dd, J = 13.1, 6.5 Hz, 1H), 2.92 – 2.85 (m, 1H), 2.80 (dd, J = 13.1, 7.9 Hz, 1H), 2.22 (s, 3H), 2.20 (s, 3H), 2.13 (t, J = 6.4 Hz, 1H) ppm.

^{13}C-NMR (126 MHz, $CDCl_3$): δ = 174.8, 136.8, 135.9, 135.8, 134.8, 130.4, 129.9, 128.7, 128.4, 128.3, 126.4, 66.6, 62.5, 49.4, 34.1, 19.9, 19.5 ppm.

HRMS (ESIpos) m/z: $[M+H]^+$ Calcd for $C_{19}H_{23}O_3$ 299.1642, Found 299.1635.

Benzyl 3-hydroxy-2-((1,2,3,4,5,6,7,8-octahydroanthracen-9-yl)methyl)propanoate

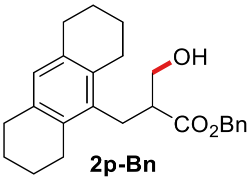

2p-Bn Following the general procedure **A**, using **1p** (54.4 mg) and pentane/Et_2O (60/30 v/v) as an eluent, the target compound **2p-Bn** was obtained as a colorless oil (22.7 mg, 0.6 mmol, 30%).

^{1}H-NMR (500 MHz, $CDCl_3$): δ = 7.40 – 7.26 (m, 3H), 7.24 – 7.19 (m, 2H), 6.73 (s, 1H), 5.16 – 5.05 (m, 2H), 3.83 – 3.70 (m, 2H), 3.07 – 2.97 (m, 1H), 2.98 – 2.84 (m, 2H), 2.69 (m, 8H), 2.27 (dd, J = 7.1, 5.7 Hz, 1H), 1.82 – 1.65 (m, 8H) ppm.

^{13}C-NMR (126 MHz, $CDCl_3$): δ = 175.4, 135.8, 135.3, 134.9, 133.4, 129.0, 128.7, 128.3, 128.0, 66.6, 62.6, 47.2, 30.1, 26.9, 26.8, 23.9, 23.0 ppm.

HRMS (ESIpos) m/z: $[M+NH_4]^+$ Calcd for $C_{25}H_{34}O_3N$ 396.2533, Found396.2524.

Benzyl 3-hydroxy-2-(naphthalen-2-ylmethyl)propanoate

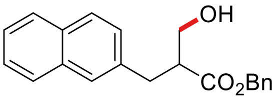

2q-Bn Following the general procedure **A**, using **1q** (42.8 mg) and pentane/Et_2O (60/30 v/v) as an eluent, the target compound **2q-Bn** was obtained as a colorless oil (23.0 mg, 0.72 mmol, 36%).

^{1}H-NMR (500 MHz, $CDCl_3$): δ = 7.83 – 7.77 (m, 1H), 7.76 – 7.72 (m, 2H), 7.63 – 7.59 (m, 1H), 7.49 – 7.40 (m, 2H), 7.33 – 7.21 (m, 4H), 7.19 – 7.15 (m, 2H), 5.18 – 5.05 (m, 2H), 3.85 – 3.74 (m, 2H), 3.19 (dd, J = 12.9, 6.4 Hz, 1H), 3.10 – 2.91 (m, 2H), 2.18 (s, 1H) ppm.

^{13}C-NMR (126 MHz, $CDCl_3$): δ = 174.6, 136.0, 135.6, 133.6, 132.4, 128.7, 128.3, 128.2, 128.1, 127.8, 127.7, 127.6, 127.4, 126.2, 125.7, 66.7, 62.5, 49.2, 34.7 ppm.

HRMS (ESIpos) m/z: $[M+H]^+$ Calcd for $C_{21}H_{21}O_3$ 321.1485, Found 321.1469.

Benzyl 3-hydroxy-2-(3-methoxybenzyl)propanoate

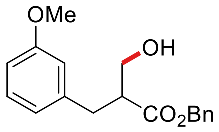

2r-Bn

Following the general procedure **A**, using **1r** (38.8 mg) and pentane/Et_2O (60/30 v/v) as an eluent, the target compound **2r-Bn** was obtained as a colorless oil (25.2 mg, 0.84 mmol, 42%).

^{1}H-NMR (500 MHz, $CDCl_3$): δ = 7.37 – 7.30 (m, 3H), 7.28 – 7.24 (m, 2H), 7.18 (t, J = 7.9 Hz, 1H), 6.78 – 6.70 (m, 3H), 5.18 – 5.09 (m, 2H), 3.82 – 3.70 (m, 5H), 3.02 (dd, J = 13.0, 6.3 Hz, 1H), 2.96 – 2.81 (m, 2H), 2.12 (s, 1H) ppm.

^{13}C-NMR (126 MHz, $CDCl_3$): δ = 174.6, 159.9, 140.1, 135.7, 129.7, 128.7, 128.4, 128.3, 121.4, 114.7, 112.1, 66.7, 62.5, 55.3, 49.2, 34.6 ppm.

HRMS (ESIpos) m/z: $[M+NH_4]^+$ Calcd for $C_{18}H_{24}O_4N$ 318.1700, Found 318.1690.

Benzyl 2-(4-bromobenzyl)-3-hydroxypropanoate

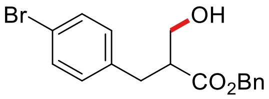

2s-Bn

Following the general procedure **A**, using **1s** (48.4 mg) and pentane/Et_2O (60/30 v/v) as an eluent, the target compound **2s-Bn** was obtained as a colorless oil (27.1 mg, 0.78 mmol, 39%).

^{1}H-NMR (500 MHz, $CDCl_3$): δ = 7.39 – 7.32 (m, 5H), 7.25 – 7.20 (m, 2H), 7.04 – 6.99 (m, 2H), 5.17 – 5.04 (m, 2H), 3.81 – 3.68 (m, 2H), 3.00 – 2.93 (m, 1H), 2.89 – 2.82 (m, 2H), 2.12 (s, 1H) ppm.

^{13}C-NMR (126 MHz, $CDCl_3$): δ = 174.3, 137.5, 135.6, 131.7, 130.9, 128.8, 128.5, 128.4, 120.6, 66.8, 62.4, 49.1, 34.0 ppm.

HRMS (ESIpos) m/z: $[M+NH_4]^+$ Calcd for $C_{17}H_{21}BrNO_3$ 366.0700, Found 366.0690.

Naphthalen-2-ylmethyl 3-hydroxy-2,2-dimethylpropanoate

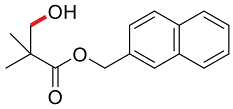

2t-Nap Following the general procedure **A**, using **1t** (20.4 mg), 2-(bromomethyl)naphthalene (220 mg, 1.00 mmol, 5 equiv.), and pentane/Et_2O (85:15 v/v) as an eluent, the target compound **2t-Nap** was obtained as a colorless oil (23.0 mg, 89.4 µmol, 45%).

^{1}H-NMR (600 MHz, $CDCl_3$): δ = 7.87 – 7.83 (m, 3H), 7.82 – 7.80 (m, 1H), 7.52 – 7.49 (m, 2H), 7.45 (dd, *J* = 8.4, 1.8 Hz, 1H), 5.31 (s, 2H), 3.60 (s, 2H), 1.25 (s, 6H) ppm.

^{13}C-NMR (151 MHz, $CDCl_3$): δ = 177.5, 133.4, 133.3, 133.2, 128.6, 128.1, 127.8, 127.2, 126.5, 126.4, 125.6, 69.8, 66.7, 44.5, 22.2 ppm.

HRMS (EI) m/z: $[M]^+$ Calcd for $C_{16}H_{18}O_3$ 258.1256, Found 258.1255.

Benzyl 2-(hydroxymethyl)-2-methylbutanoate

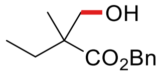

2u-Bn Following the general procedure **A**, using **1u** (23.2 mg) and pentane/Et_2O (65:35 v/v) as an eluent, the target compound **2u-Bn** was obtained as a colorless oil (17.0 mg, 76.4 µmol, 38%).

^{1}H-NMR (500 MHz, $CDCl_3$): δ = 7.39 – 7.31 (m, 5H), 5.16 (s, 2H), 3.74 (d, *J* = 11.2 Hz, 1H), 3.51 (d, *J* = 11.2 Hz, 1H), 1.71 – 1.53 (m, 2H), 1.20 (s, 3H), 0.85 (t, *J* = 7.6 Hz, 3H) ppm.

^{13}C-NMR (126 MHz, $CDCl_3$): δ = 177.1, 136.1, 128.7, 128.4, 128.1, 68.0, 66.5, 48.3, 28.7, 19.2, 8.7 ppm.

HRMS (ESIpos) m/z: $[M+H]^+$ Calcd for $C_{13}H_{19}O_3$ 223.1328, Found 223.1328.

Benzyl 3-hydroxy-2-methyl-2-(3-methylbenzyl)propanoate

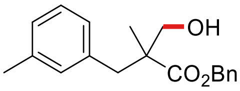

2v-Bn Following the general procedure **A**, using **1v** (38.4 mg) and pentane/Et_2O (65:35 v/v) as an eluent, the target compound **2v-Bn** was obtained as a colorless oil (20.3 mg, 68.2 µmol, 34%).

^{1}H-NMR (500 MHz, $CDCl_3$): δ = 7.39 – 7.28 (m, 5H), 7.14-7.11 (m, 1H), 7.03-7.00 (m, 1H), 6.93 – 6.90 (m, 2H), 5.14 (s, 2H), 3.63 (d, *J* = 11.3 Hz, 1H), 3.55 (d, *J* = 11.3 Hz, 1H), 2.94 (d, *J* = 13.3 Hz, 1H), 2.88 (d, *J* = 13.3 Hz, 1H), 2.29 (s, 3H), 1.16 (s, 3H) ppm.

^{13}C-NMR (126 MHz, $CDCl_3$): δ = 176.8, 137.8, 136.6, 135.8, 131.2, 128.7, 128.4, 128.2 (2C), 127.5(2), 127.4(7), 67.2, 66.6, 49.0, 41.1, 21.5, 19.5 ppm.

HRMS (ESIpos) m/z: $[M+NH_4]^+$ Calcd for $C_{19}H_{26}O_3N$ 316.1907, Found 316.1909.

Benzyl 2-(3,5-bis(trifluoromethyl)benzyl)-3-hydroxy-2-methylpropanoate

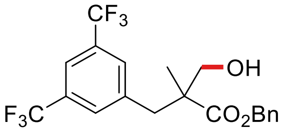

2w-Bn Following the general procedure **A**, using **1w** (62.8 mg) and pentane/Et_2O (60:40 v/v) as an eluent, the target compound **2w-Bn** was obtained as a colorless oil (34.7 mg, 82.7 µmol, 41%).

^{1}H-NMR (500 MHz, $CDCl_3$): δ = 7.75 (m, 1H), 7.65 (m, 2H), 7.39 – 7.34 (m, 3H), 7.31 – 7.27 (m, 2H), 5.16 (d, *J* = 12.3 Hz, 1H), 5.10 (d, *J* = 12.3 Hz, 1H), 3.68-3.65 (m, 1H), 3.50-3.47 (m, 1H), 3.11 (d, *J* = 13.6 Hz, 1H), 3.08 (d, *J* = 13.6 Hz, 1H), 1.16 (s, 3H) ppm.

^{13}C-NMR (126 MHz, $CDCl_3$): δ = 175.7, 139.5, 135.4, 131.5 (q, $^3J_{C\text{-}F}$ = 33.1 Hz), 130.6, 128.9, 128.7, 128.2, 123.4 (q, $^2J_{C\text{-}F}$ = 272.5 Hz), 120.9 – 120.8 (m), 67.0, 66.7, 49.0, 40.2, 19.4 ppm.

^{19}F NMR (471 MHz, $CDCl_3$): δ = – 63.3 (s) ppm

HRMS (EI) m/z: $[M]^+$ Calcd for $C_{20}H_{18}F_6O_3$ 420.1160, Found 420.1158.

Benzyl 3-hydroxy-2-methyl-2-(3-nitrobenzyl)propanoate

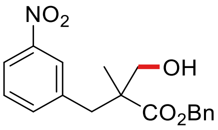

2x-Bn Following the general procedure **A**, using **1x** (44.6 mg) and pentane/Et_2O (50:50 v/v) as an eluent, the target compound **2x-Bn** was obtained as a colorless oil (27.5 mg, 83.6 µmol, 42%).

^{1}H-NMR (500 MHz, $CDCl_3$): δ = 8.08 – 8.01 (m, 2H), 7.47-7.41 (m, 1H), 7.39 – 7.33 (m, 4H), 7.33 – 7.29 (m, 2H), 5.16 (s, 2H), 3.65 (d, *J* = 11.3 Hz, 1H), 3.53 (d, *J* = 11.3 Hz, 1H), 3.06 (s, 2H), 1.17 (s, 3H) ppm.

^{13}C-NMR (126 MHz, $CDCl_3$): δ = 175.9, 148.2, 138.9, 136.7, 135.5, 129.2, 128.8, 128.6, 128.4, 125.2, 122.0, 67.0, 66.9, 48.9, 40.4, 19.4. ppm.

HRMS (ESIpos) m/z: $[M+NH_4]^+$ Calcd for $C_{18}H_{23}N_2O_5$ 347.1601, Found 347.1597.

Benzyl 5-(2,5-dimethylphenoxy)-2-(hydroxymethyl)-2-methylpentanoate

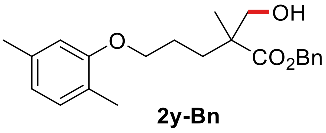

2y-Bn Following the general procedure **A**, using **1y** (50.1 mg) and pentane/Et_2O (70:30 v/v) as an eluent, the target compound **2y-Bn** was obtained as a colorless solid (36.1 mg, 0.101 mol, 51%).

^{1}H-NMR (500 MHz, $CDCl_3$): δ = 7.37 – 7.32 (m, 5H), 7.01– 6.98 (m, 1H), 6.66 (d, *J* = 6.5 Hz, 1H), 6.59 (s, 1H), 5.16 (s, 2H), 3.91 – 3.85 (m, 2H), 3.78 (d, *J* = 11.3 Hz, 1H), 3.58 (d, *J* = 11.3 Hz, 1H), 2.30 (s, 3H), 2.15 (s, 3H), 1.83 – 1.73 (m, 4H), 1.26 (s, 3H) ppm.

^{13}C-NMR (126 MHz, $CDCl_3$): δ = 176.8, 157.0, 136.6, 136.0, 130.5, 128.8, 128.4, 128.1, 123.7, 120.9, 112.1, 68.3, 67.9, 66.6, 47.8, 32.5, 24.6, 21.5, 19.8, 15.9 ppm.

HRMS (ESIpos) m/z: $[M+NH_4]^+$ Calcd for $C_{22}H_{32}O_4N$ 374.2325, Found 374.2319.

Large scale reaction

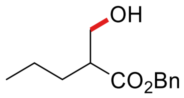

2a-Bn An oven dried 150 mL Schlenk tube was charged with $Pd(OAc)_2$ (23 mg, 0.10 mmol, 10 mol%), **L1** (38 mg, 0.25 mmol, 25 mol%), K_2HPO_4 (610 mg, 3.50 mmol, 3.5 equiv.), DMF (385 µL, 5.00 mmol, 5 equiv), TBHP (70% in H_2O, 580 µL, 4.00 mmol, 4 equiv.) carboxylic acid **1a** (116 mg, 1.0 mmol, 1.0 equiv.) and HFIP (10 mL). The reaction mixture was stirred at 100 °C for 15 h in a preheated metal block. The reaction mixture was allowed to cool to rt and formic acid (0.5 mL) was added. The mixture was filtered through a pad of Celite® using CH_2Cl_2 (35 mL) to complete the elution and the volatiles were removed under reduced pressure. Cs_2CO_3 (2.1 g, 6.5 mmol, 6.5 equiv.), NaI (75 mg, 0.50 mmol, 0.50 equiv.), benzyl bromide (580 µL, 5.00 mmol, 5.0 equiv.) and acetone (25 mL) were added and the resulting the mixture was stirred at rt for 24 h. The mixture was filtered through a pad of Celite® using CH_2Cl_2 (35 mL) as eluent and all volatiles were removed under reduced pressure. The resulting mixture was diluted with CH_2Cl_2 (20 mL) and was washed with H_2O (4× 30 mL), the organic layer was concentrated under reduced pressure and subsequent purification by silica gel column chromatography using pentane/Et_2O (65:35 v/v) as an eluent afforded the desired product **2a-Bn** as a colorless oil (91.1 mg, 0.409 mmol, 41%).

6. Preliminary Mechanistic Investigations

Control Experiment A: An oven dried 10 mL Schlenk tube was charged with $Pd(OAc)_2$ (2.3 mg, 0.010 mmol, 10 mol%), **L1** (3.8 mg, 0.025 mmol, 25 mol%), K_2HPO_4 (61 mg, 0.35 mmol, 3.5 equiv.), DMF (38.5 µL, 0.500 mmol, 5 equiv), TBHP (70% in H_2O, 58 µL, 0.40 mmol, 4 equiv.), **3u** (11.6 mg, 0.100 mmol, 1.0 equiv.), and HFIP (1.0 mL). The reaction mixture was stirred at 100 °C for 15 h in a preheated metal block. The reaction mixture was allowed to cool to room temperature and formic acid (0.1 mL) was added. The mixture was filtered through a pad of Celite® using CH_2Cl_2 (30 mL) to complete the elution. All volatiles were removed under reduced pressure and the crude reaction mixture was analyzed via ^1H NMR using CH_2Br_2 as internal standard.

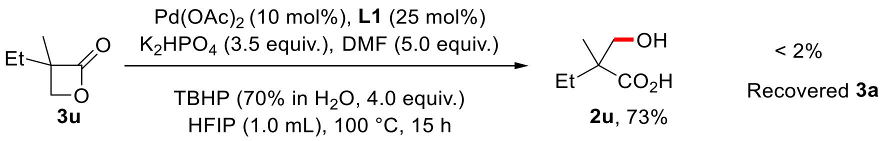

Control Experiment B: An oven dried 10 mL Schlenk tube was charged with $Pd(OAc)_2$ (2.3 mg, 0.010 mmol, 10 mol%), **L1** (3.8 mg, 0.025 mmol, 25 mol%), K_2HPO_4 (61 mg, 0.35 mmol, 3.5 equiv.), DMF (38.5 µL, 0.500 mmol, 5 equiv), TBHP (70% in H_2O, 58 µL, 0.40 mmol, 4 equiv.), **3y** (25.0 mg, 0.100 mmol, 1.0 equiv.), and HFIP (1.0 mL). The reaction mixture was stirred at 100 °C for 15 h in a preheated metal block. The reaction mixture was allowed to cool to room temperature and formic acid (0.1 mL) was added. The mixture was filtered through a pad of Celite® using CH_2Cl_2 (30 mL) to complete the elution. All volatiles were removed under reduced pressure and the crude reaction mixture was analyzed via ^1H NMR using CH_2Br_2 as internal standard.

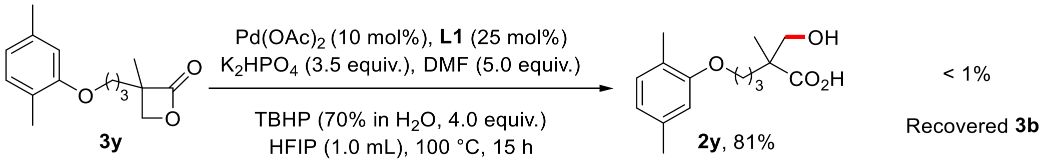

3u and **3y** were prepared following the literature procedure reported by Yu *et al.*[7]

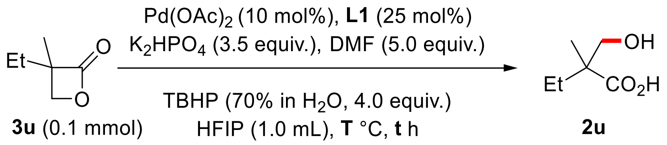

T (°C)	t	2u (%)	Recovered 3u (%)
T = 60	t = 1 h	18	81
	t = 2 h	25	68
	t = 3 h	46	42
	t = 4 h	62	26

Scheme S12: Hourly monitoring of the decomposition of 3u

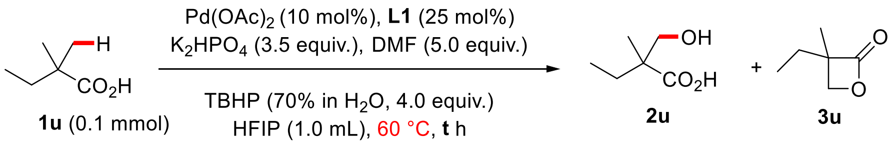

Entry	t	NMR-Yield (%) 2u	3u
1.	1 h	30	< 2%
2.	2 h	42	< 2%
3.	3 h	45	< 1%
4.	4 h	46	< 1%

Scheme S13: Hourly monitoring of the hydroxylation reaction of 1u

After establishing that substantial amounts of **3u** remain detectable even after 4 h of reaction time at 60 °C (Scheme S12), we monitored the hydroxylation of **1u** under these conditions. Even after 1 h 30% of product **2u** could be detected, which increased to 46% after 4 h. During this time no accumulation of **3u** could be detected (Scheme S13). Based on the timeframe required for hydrolysis established in Scheme S12, the product detected in Scheme S13 cannot have formed primarily through **3u** as an intermediate, since otherwise substantial amounts of this compound would have to be detectable. These experiments allow us to rule out *path B* as major pathway for product formation. Notably, while we had to reduce the reaction temperature

to ensure the detectability of **3u**, through the observation that 46% of the desired compound could be obtained after 4 h at 60°C our results clearly show that the barrier for the product formation through *path A* is lower than for the formation of the β-lactone. Since both of these steps take place as unimolecular processes from the same reaction intermediate, it seems highly unlikely that the reaction mechanism would change when the reaction is performed at a higher temperature.

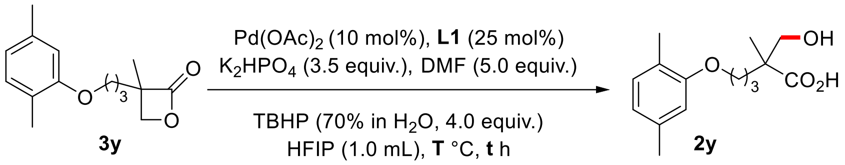

T (°C)	t	2y (%)	Recovered 3y (%)
T = 100	t = 30 min	82	0
	t = 1 h	81	0
T = 80	t = 1 h	82	0
T = 60	t = 1 h	42	56
	t = 2 h	62	33
	t = 4 h	80	< 8

Scheme S14: Hourly monitoring of the decomposition of 3y

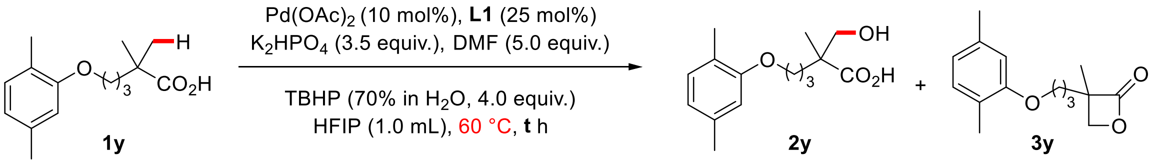

Entry	t	NMR-Yield (%) 2y	3y
1.	1 h	18	59
2.	2 h	32	42
3.	3 h	41	30
4.	4 h	51	18

Scheme S15: Hourly monitoring of the hydroxylation reaction of 1y

After identifying the suitable conditions where **3y** remains partially stable (Scheme S14), the hydroxylation of **1y** was investigated. Interestingly, after 1 h, a substantial accumulation of **3y** was observed, which gradually decomposes to **2y** with time (Scheme S15), indicating that **1y** follows *path B.* The combined results from Scheme S12-S15 establish that different pathways are operative for the formation of **2u** and **2y**. The question arises, which of these pathways is relevant for the majority of the scope. We hypothesize that the results obtained for the hydroxylation of **2u** are representative for the vast majority of compounds, while gemfibrozil **1y** constitutes an outlier. This hypothesis is based on previous observations, where gemfibrozil has repeatedly behaved differently from all other compounds studied.

It is worth highlighting that **1y** is known to have very strong tendency to form β-lactone via C–O RE from high-valent Pd (IV) species as reported previously by Yu lab,[7] where the authors reported yields of β-lactone **3y** much above the average yields. The tendency of **1y** to form β-lactone product **3y** is so strong, that we could recently observe its formation under the conditions we have optimized for β-C(sp^3)–H fluorination (Scheme S16),[1] where all other substrates gave fluorination selectively.

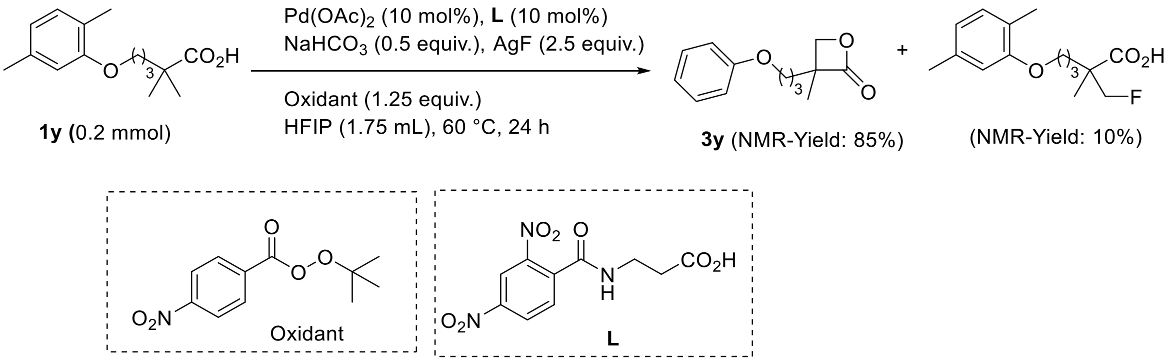

Scheme S16: β-C(sp^3)–H fluorination of gemfibrozil

These observations indicate that gemfibrozil (**1y**), has a unique propensity to undergo β-lactone formation even when other substrates prefer a different pathway. We therefore propose that this is also the case in our present work and therefore propose that the vast majority of compounds undergoes hydroxylation through *path A*, while gemfibrozil as an outlier follows *path B.*

Notably, Yu's study also showed that the β-lactone formation remains limited to α-quaternary carboxylic acids, which corroborates to our conclusion that *path A* is operative and at least for all α-non-quaternary substrates the direct formation of hydroxylated products via *path A* can be concluded. Together with the experimental evidence presented above pointing towards *path A* for simple α-quaternary substrates such as **1u** the classification of gemfibrozil as mechanistic outlier is strongly supported.

Investigations into the possible role of H_2O and O_2 as a source of hydroxyl group

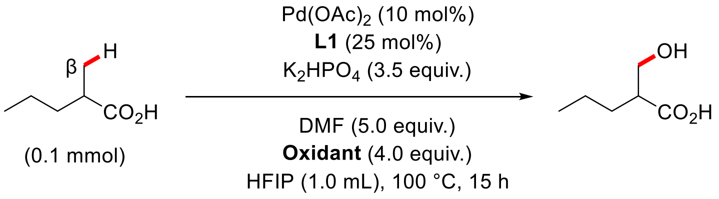

Entry	Oxidant	NMR-Yield (%)
1.	TBHP (70% in H_2O)	48%
2.	TBHP (in decane)	45%

Scheme S17: Comparison of aqueous and anhydrous TBHP under the optimized conditions

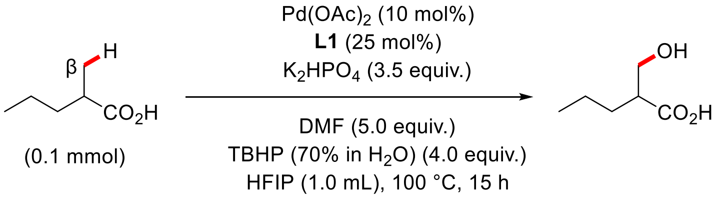

Entry	Conditions	NMR-Yield (%)
1.	As above (under air)	48%
2.	Degassed[a] HFIP under N_2	47%

Scheme S18: Influence of inert atmosphere on the optimized conditions. [a]HFIP was degassed using the freeze-pump-thaw technique.

Experiments to probe the possibility of an S_N2-like reaction in *path A*:

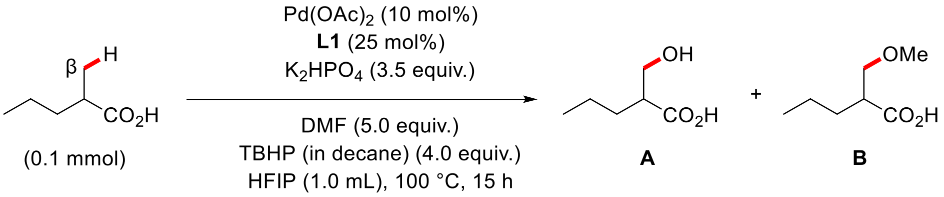

Entry	Additive	**A** (NMR-Yield)	**B** (NMR-Yield)
1.	None	45%	–
2.	MeOH (0.5 equiv.)	44%	0%
3.	MeOH (1.0 equiv.)	43%	0%

Scheme S19: Screening of MeOH as additive with anhydrous TBHP

We proceeded to investigate whether an S_N2-like reaction[12] is operative in *path A*.

We argued that the presence of a competing nucleophilic species, such as MeOH, should result in the formation of **B** (Scheme S19) if an S_N2-like attack at carbon bound to the high-valent Pd(IV) is involved in product formation. Additionally, since anhydrous TBHP was shown to give equally good results (Scheme S17), we argued that when using this oxidant and adding stoichiometric amounts of MeOH, there would be more MeOH than water present in the reaction mixture, suggesting that if these two nucleophiles were competing in product formation substantial amounts of **B** should be detectable. However, in the presence of 0.5 or 1.0 equiv. of MeOH, product **A** was observed (Entries 2-3) and the reaction remained virtually unaffected by the presence of MeOH (Entry 1). This finding strongly suggests that the product is formed by a direct reductive elimination from the Pd(IV) species containing the activated acid and a hydroxide ligand rather than involving a backside attack of dissociated water.

Experiments to probe product poisoning of the catalyst:

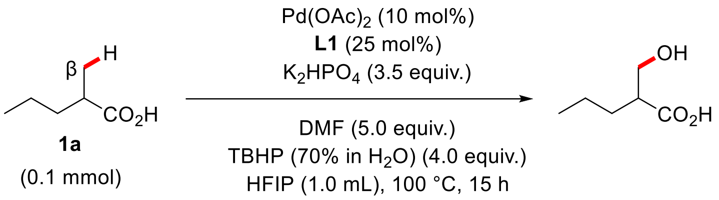

Entry	Additive	NMR-Yield (%)
1.	None	48
2.	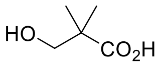 (**2t**, 0.25 equiv.)	40
3.	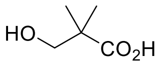 (**2t**, 0.5 equiv.)	32
4.	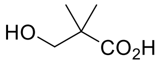 (**2t**, 1.0 equiv.)	18

Scheme S20: Monitoring of hydroxylation reaction of **1a** in the presence of **2t**.

Experiments to prevent catalyst poisoning:

To alleviate the catalyst poisoning, a series of boron additives was tested (see Scheme S21) due to their well-known ability to form cyclic boronate esters with diols and related structures.[13, 14] However, the starting material **1a** was found to decompose in the presence of boron additives, and neither **2a** nor its corresponding cyclic boronic esters were detected (Entry 2-4).

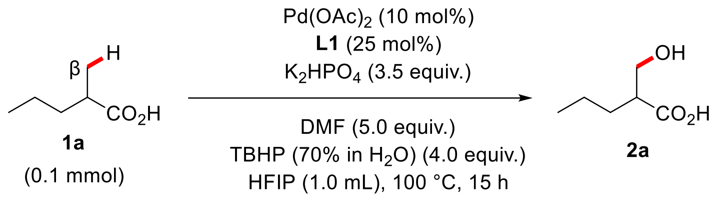

Entry	Additive	2a	Decomp. 1a (%)
1.	None	48	74
2.	$B(OH)_3$ (1.0 equiv.)	Not detected	77
3.	$B(OMe)_3$ (1.0 equiv.)	Not detected	73
4.	$PhB(OH)_2$ (1.0 equiv.)	Not detected	74

Scheme S21: Screening of boron additives for the hydroxylation of **1a**. The reactions were analyzed by ^1H NMR analysis of the crude reaction mixture using CH_2Br_2 as an internal standard.

7. References

(1) Mal, S.; Jurk, F.; Hiesinger, K.; van Gemmeren, M. Pd-catalysed direct β-C(sp^3)–H fluorination of aliphatic carboxylic acids. *Nat. Synth.* **2024**, *3*, 1292-1298.
(2) Farizyan, M.; Mondal, A.; Mal, S.; Deufel, F.; van Gemmeren, M. Palladium-Catalyzed Nondirected Late-Stage C–H Deuteration of Arenes. *J. Am. Chem. Soc.* **2021**, *143*, 16370-16376.
(3) Li, Z.; Park, H. S.; Qiao, J. X.; Yeung, K.-S.; Yu, J.-Q. Ligand-Enabled C–H Hydroxylation with Aqueous H_2O_2 at Room Temperature. *J. Am. Chem. Soc.* **2022**, *144*, 18109-18116.
(4) Ghosh, K. K.; van Gemmeren, M. Pd-Catalyzed β-C(sp^3)−H Arylation of Propionic Acid and Related Aliphatic Acids. *Chem. Eur. J.* **2017**, *23*, 17697-17700.
(5) Ghiringhelli, F.; Uttry, A.; Ghosh, K. K.; van Gemmeren, M. Direct β- and γ-C(sp^3)−H Alkynylation of Free Carboxylic Acids. *Angew. Chem. Int. Ed.* **2020**, *59*, 23127-23131.
(6) Zhuang, Z.; Herron, A. N.; Fan, Z.; Yu, J.-Q. Ligand-Enabled Monoselective β-C(sp^3)–H Acyloxylation of Free Carboxylic Acids Using a Practical Oxidant. *J. Am. Chem. Soc.* **2020**, *142*, 6769-6776.
(7) Zhuang, Z.; Yu, J.-Q. Lactonization as a general route to β-C(sp^3)–H functionalization. *Nature* **2020**, *577*, 656-659.
(8) Uttry, A.; Mal, S.; van Gemmeren, M. Late-Stage β-C(sp3)–H Deuteration of Carboxylic Acids. *J. Am. Chem. Soc.* **2021**, *143*, 10895-10901.
(9) Srikrishna, A.; Babu, R. R. Total synthesis of (±)-β-chamigrene and (±)-laurencenone C via Ireland ester Claisen rearrangement and an intramolecular type II carbonyl ene reaction sequence. *Tetrahedron* **2008**, *64*, 10501-10506.
(10) Rosowsky, A.; Papathanasopoulos, N.; Lazarus, H.; Foley, G. E.; Modest, E. J. Cysteine scavengers. 2. Synthetic .alpha.-methylenebutyrolactones as potential tumor inhibitors. *J. Med. Chem.* **1974**, *17*, 672-676.
(11) Sajiki, H.; Ikawa, T.; Hattori, K.; Hirota, K. A remarkable solvent effect toward the Pd/C-catalyzed cleavage of silyl ethers. *Chem. Commun.* **2003**, 654-655.
(12) Dick, A. R.; Hull, K. L.; Sanford, M. S. A Highly Selective Catalytic Method for the Oxidative Functionalization of C−H Bonds. *J. Am. Chem. Soc.* **2004**, *126*, 2300-2301.
(13) Liu, Y.; Brown, M. K. Photosensitized [2 + 2]-Cycloadditions of Dioxaborole: Reactivity Enabled by Boron Ring Constraint Strategy. *J. Am. Chem. Soc.* **2023**, *145*, 25061-25067.
(14) Akgun, B.; Hall, D. G. Boronic Acids as Bioorthogonal Probes for Site-Selective Labeling of Proteins. *Angew. Chem. Int. Ed.* **2018**, *57*, 13028-13044.

8. NMR Spectra

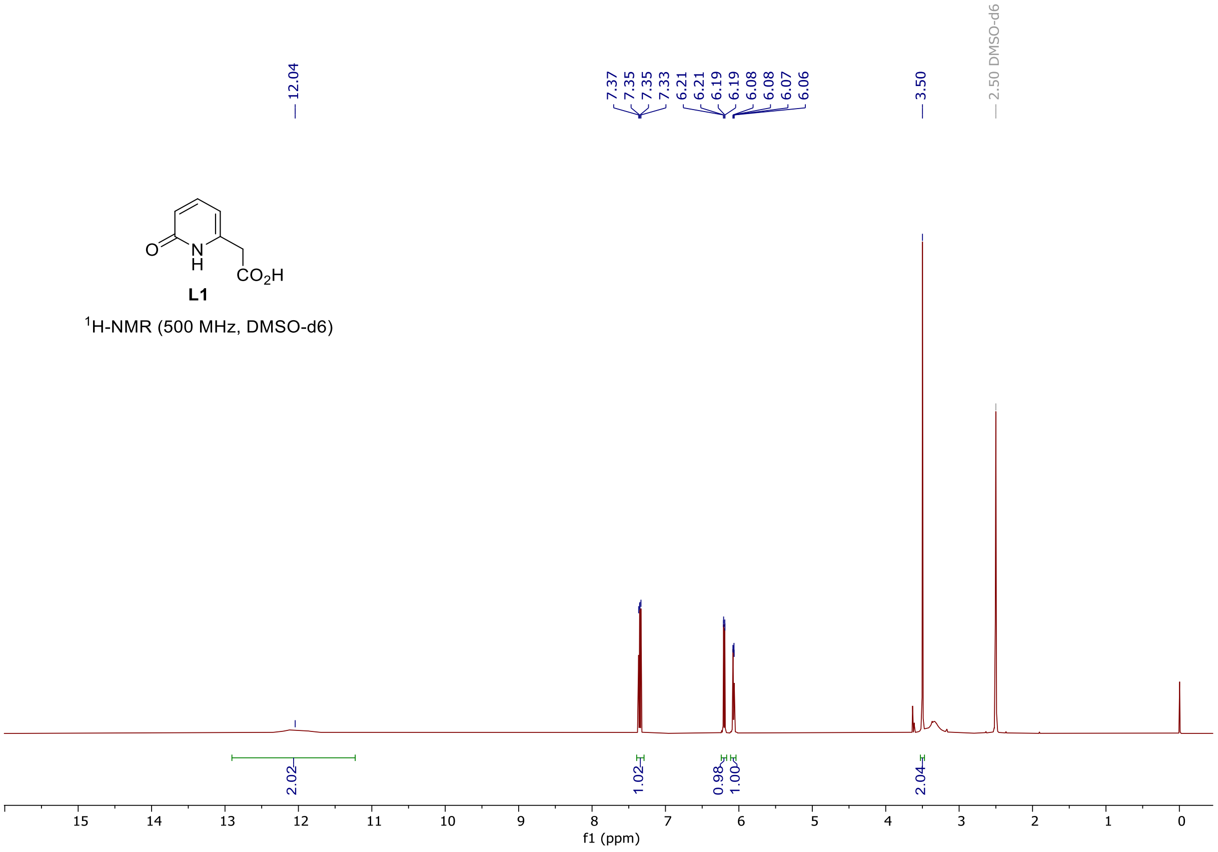

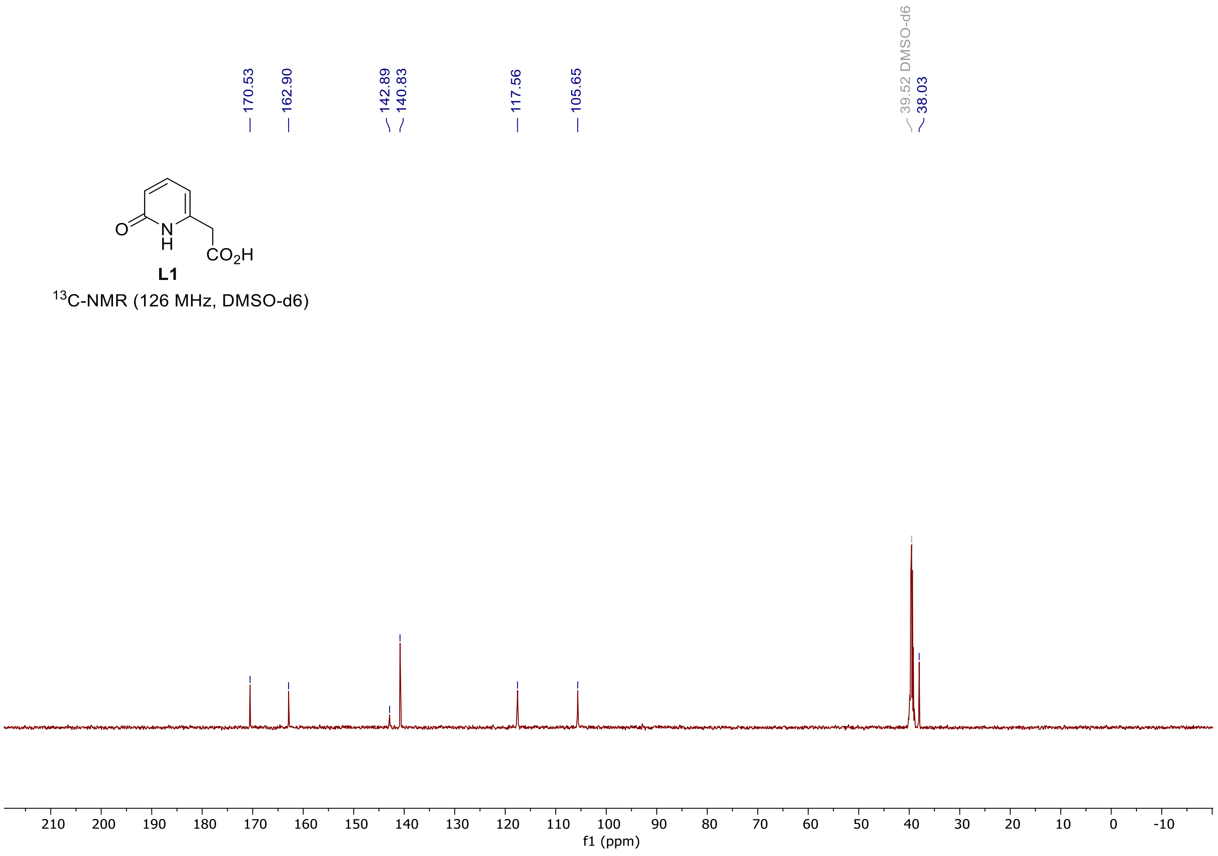

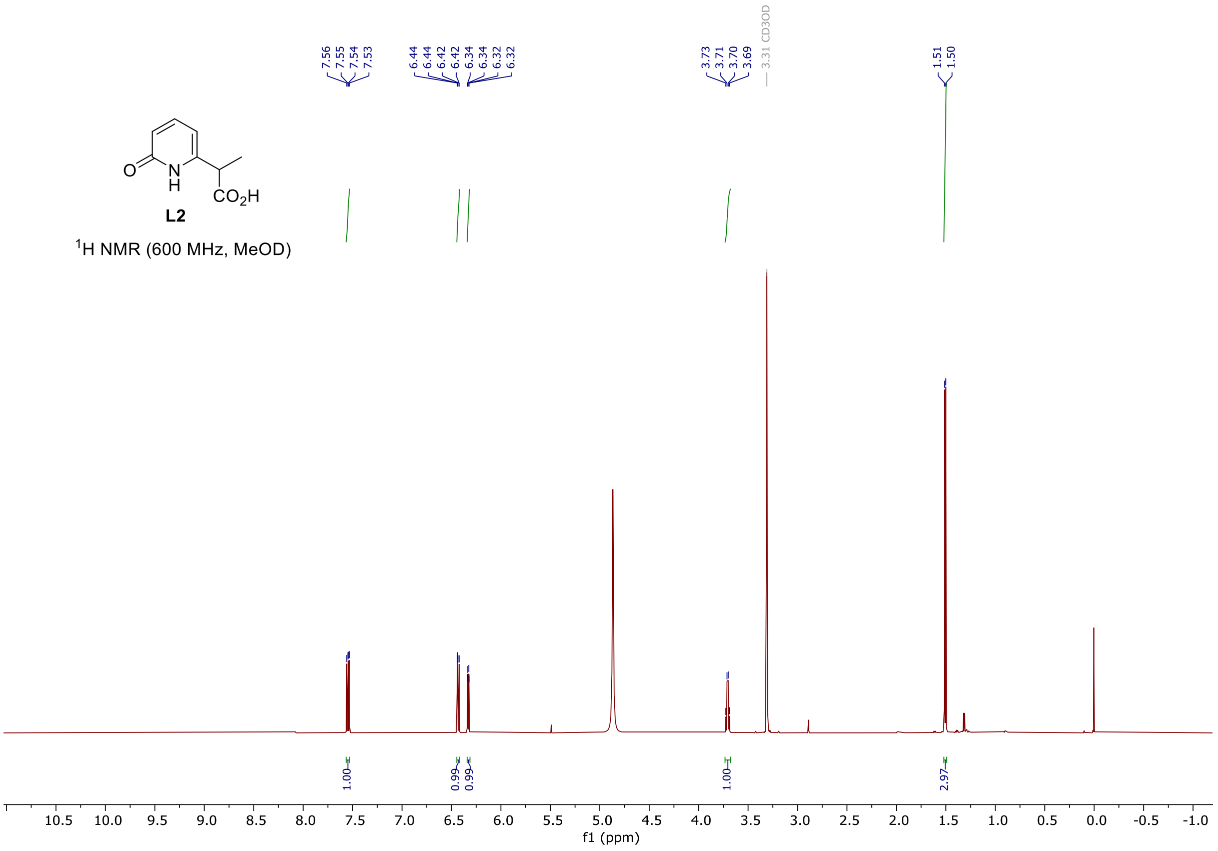

L2

^{1}H NMR (600 MHz, MeOD)

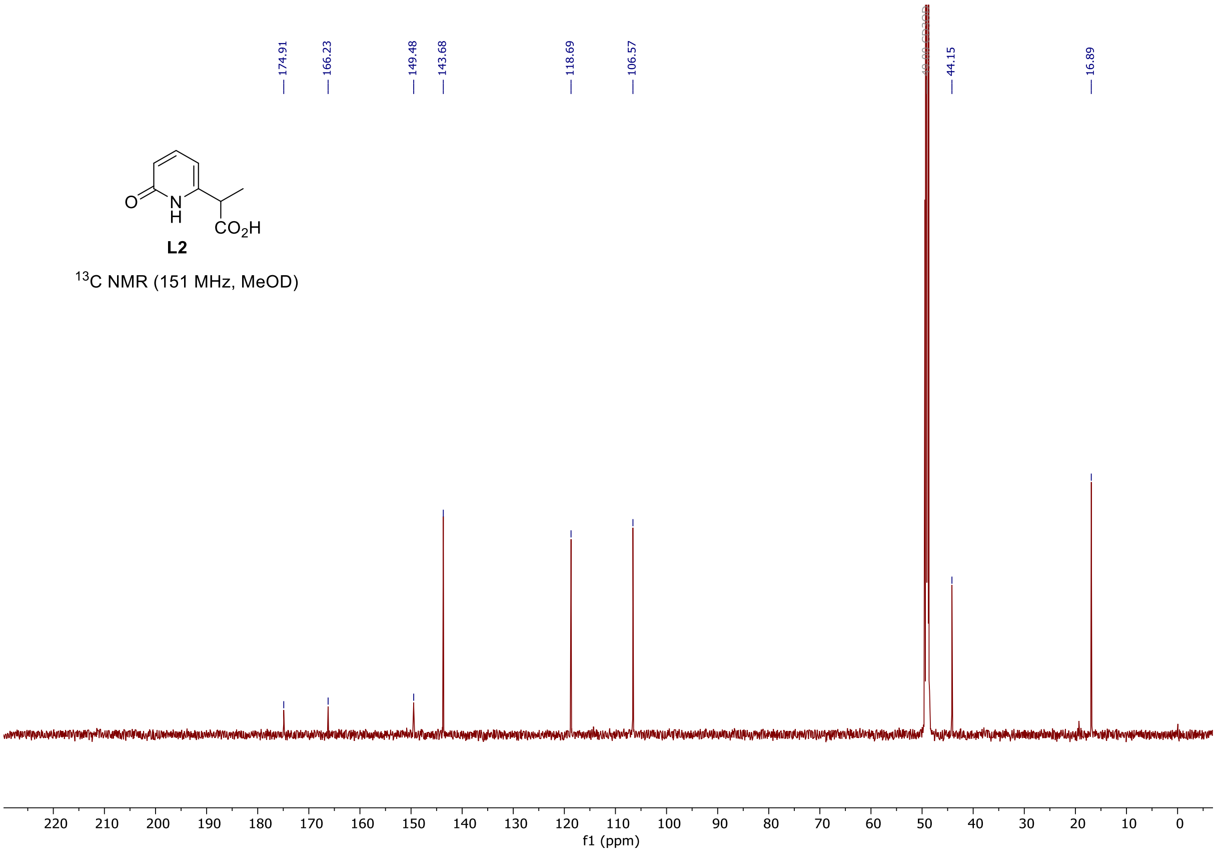

L2

^{13}C NMR (151 MHz, MeOD)

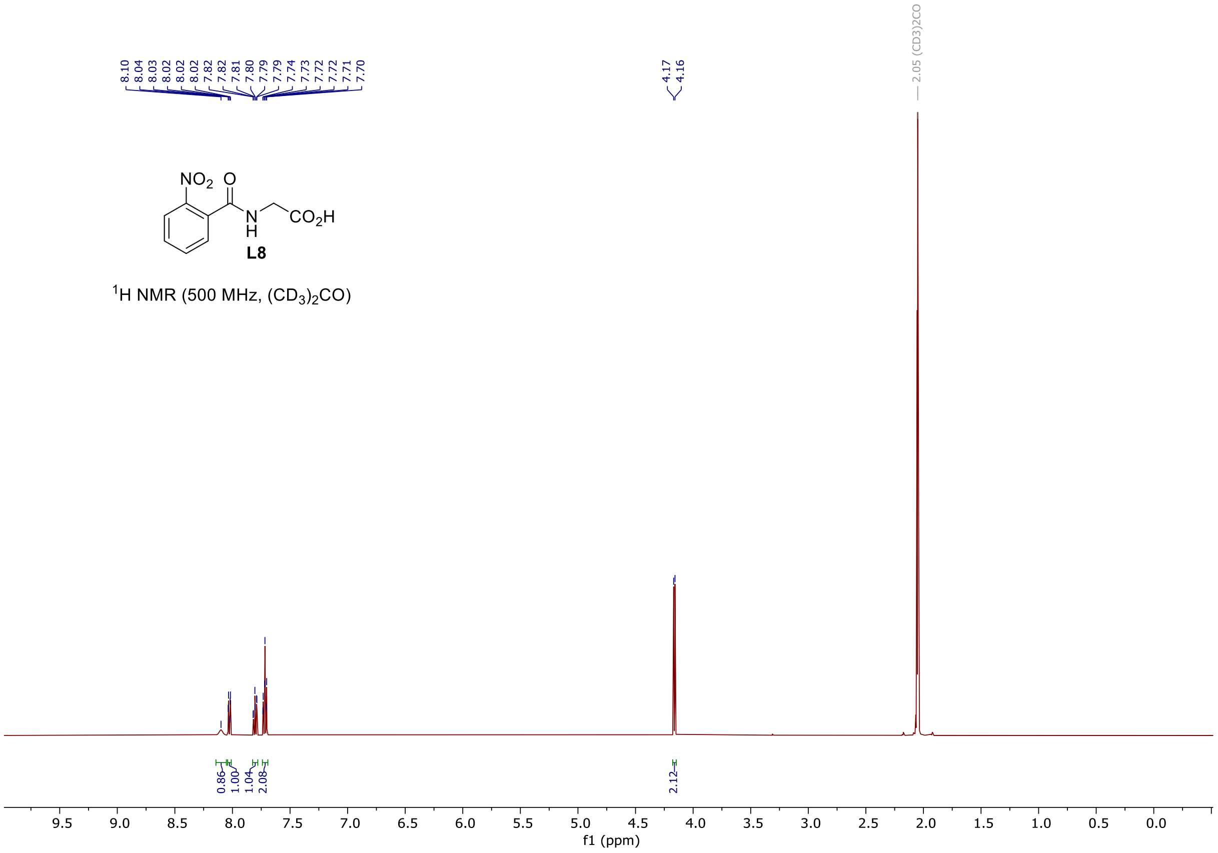
8.10
8.04
8.03
8.02
8.02
8.02
7.82
7.82
7.81
7.80
7.79
7.79
7.74
7.73
7.72
7.72
7.71
7.70
4.17
4.16
2.05 (CD3)2CO
NO_2 O
N
H
CO_2H
L8
^{1}H NMR (500 MHz, $(CD_3)_2CO$)
0.86
1.00
1.04
2.08
2.12
9.5 9.0 8.5 8.0 7.5 7.0 6.5 6.0 5.5 5.0 4.5 4.0 3.5 3.0 2.5 2.0 1.5 1.0 0.5 0.0
f1 (ppm)

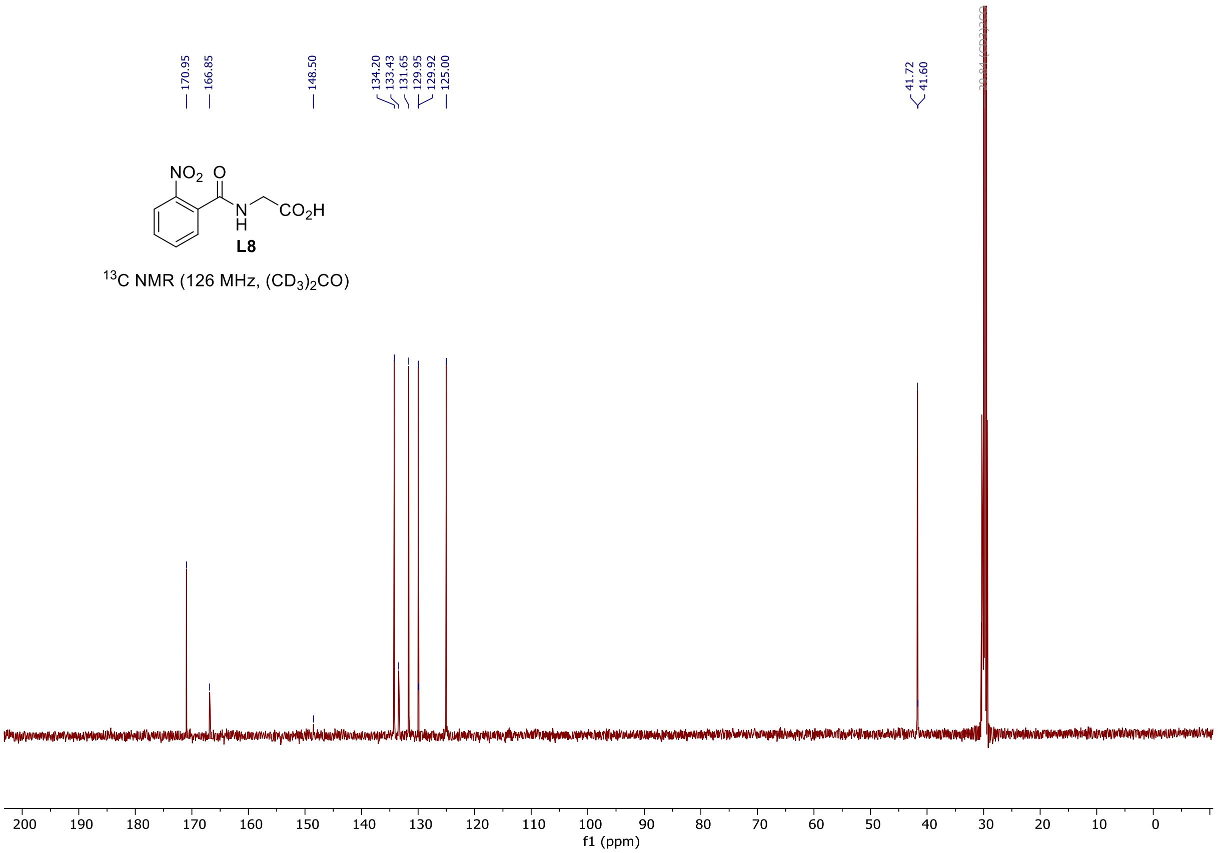
170.95
166.85
148.50
134.20
133.43
131.65
129.95
129.92
125.00
41.72
41.60
NO_2 O
N
H
CO_2H
L8
^{13}C NMR (126 MHz, $(CD_3)_2CO$)
200 190 180 170 160 150 140 130 120 110 100 90 80 70 60 50 40 30 20 10 0
f1 (ppm)

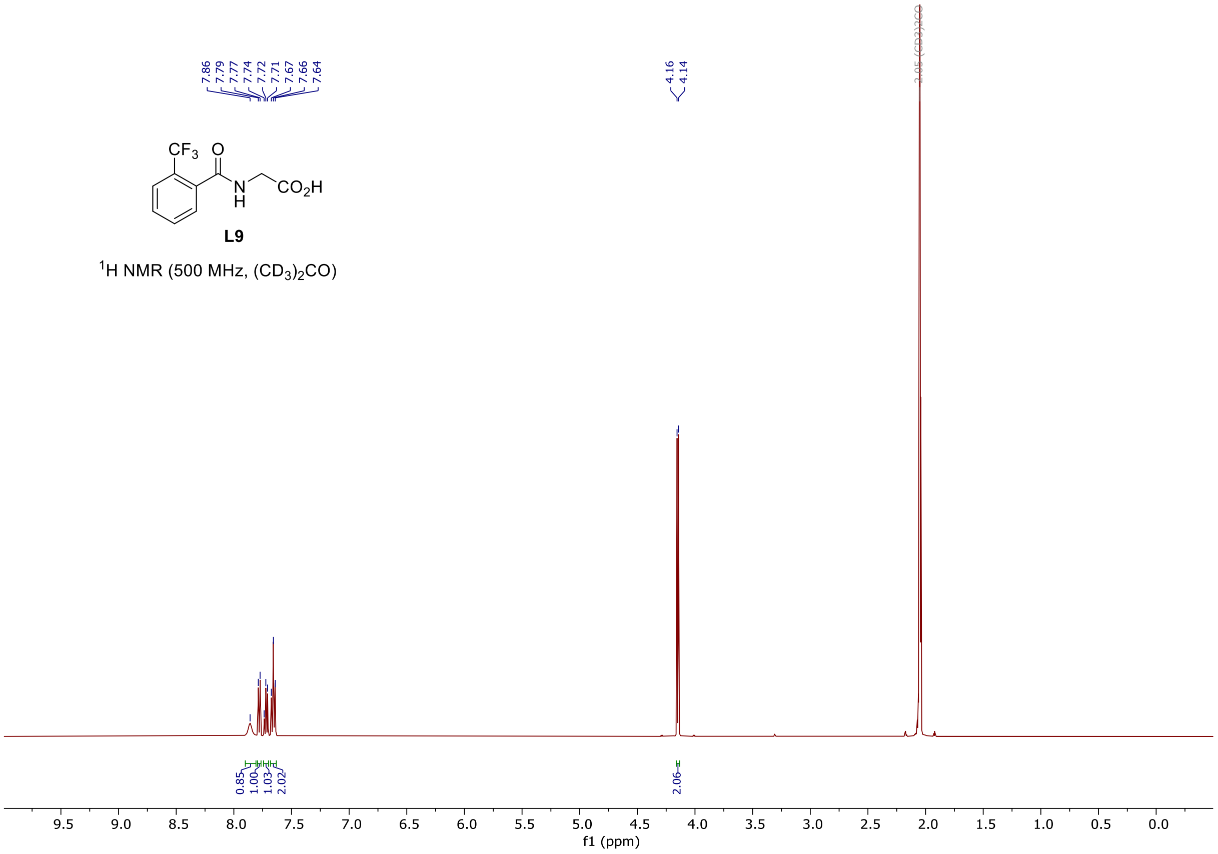

L9

^{1}H NMR (500 MHz, $(CD_3)_2CO$)

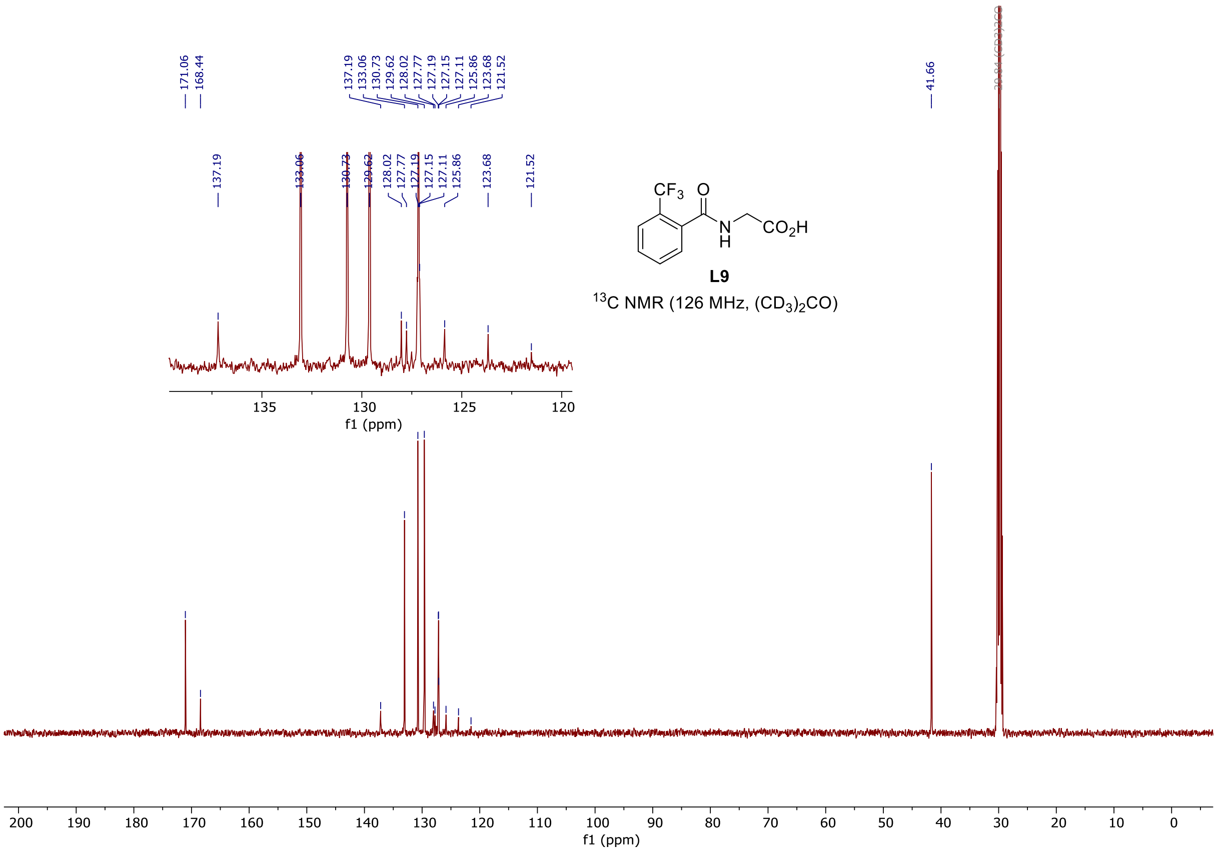

L9

^{13}C NMR (126 MHz, $(CD_3)_2CO$)

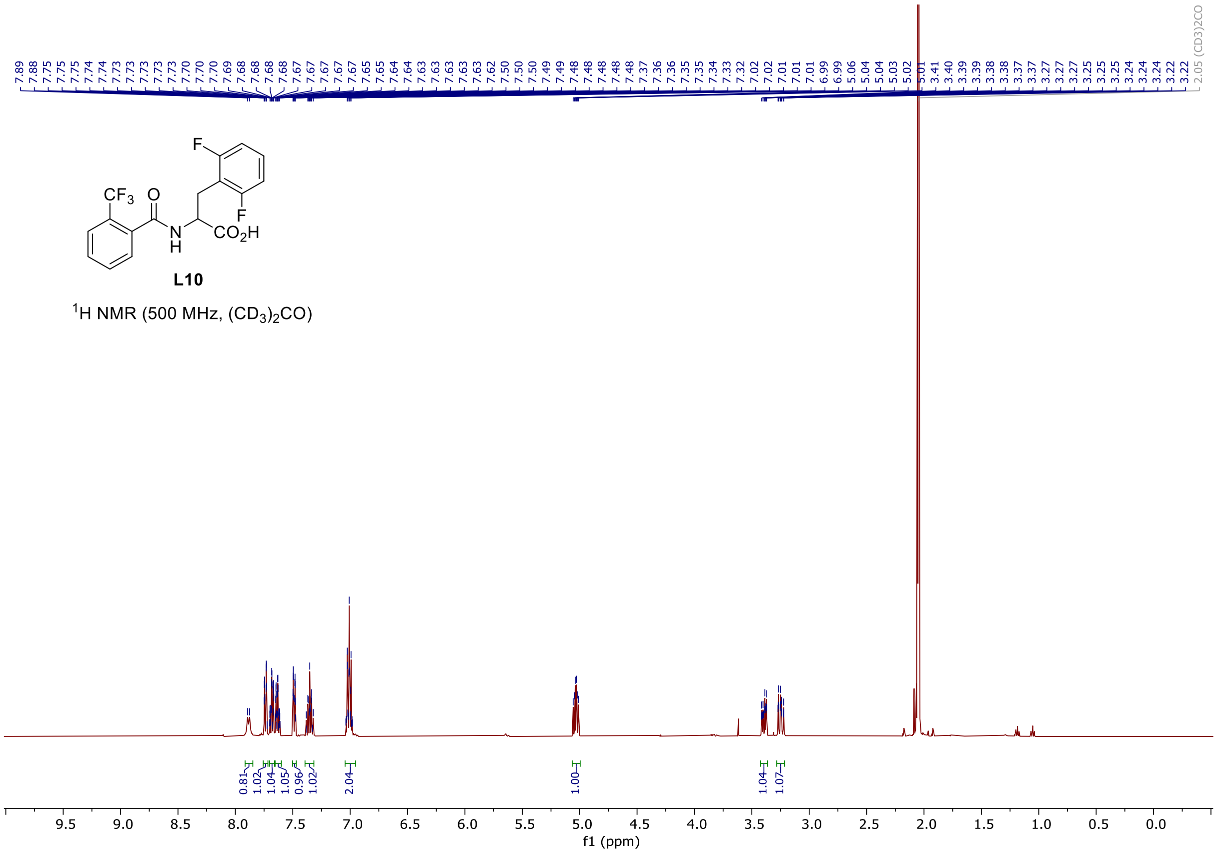
L10
^{1}H NMR (500 MHz, $(CD_3)_2CO$)
f1 (ppm)

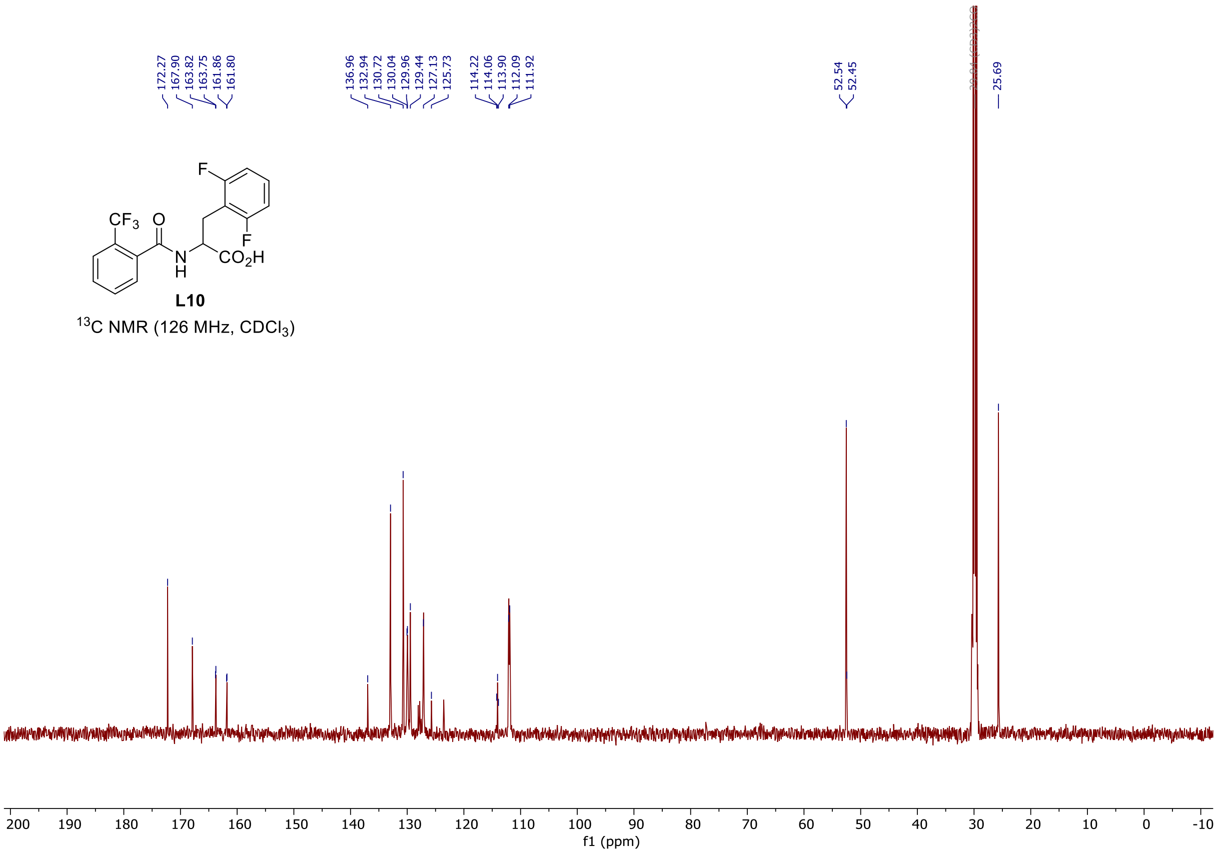
L10
^{13}C NMR (126 MHz, $CDCl_3$)
f1 (ppm)

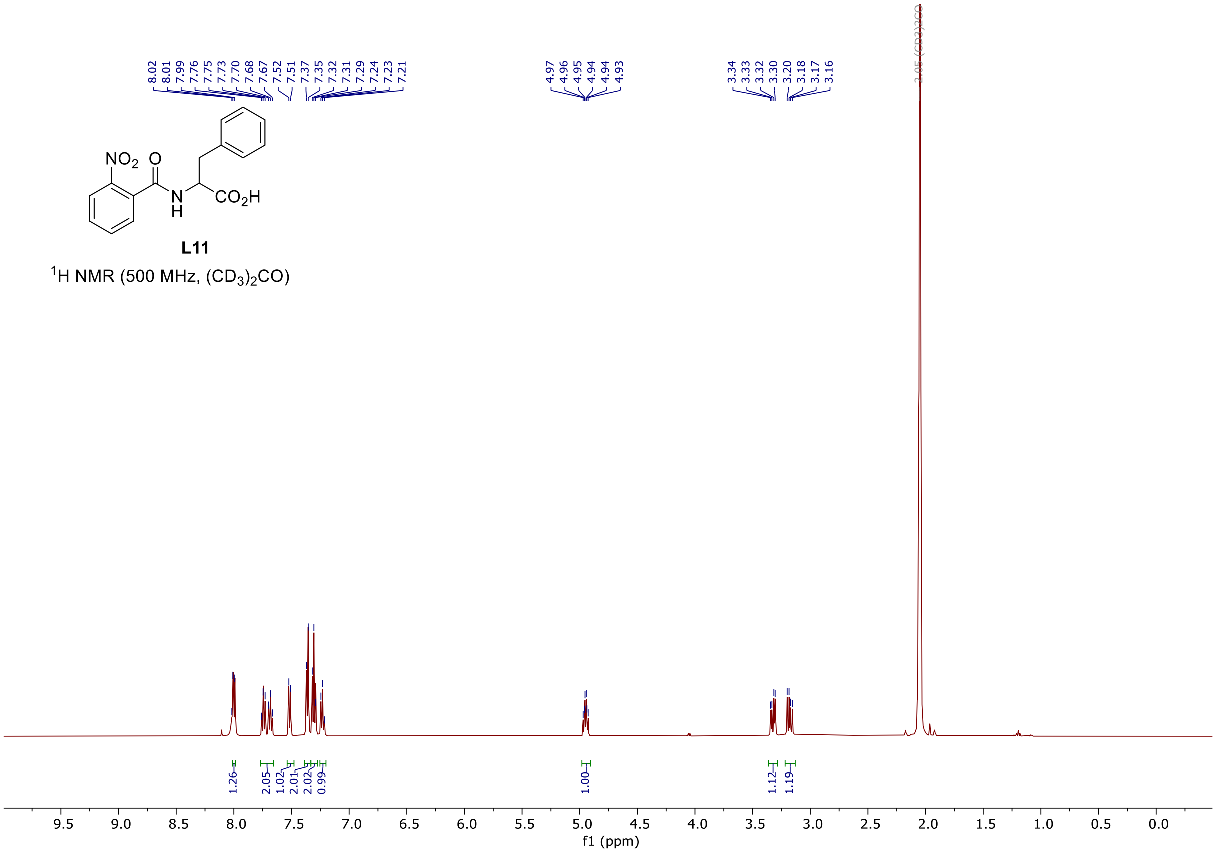

L11

^{1}H NMR (500 MHz, $(CD_3)_2CO$)

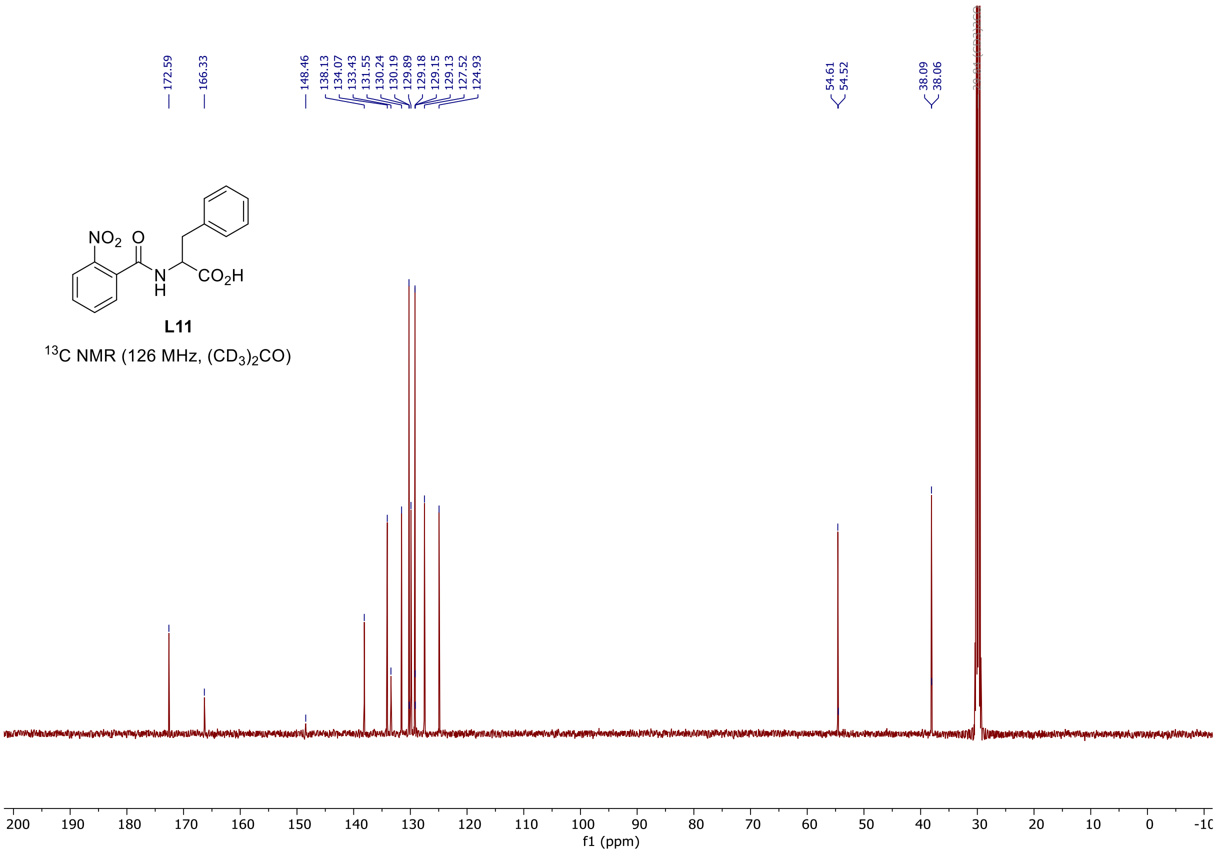

L11

^{13}C NMR (126 MHz, $(CD_3)_2CO$)

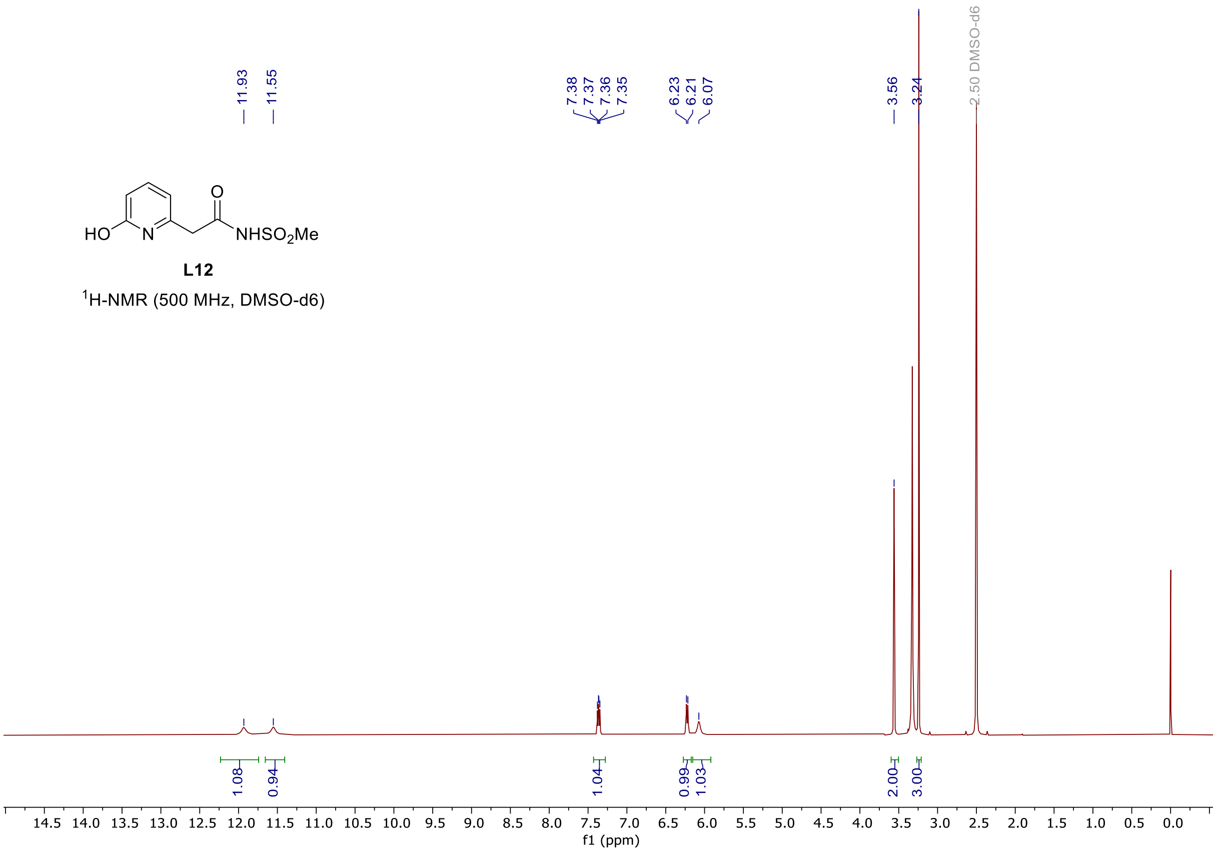
11.93
11.55
7.38
7.37
7.36
7.35
6.23
6.21
6.07
3.56
3.24
2.50 DMSO-d6
HO
N
O
NHSO2Me
L12
1H-NMR (500 MHz, DMSO-d6)
1.08
0.94
1.04
0.99
1.03
2.00
3.00
14.5 14.0 13.5 13.0 12.5 12.0 11.5 11.0 10.5 10.0 9.5 9.0 8.5 8.0 7.5 7.0 6.5 6.0 5.5 5.0 4.5 4.0 3.5 3.0 2.5 2.0 1.5 1.0 0.5 0.0
f1 (ppm)

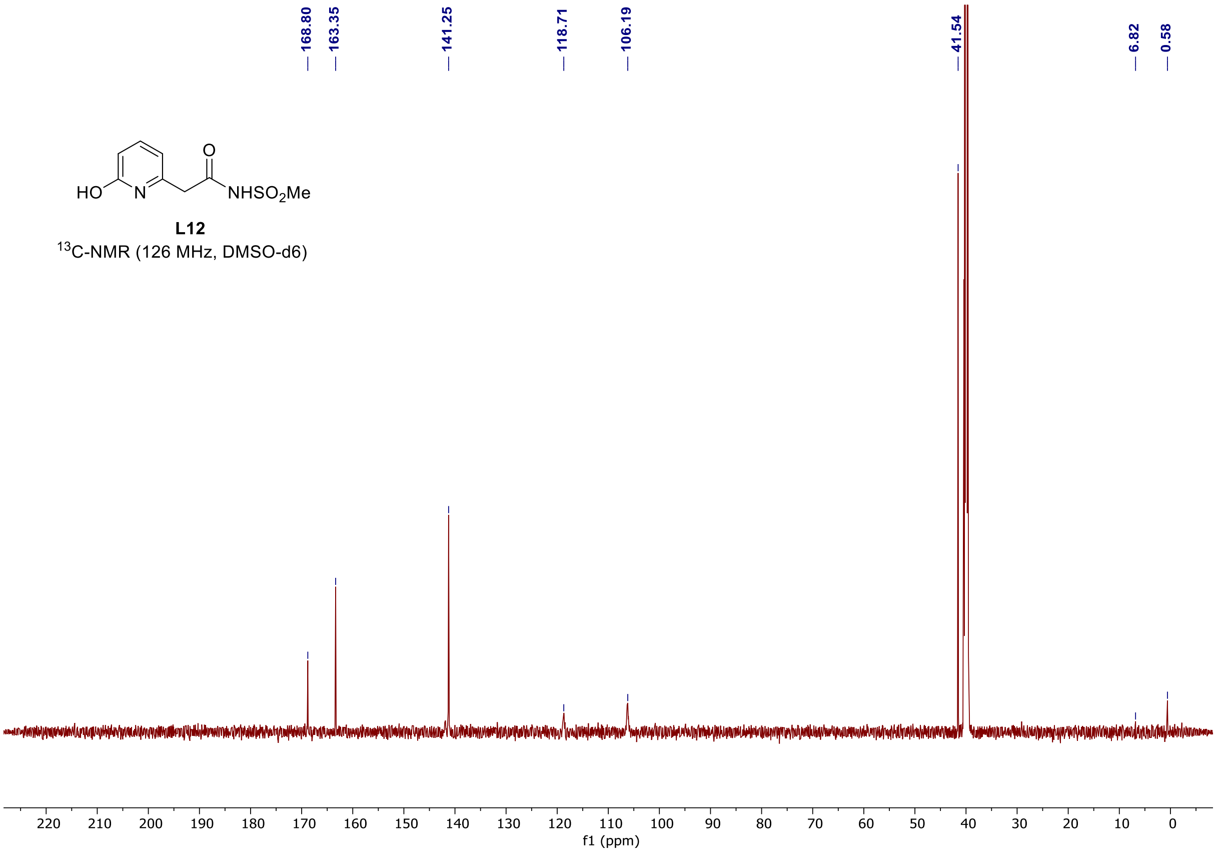
168.80
163.35
141.25
118.71
106.19
41.54
6.82
0.58
HO
N
O
NHSO2Me
L12
13C-NMR (126 MHz, DMSO-d6)
220 210 200 190 180 170 160 150 140 130 120 110 100 90 80 70 60 50 40 30 20 10 0
f1 (ppm)

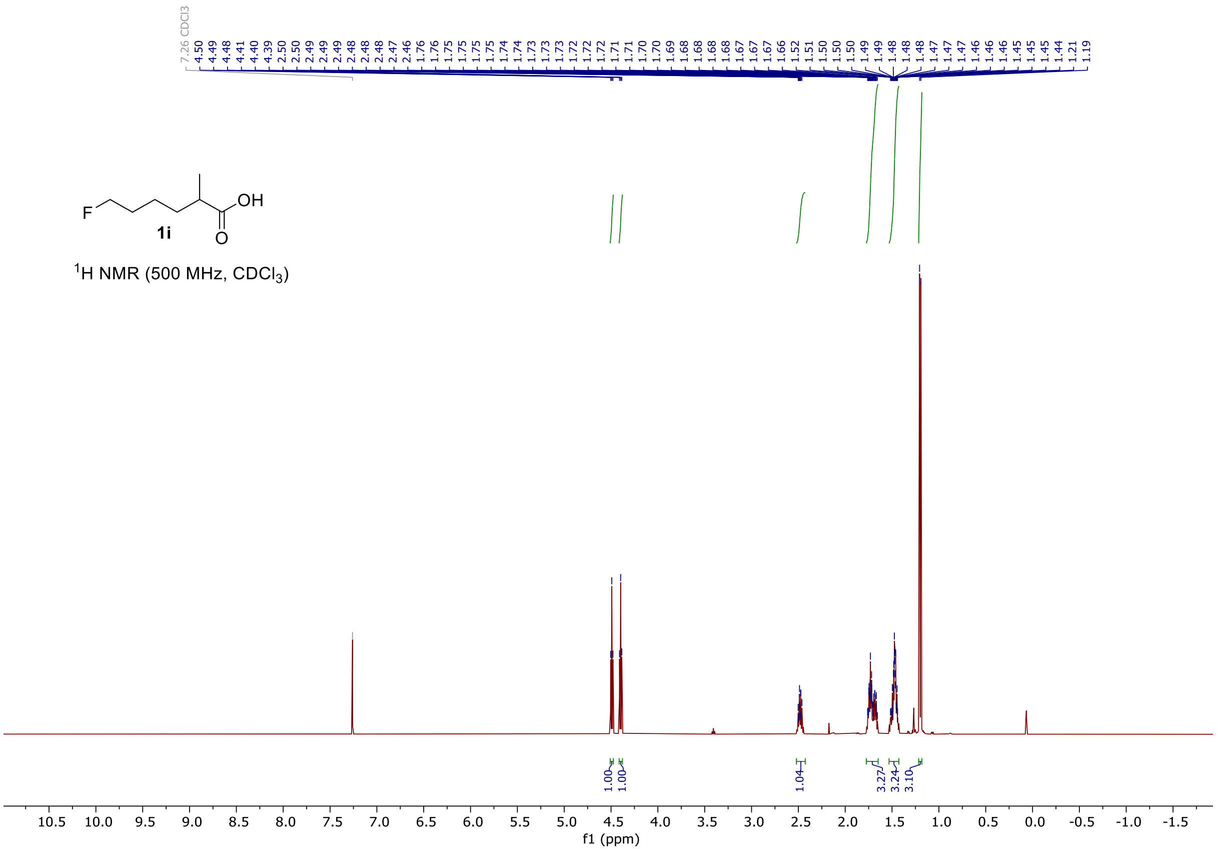
7.26 CDCl3
4.50 4.49 4.48 4.41 4.40 4.39 2.50 2.50 2.49 2.49 2.49 2.48 2.48 2.48 2.47 2.46 1.76 1.76 1.75 1.75 1.75 1.74 1.74 1.73 1.73 1.73 1.72 1.72 1.72 1.71 1.71 1.70 1.69 1.68 1.68 1.68 1.67 1.67 1.67 1.66 1.52 1.51 1.50 1.50 1.49 1.49 1.48 1.48 1.48 1.47 1.47 1.46 1.46 1.46 1.45 1.45 1.45 1.44 1.21 1.19
F
OH
O
1i
^{1}H NMR (500 MHz, CDCl$_3$)
1.00
1.00
1.04
3.27
3.24
3.10
10.5 10.0 9.5 9.0 8.5 8.0 7.5 7.0 6.5 6.0 5.5 5.0 4.5 4.0 3.5 3.0 2.5 2.0 1.5 1.0 0.5 0.0 -0.5 -1.0 -1.5
f1 (ppm)

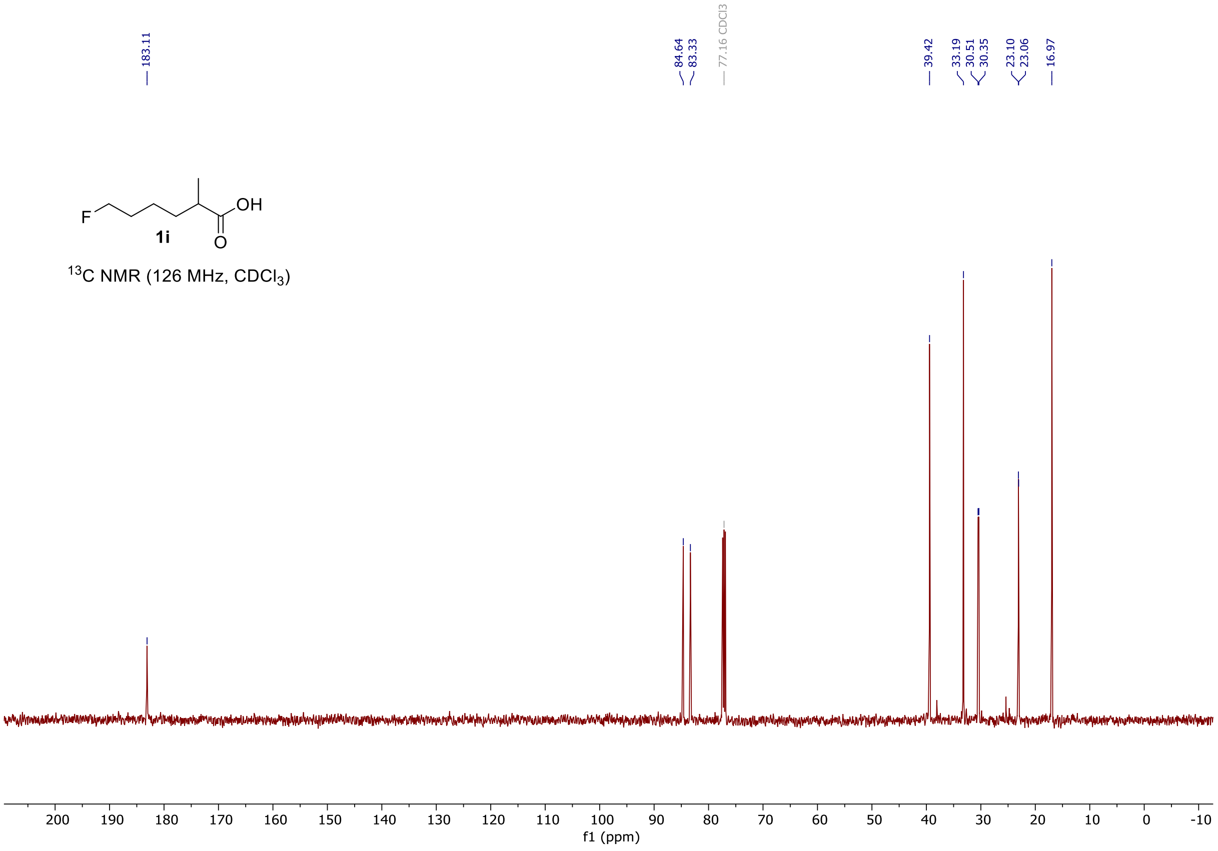
183.11
84.64
83.33
77.16 CDCl3
39.42
33.19
30.51
30.35
23.10
23.06
16.97
F
OH
O
1i
^{13}C NMR (126 MHz, CDCl$_3$)
200 190 180 170 160 150 140 130 120 110 100 90 80 70 60 50 40 30 20 10 0 -10
f1 (ppm)

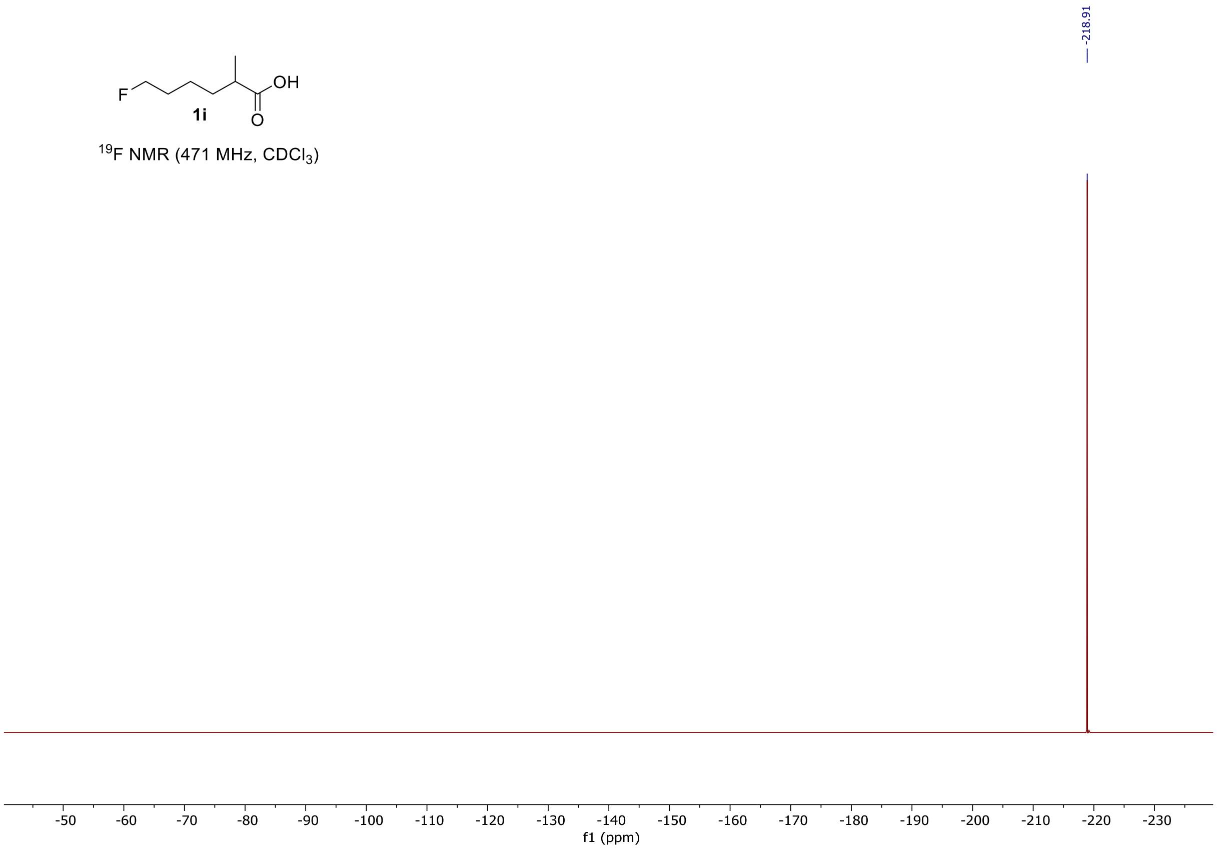
OH
F
1i
O
^{19}F NMR (471 MHz, CDCl$_3$)
-218.91
-50
-60
-70
-80
-90
-100
-110
-120
-130
-140
-150
-160
-170
-180
-190
-200
-210
-220
-230
f1 (ppm)

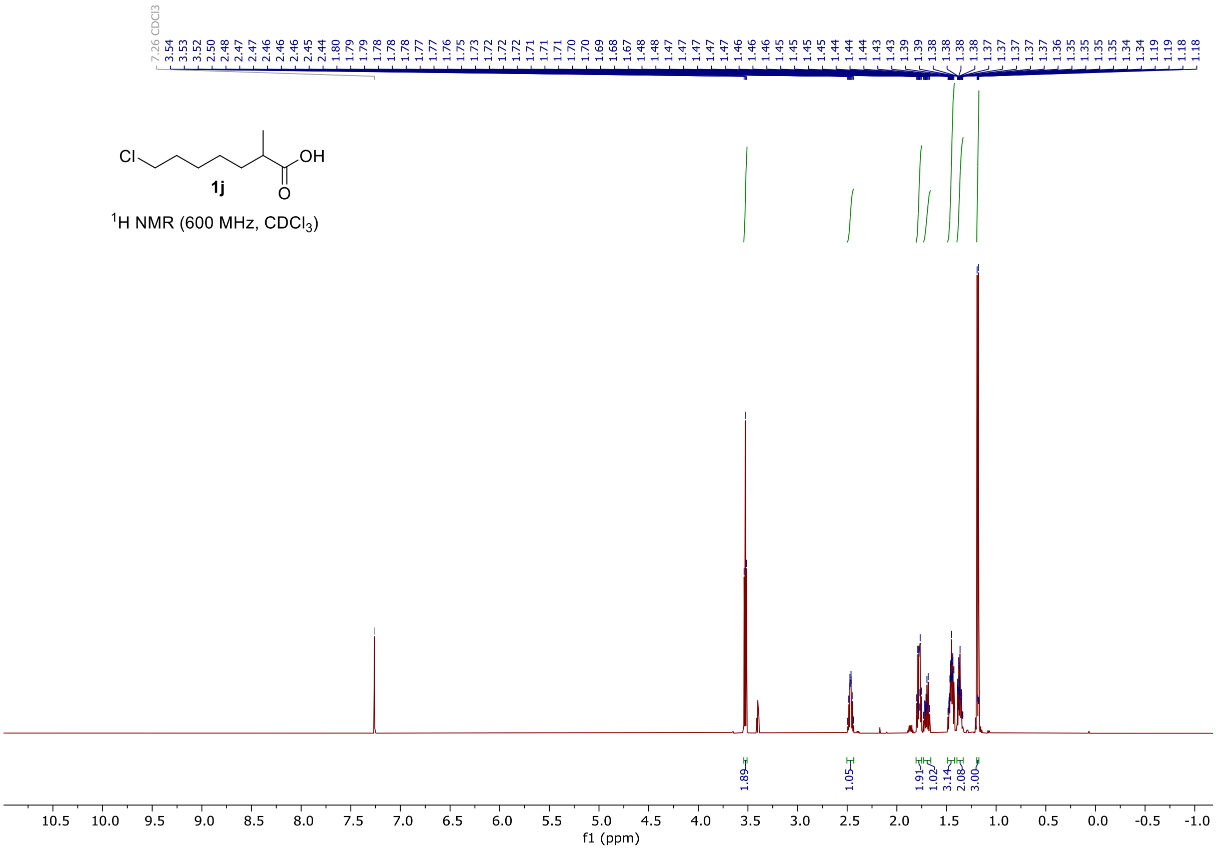

1j

^{1}H NMR (600 MHz, $CDCl_3$)

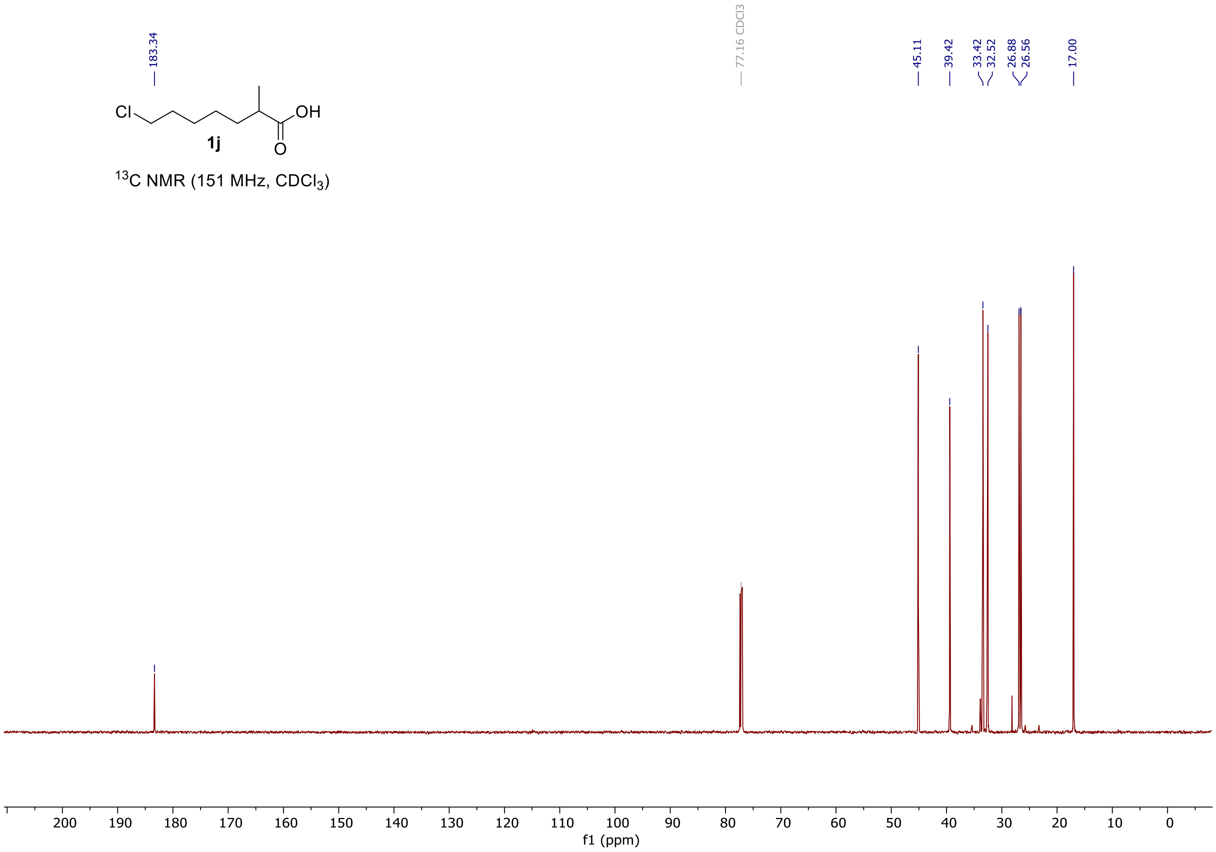

1j

^{13}C NMR (151 MHz, $CDCl_3$)

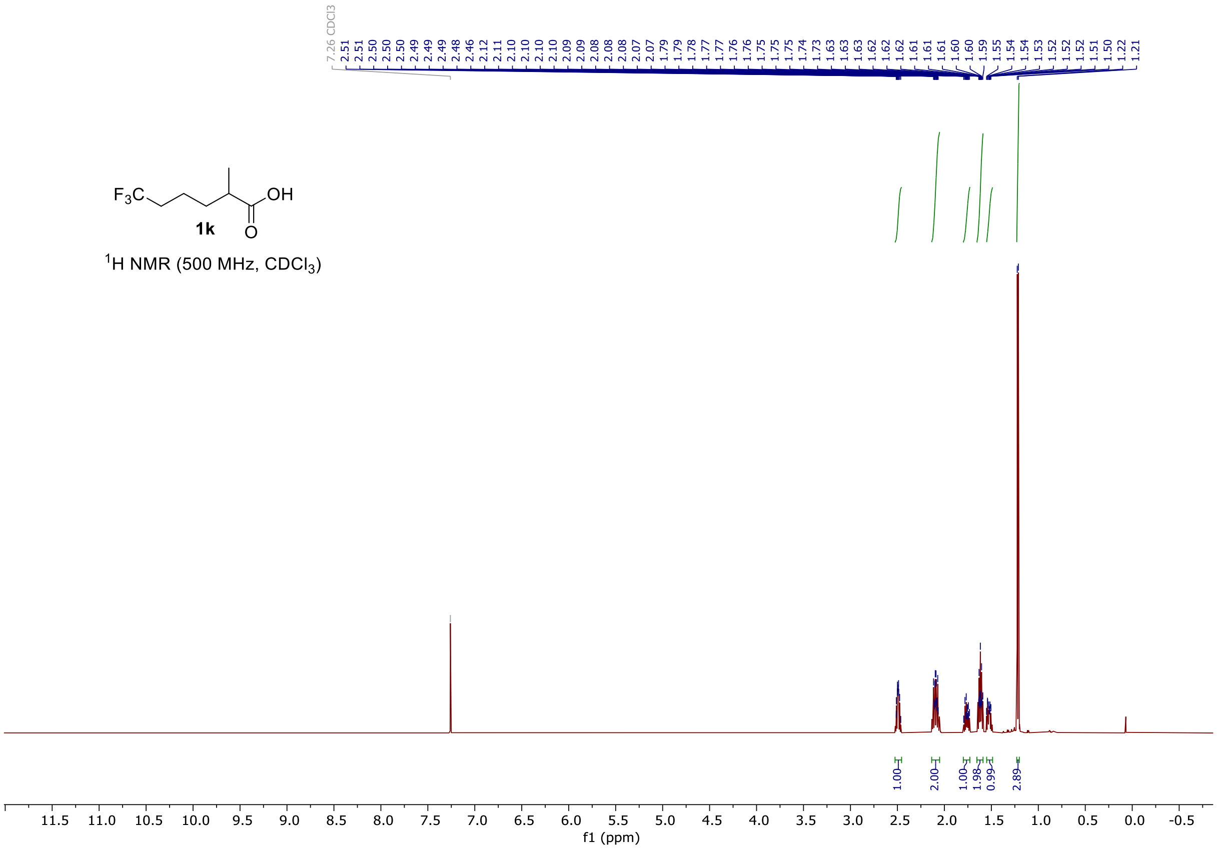
F3C OH
1k O
1H NMR (500 MHz, CDCl3)
7.26 CDCl3
2.51 2.51 2.50 2.50 2.50 2.49 2.49 2.48 2.46 2.12 2.11 2.10 2.10 2.10 2.09 2.09 2.08 2.08 2.08 2.07 2.07 1.79 1.79 1.78 1.77 1.77 1.76 1.76 1.75 1.75 1.75 1.74 1.73 1.63 1.63 1.63 1.62 1.62 1.61 1.61 1.60 1.59 1.55 1.54 1.54 1.53 1.52 1.52 1.52 1.51 1.50 1.22 1.21
1.00 2.00 1.00 1.98 0.99 2.89
11.5 11.0 10.5 10.0 9.5 9.0 8.5 8.0 7.5 7.0 6.5 6.0 5.5 5.0 4.5 4.0 3.5 3.0 2.5 2.0 1.5 1.0 0.5 0.0 -0.5
f1 (ppm)

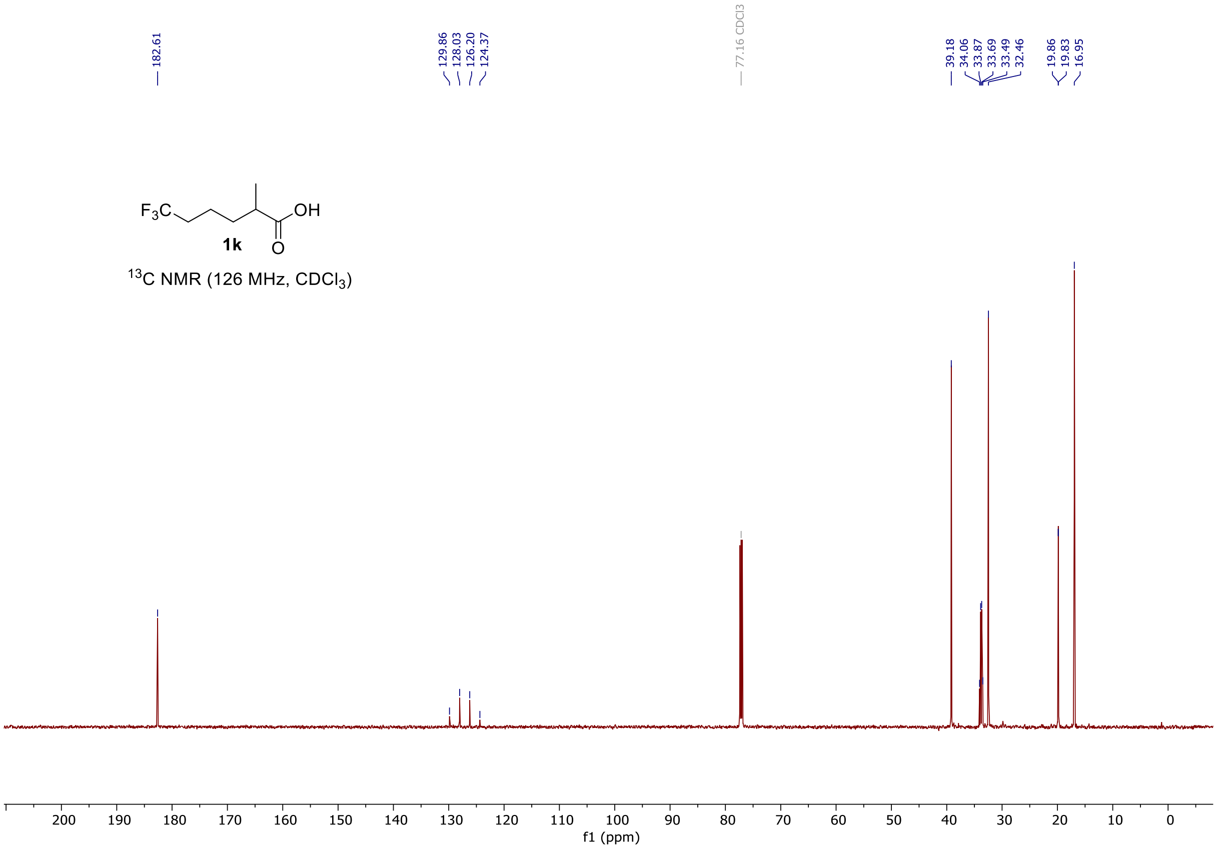
182.61
129.86 128.03 126.20 124.37
77.16 CDCl3
39.18 34.06 33.87 33.69 33.49 32.46
19.86 19.83 16.95
F3C OH
1k O
13C NMR (126 MHz, CDCl3)
200 190 180 170 160 150 140 130 120 110 100 90 80 70 60 50 40 30 20 10 0
f1 (ppm)

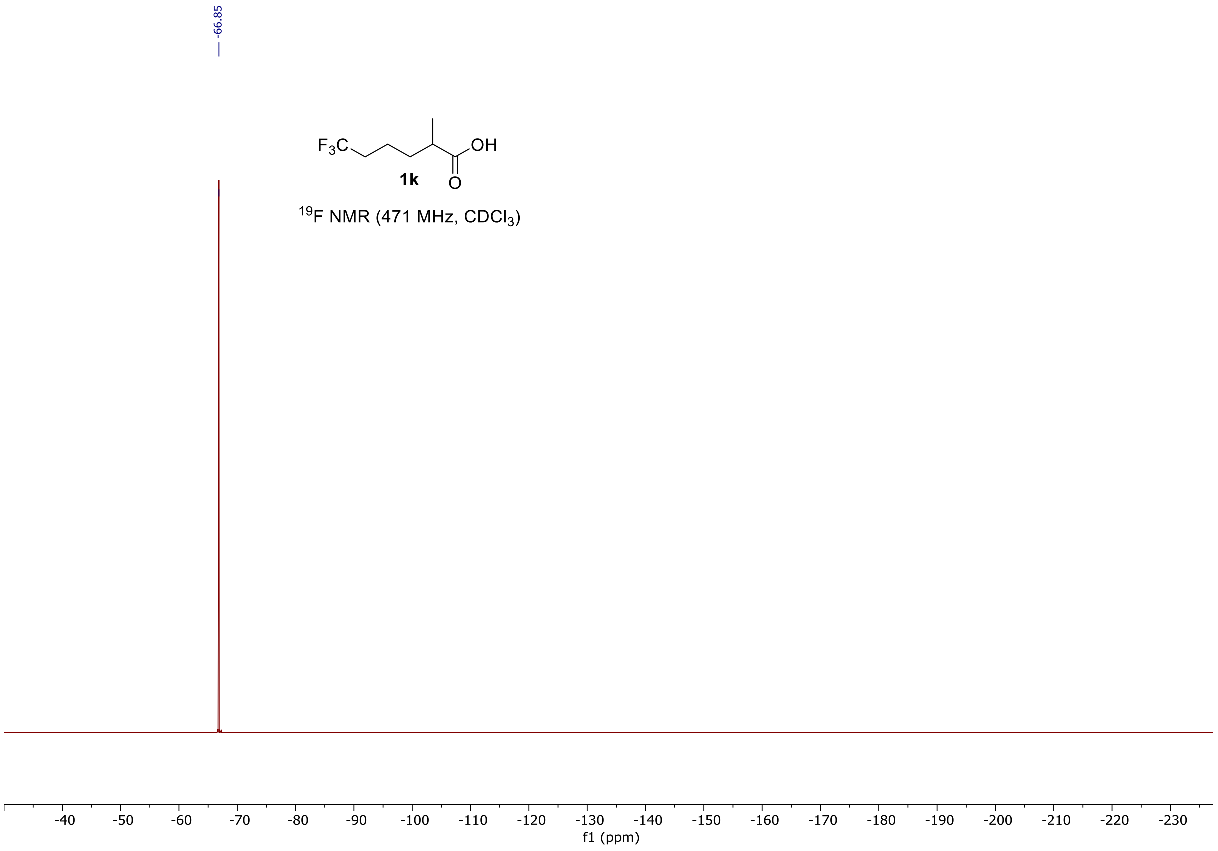
-66.85
F3C
OH
O
1k
19F NMR (471 MHz, CDCl3)
-40 -50 -60 -70 -80 -90 -100 -110 -120 -130 -140 -150 -160 -170 -180 -190 -200 -210 -220 -230
f1 (ppm)

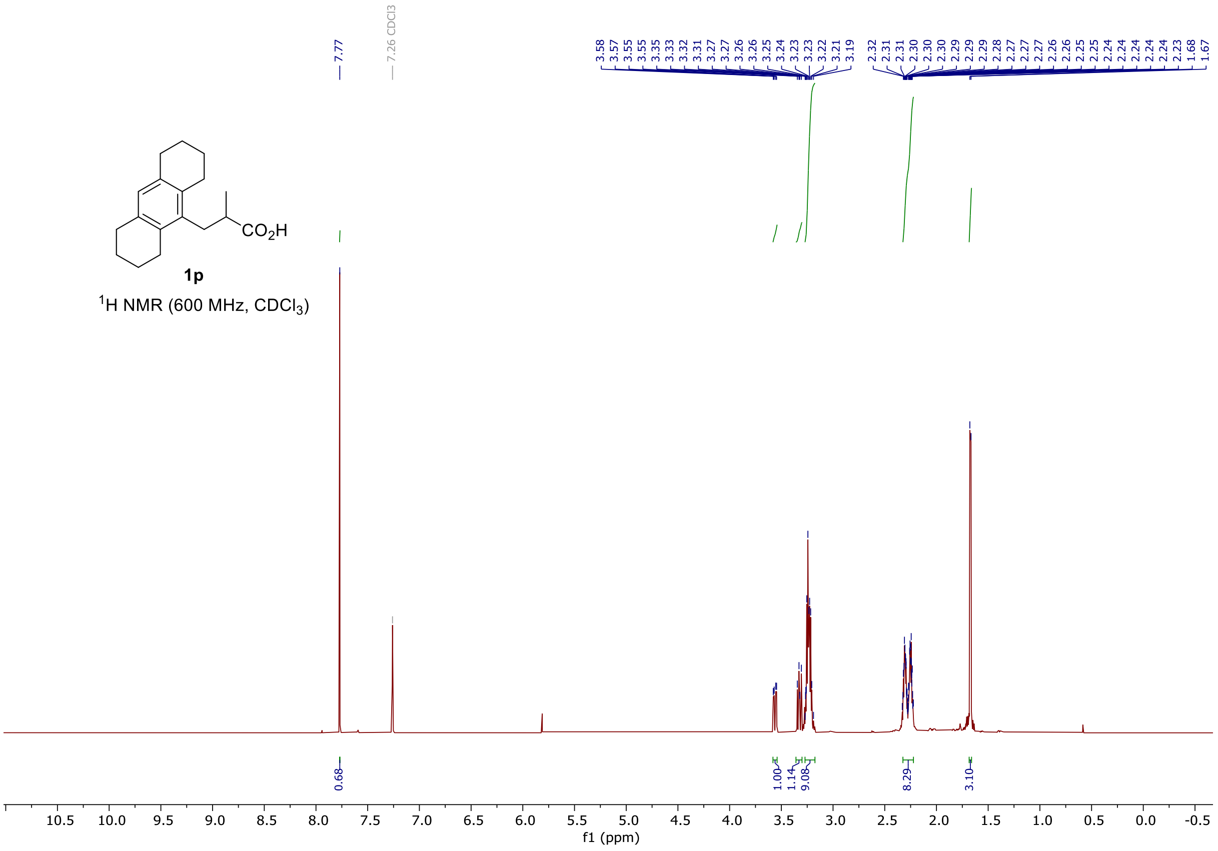

7.77
7.26 CDCl3
3.58 3.57 3.55 3.55 3.35 3.33 3.32 3.31 3.27 3.27 3.26 3.26 3.25 3.24 3.23 3.23 3.22 3.21 3.19
2.32 2.31 2.31 2.30 2.30 2.30 2.29 2.29 2.29 2.28 2.27 2.27 2.27 2.26 2.26 2.25 2.25 2.24 2.24 2.24 2.24 2.24 2.23 1.68 1.67
CO2H
1p
1H NMR (600 MHz, CDCl3)
0.68
1.00
1.14
9.08
8.29
3.10
10.5 10.0 9.5 9.0 8.5 8.0 7.5 7.0 6.5 6.0 5.5 5.0 4.5 4.0 3.5 3.0 2.5 2.0 1.5 1.0 0.5 0.0 -0.5
f1 (ppm)

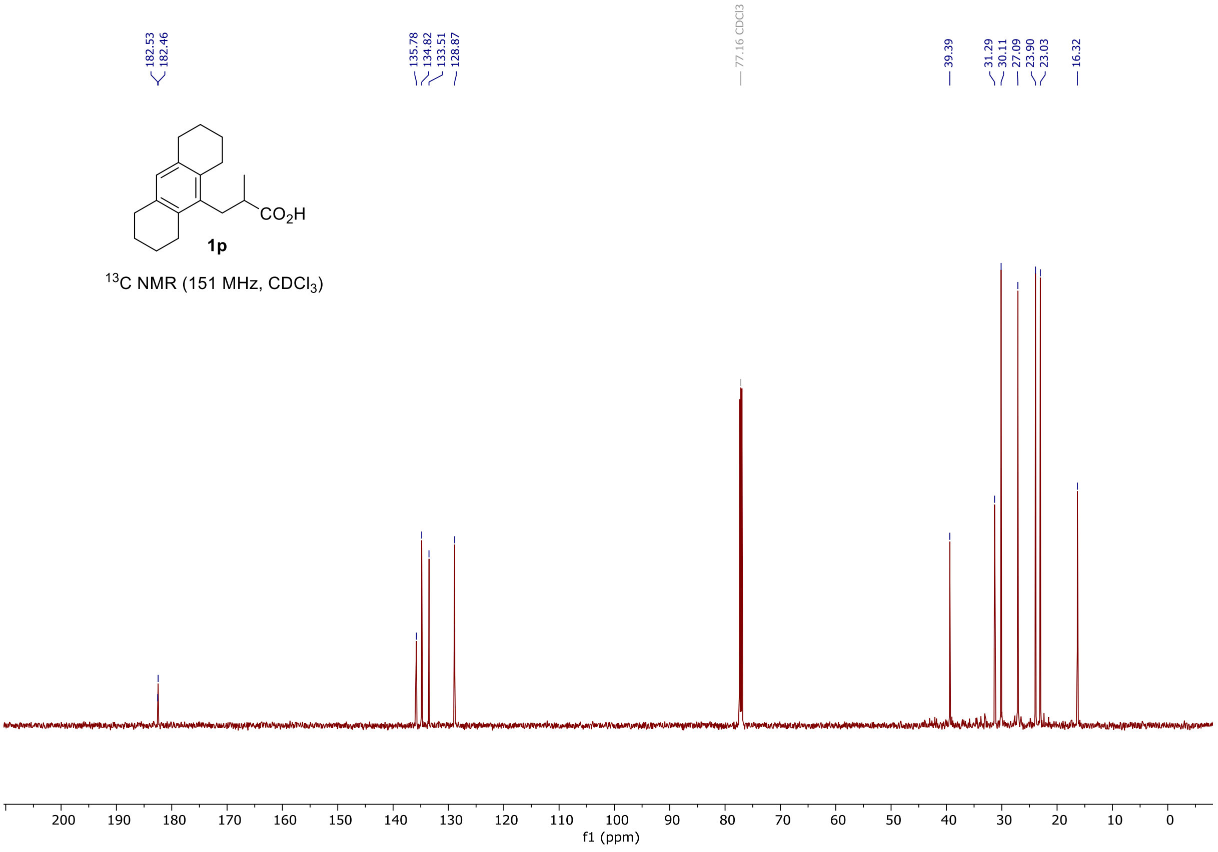

182.53
182.46
135.78
134.82
133.51
128.87
77.16 CDCl3
39.39
31.29
30.11
27.09
23.90
23.03
16.32
CO2H
1p
13C NMR (151 MHz, CDCl3)
200 190 180 170 160 150 140 130 120 110 100 90 80 70 60 50 40 30 20 10 0
f1 (ppm)

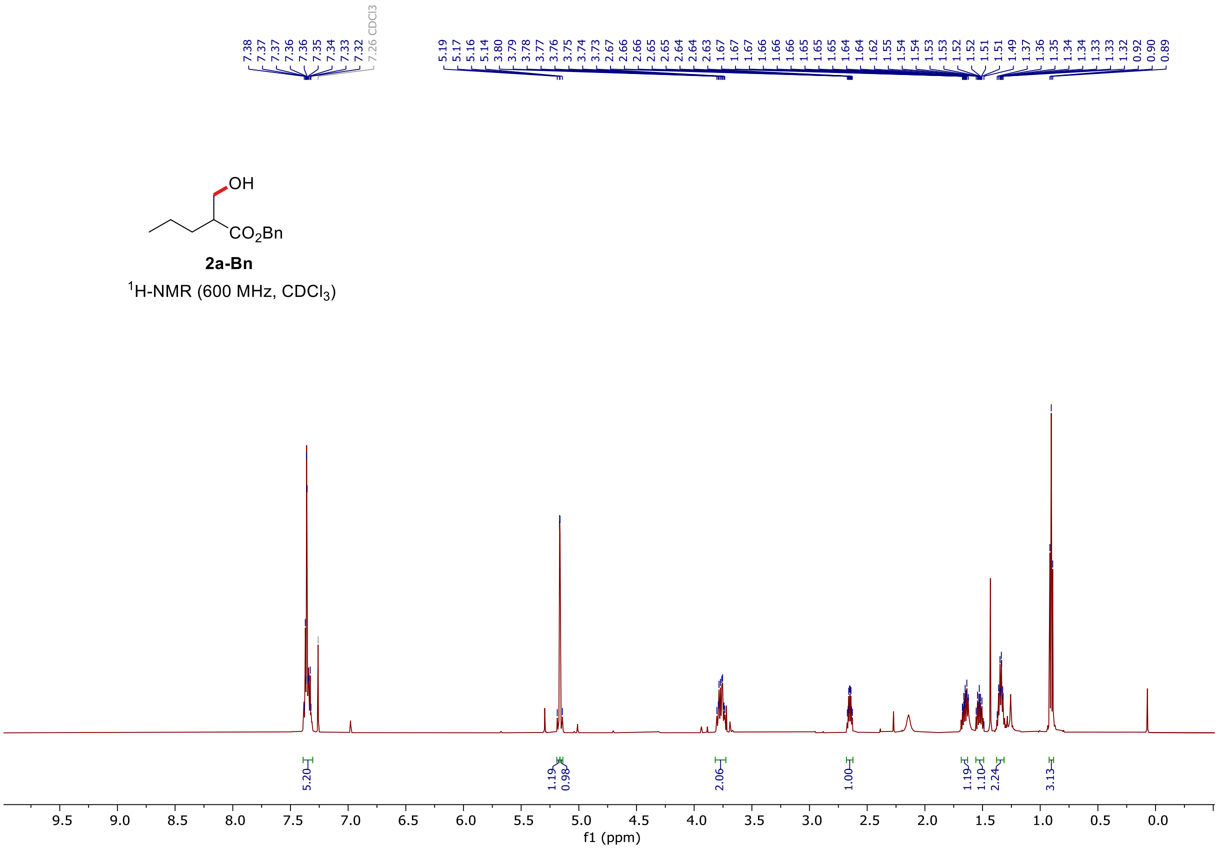

7.38
7.37
7.37
7.36
7.36
7.35
7.34
7.33
7.32
7.26 CDCl3
5.19
5.17
5.16
5.14
3.80
3.79
3.78
3.77
3.76
3.75
3.74
3.73
2.67
2.66
2.66
2.65
2.65
2.64
2.63
1.67
1.67
1.67
1.66
1.66
1.65
1.65
1.65
1.64
1.64
1.62
1.55
1.54
1.54
1.53
1.53
1.52
1.52
1.51
1.51
1.49
1.37
1.36
1.35
1.34
1.34
1.33
1.33
1.32
0.92
0.90
0.89
OH
CO2Bn
2a-Bn
1H-NMR (600 MHz, CDCl3)
5.20
1.19
0.98
2.06
1.00
1.19
1.10
2.24
3.13
f1 (ppm)

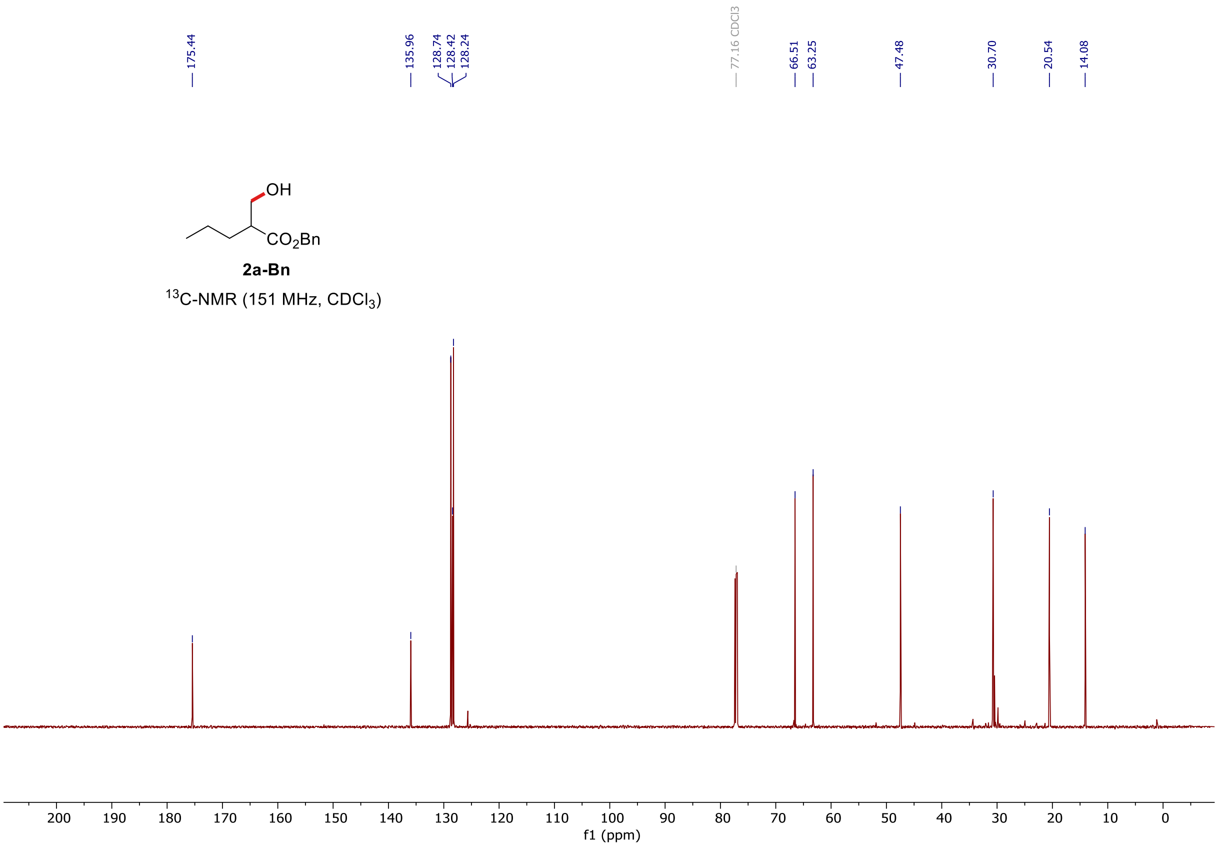

175.44
135.96
128.74
128.42
128.24
77.16 CDCl3
66.51
63.25
47.48
30.70
20.54
14.08
OH
CO2Bn
2a-Bn
13C-NMR (151 MHz, CDCl3)
f1 (ppm)

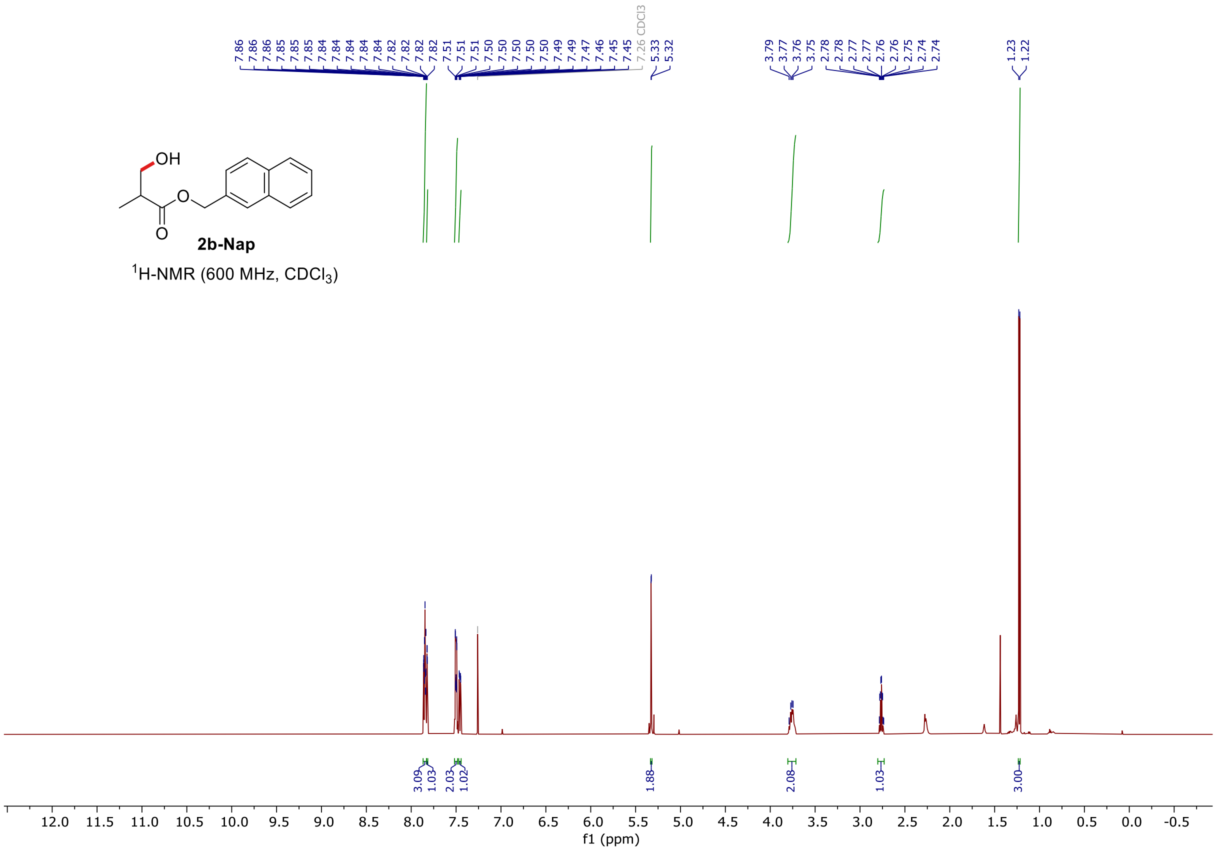

2b-Nap
^{1}H-NMR (600 MHz, CDCl$_3$)
f1 (ppm)

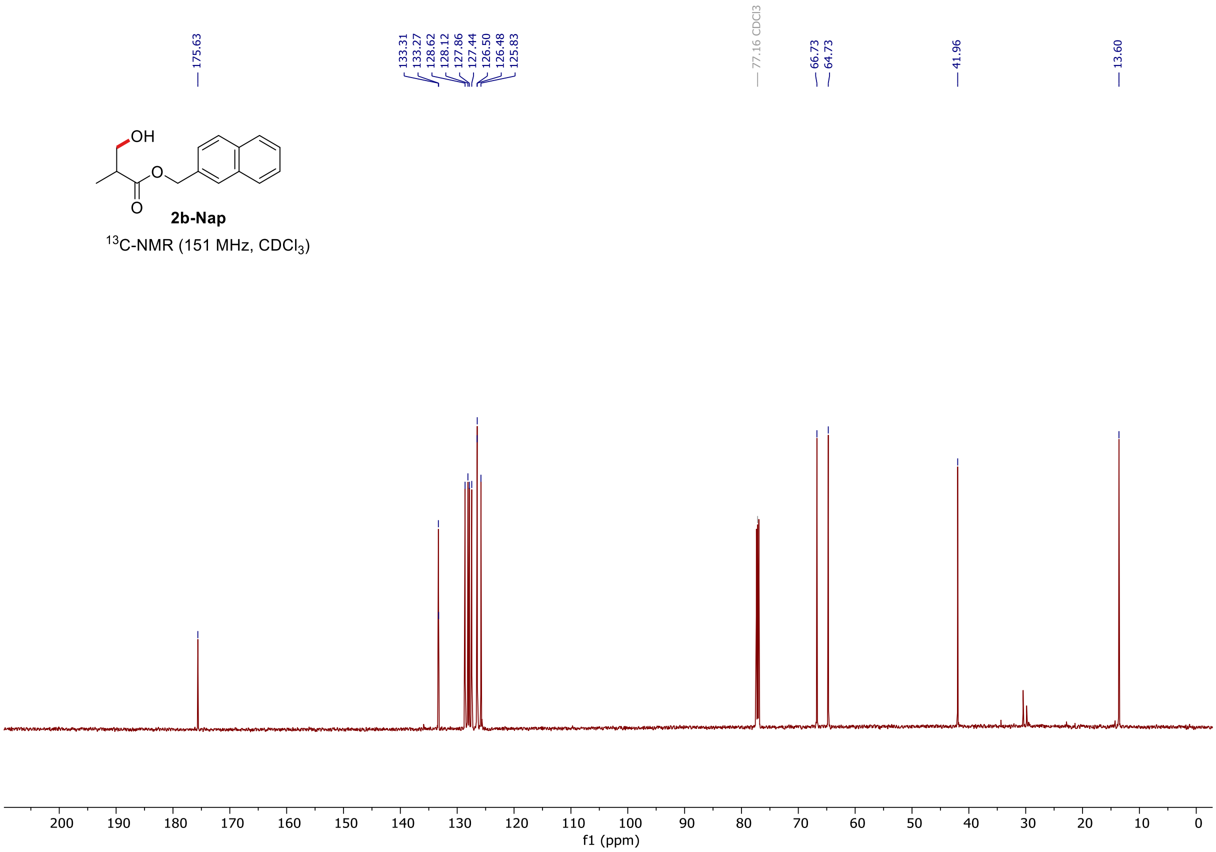

2b-Nap
^{13}C-NMR (151 MHz, CDCl$_3$)
f1 (ppm)

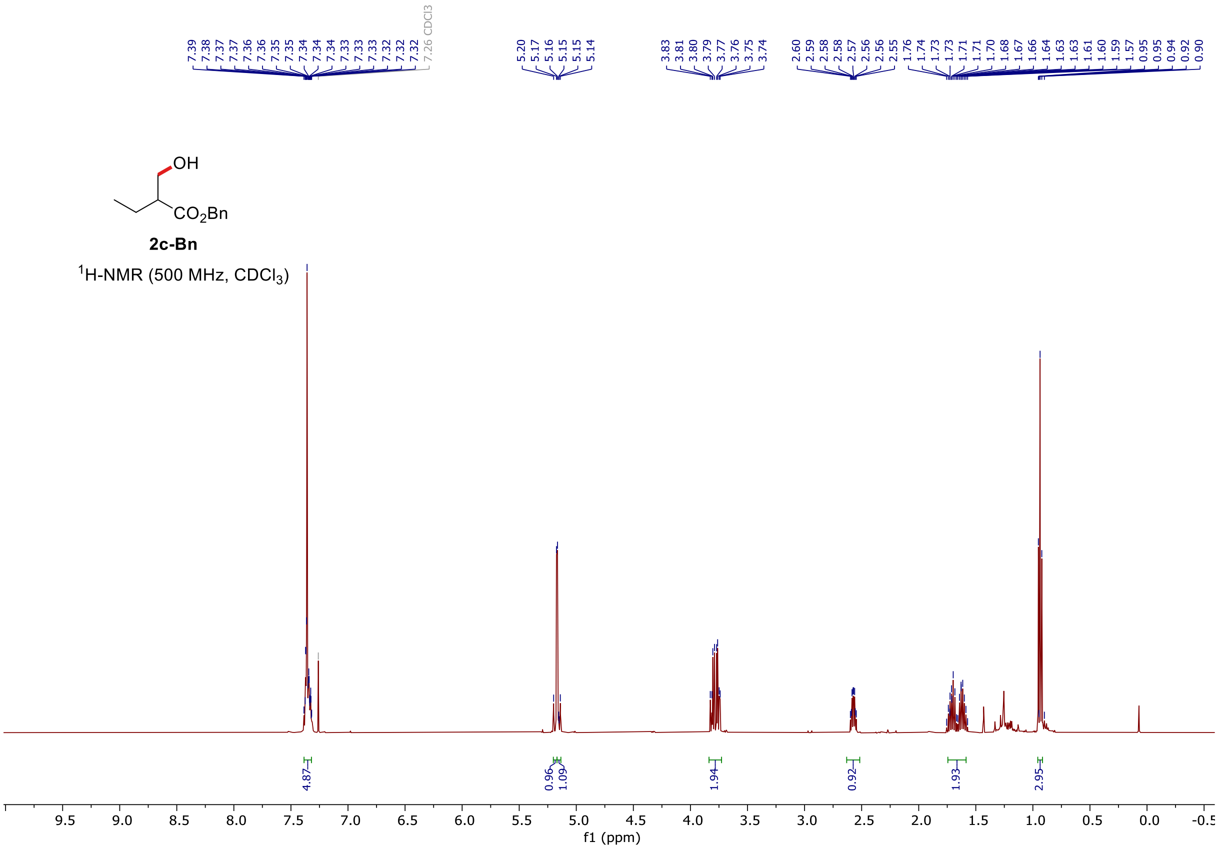
7.39 7.38 7.37 7.37 7.36 7.36 7.35 7.35 7.34 7.34 7.34 7.33 7.33 7.33 7.32 7.32 7.32 7.26 CDCl3
5.20 5.17 5.16 5.15 5.15 5.14
3.83 3.81 3.80 3.79 3.77 3.76 3.75 3.74
2.60 2.59 2.58 2.58 2.57 2.56 2.56 2.55
1.76 1.74 1.73 1.73 1.71 1.71 1.70 1.68 1.67 1.66 1.64 1.63 1.63 1.61 1.60 1.59 1.57 0.95 0.95 0.94 0.92 0.90
OH
CO2Bn
2c-Bn
1H-NMR (500 MHz, CDCl3)
4.87
0.96
1.09
1.94
0.92
1.93
2.95
f1 (ppm)

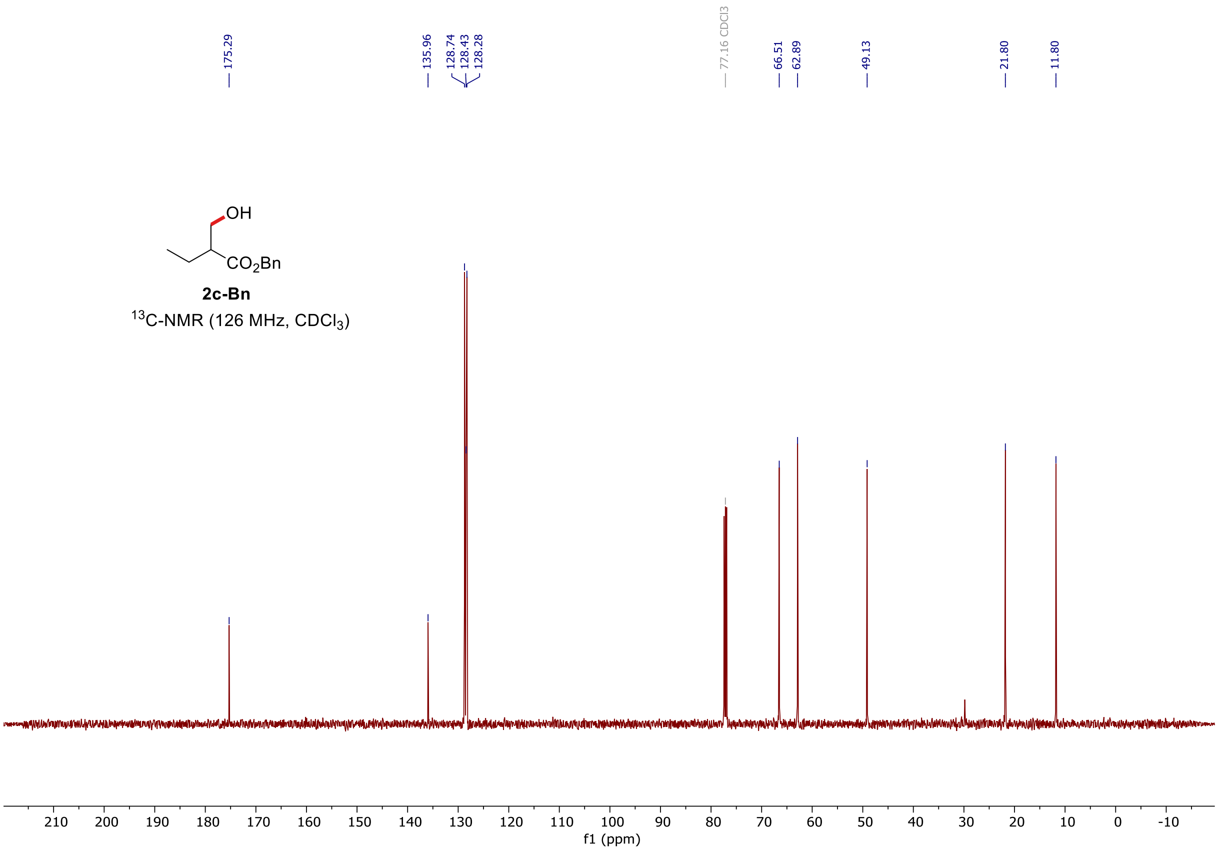
175.29
135.96
128.74
128.43
128.28
77.16 CDCl3
66.51
62.89
49.13
21.80
11.80
OH
CO2Bn
2c-Bn
13C-NMR (126 MHz, CDCl3)
f1 (ppm)

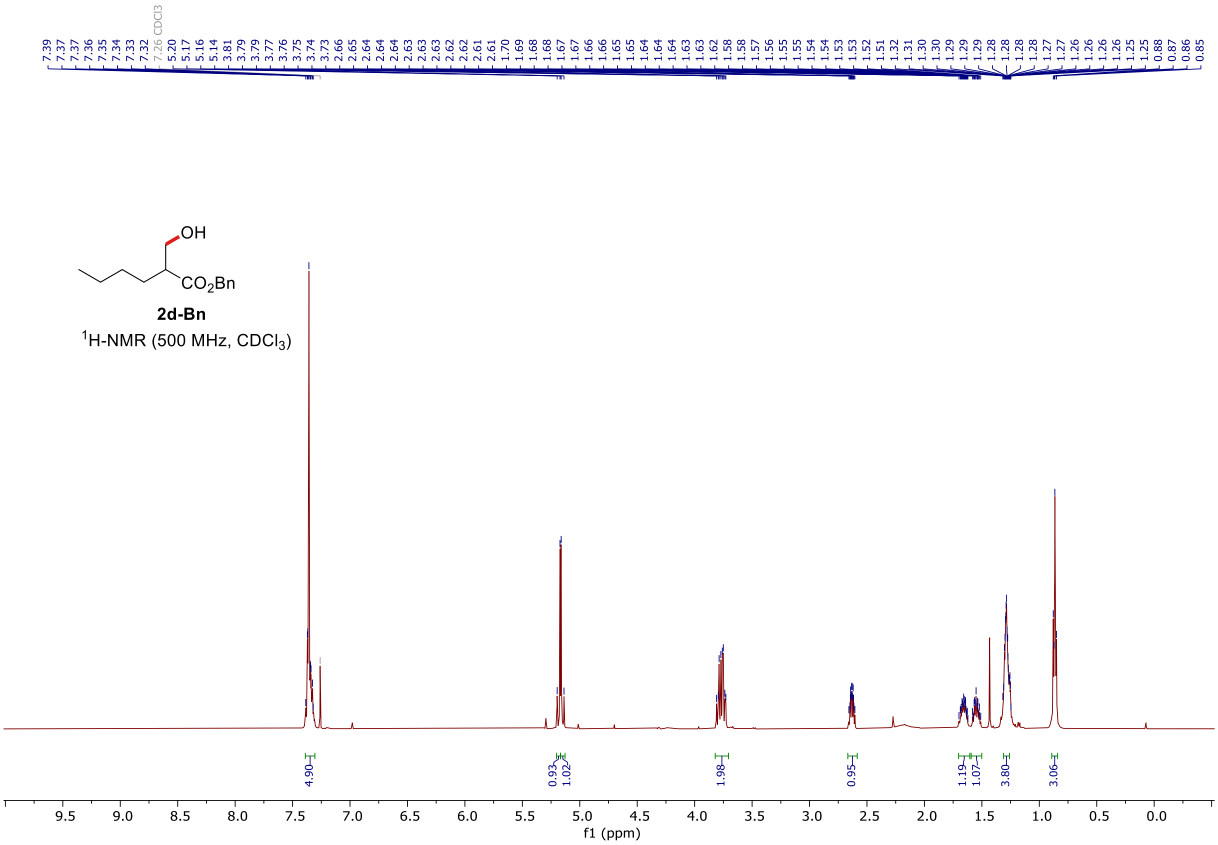
7.39 7.37 7.37 7.36 7.35 7.34 7.33 7.32 7.26 CDCl3 5.20 5.17 5.16 5.14 3.81 3.79 3.79 3.77 3.76 3.75 3.74 3.73 2.66 2.65 2.64 2.64 2.63 2.63 2.63 2.62 2.62 2.61 1.70 1.69 1.68 1.68 1.67 1.67 1.66 1.65 1.65 1.64 1.64 1.63 1.63 1.62 1.58 1.58 1.57 1.56 1.55 1.55 1.54 1.54 1.53 1.53 1.52 1.51 1.32 1.31 1.30 1.30 1.29 1.29 1.29 1.28 1.28 1.28 1.28 1.28 1.27 1.27 1.26 1.26 1.26 1.26 1.25 1.25 0.88 0.87 0.86 0.85
OH
CO2Bn
2d-Bn
1H-NMR (500 MHz, CDCl3)
4.90
0.93
1.02
1.98
0.95
1.19
1.07
3.80
3.06
f1 (ppm)

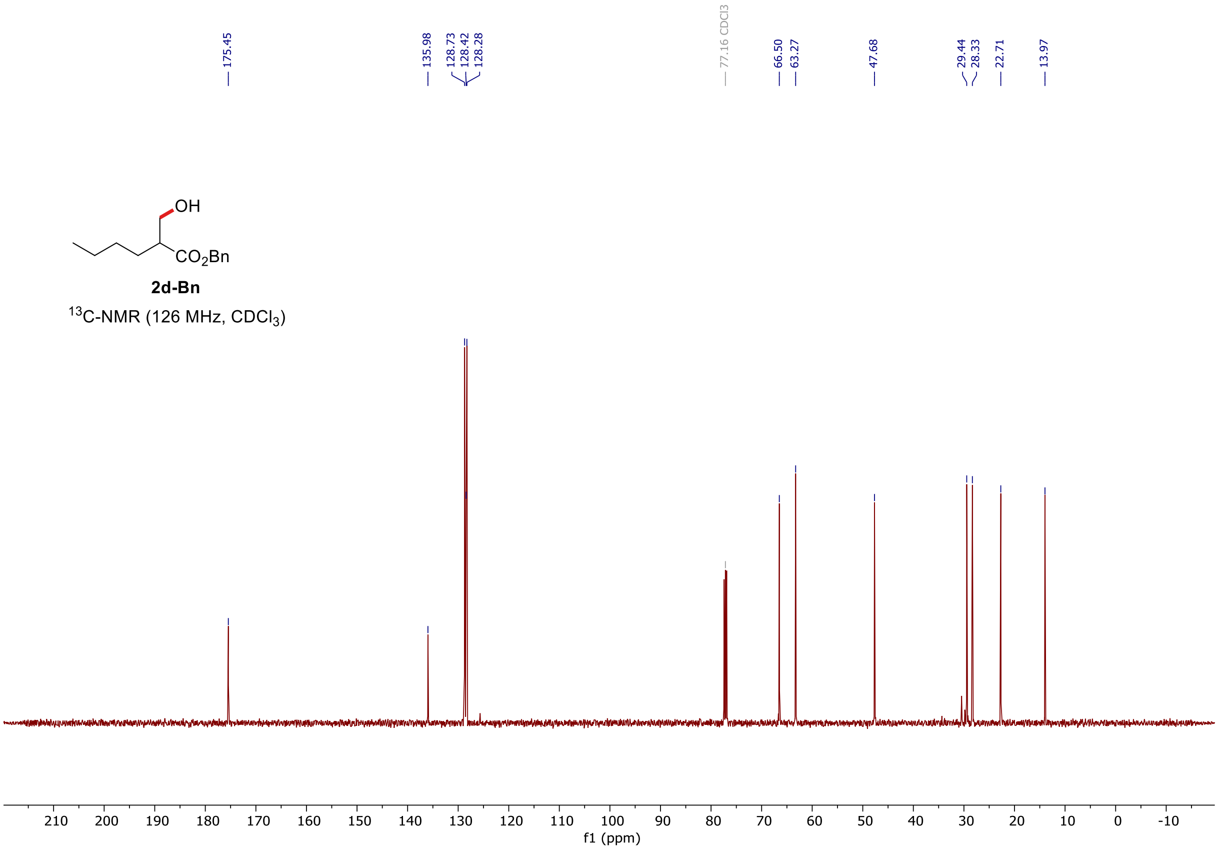
175.45
135.98
128.73
128.42
128.28
77.16 CDCl3
66.50
63.27
47.68
29.44
28.33
22.71
13.97
OH
CO2Bn
2d-Bn
13C-NMR (126 MHz, CDCl3)
f1 (ppm)

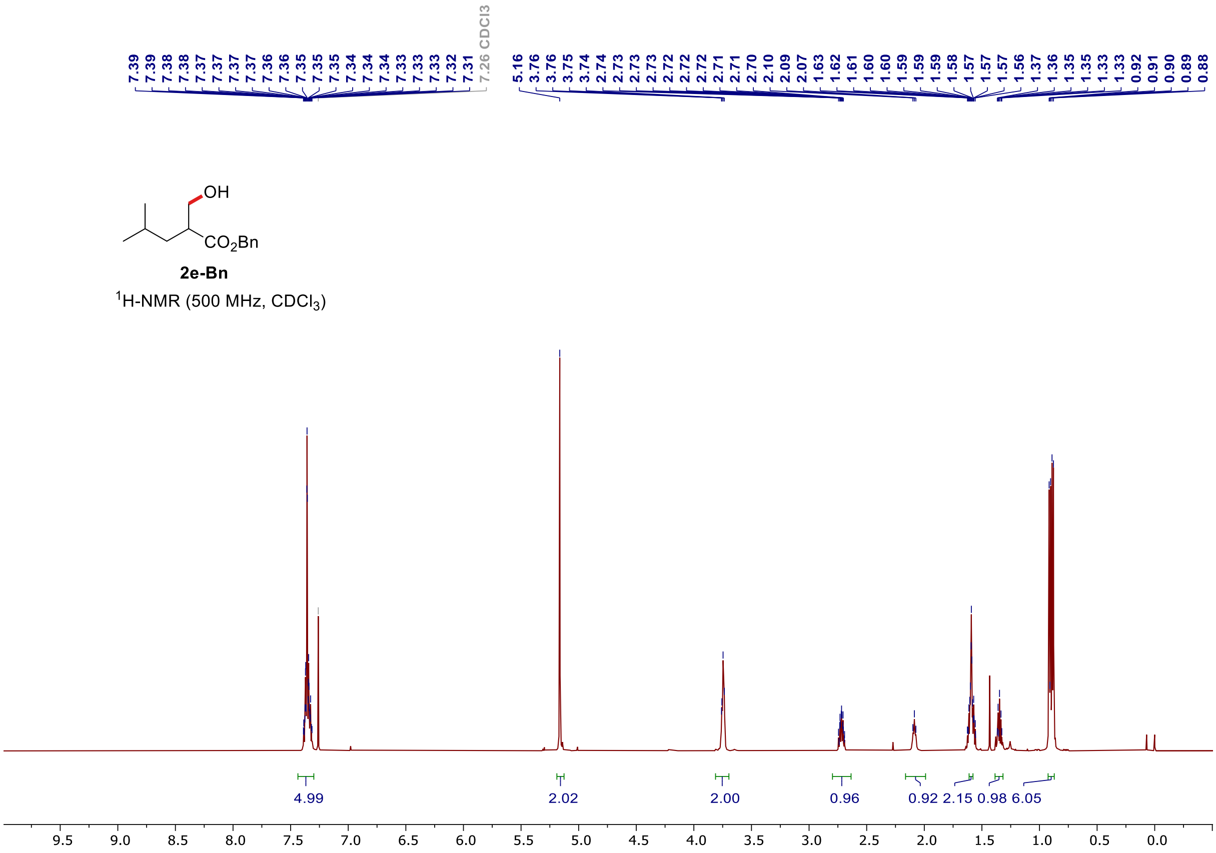

2e-Bn

^{1}H-NMR (500 MHz, $CDCl_3$)

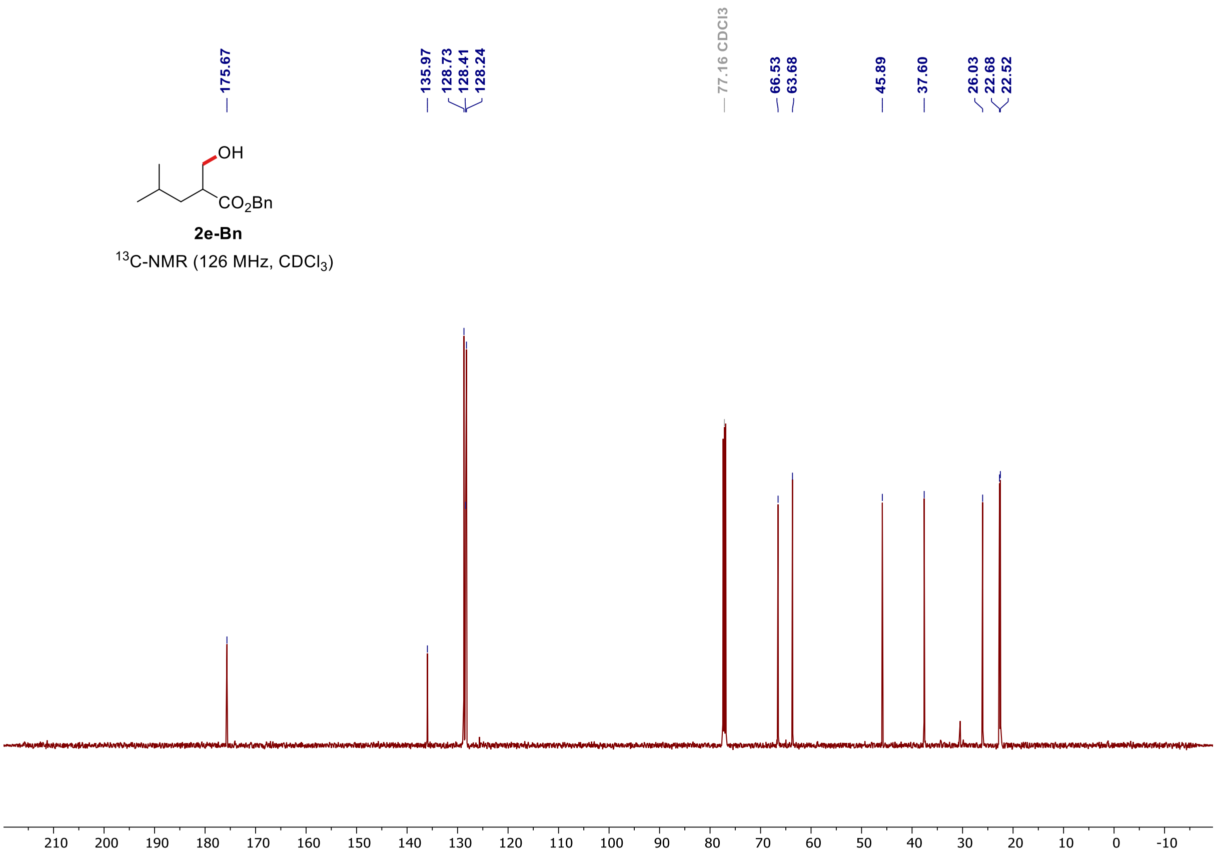

2e-Bn

^{13}C-NMR (126 MHz, $CDCl_3$)

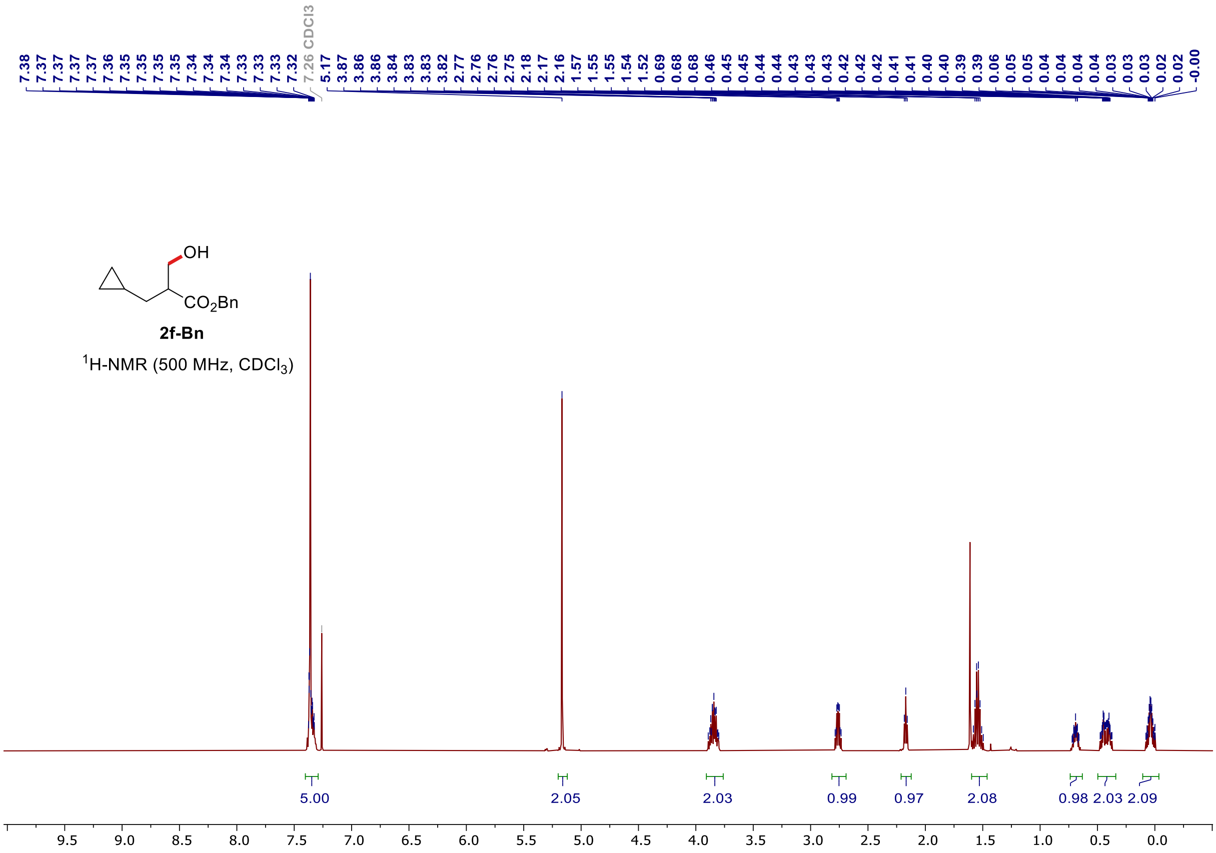
OH
CO2Bn
2f-Bn
1H-NMR (500 MHz, CDCl3)

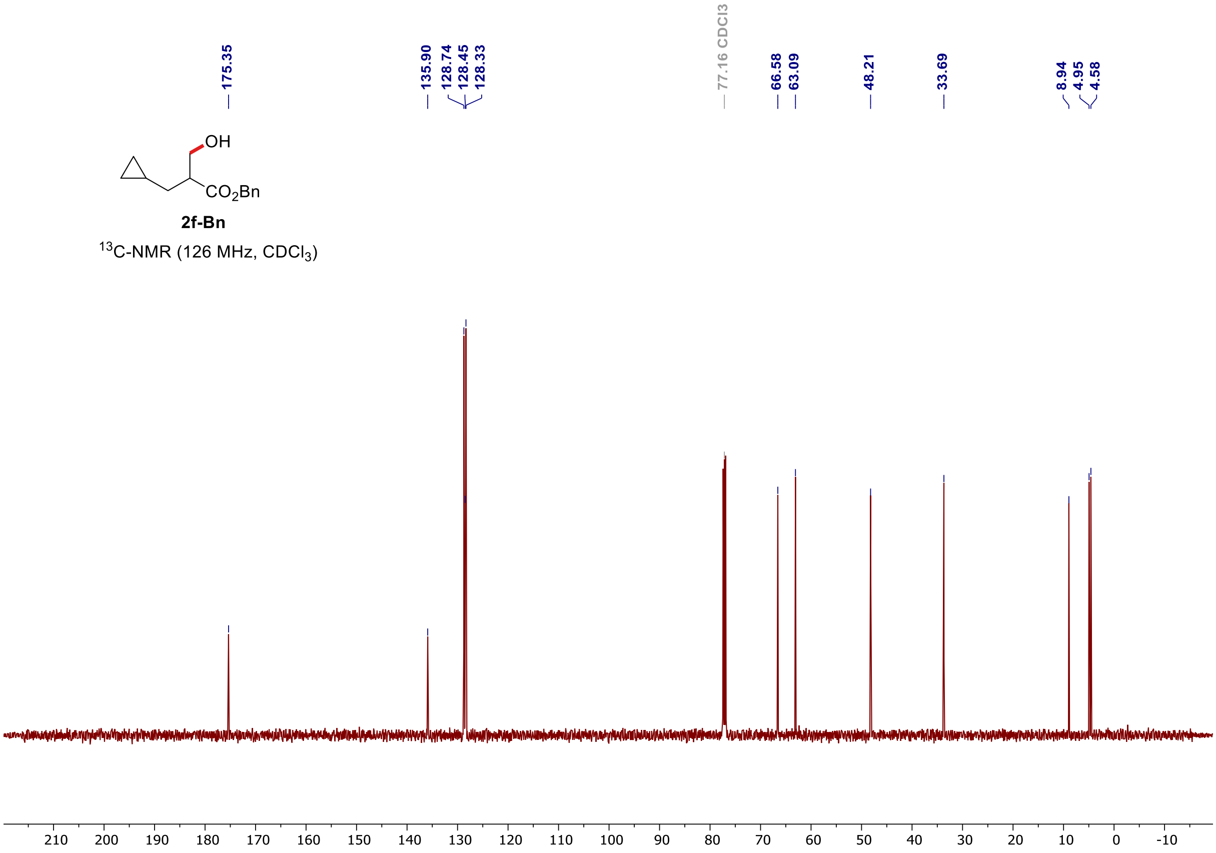
OH
CO2Bn
2f-Bn
13C-NMR (126 MHz, CDCl3)

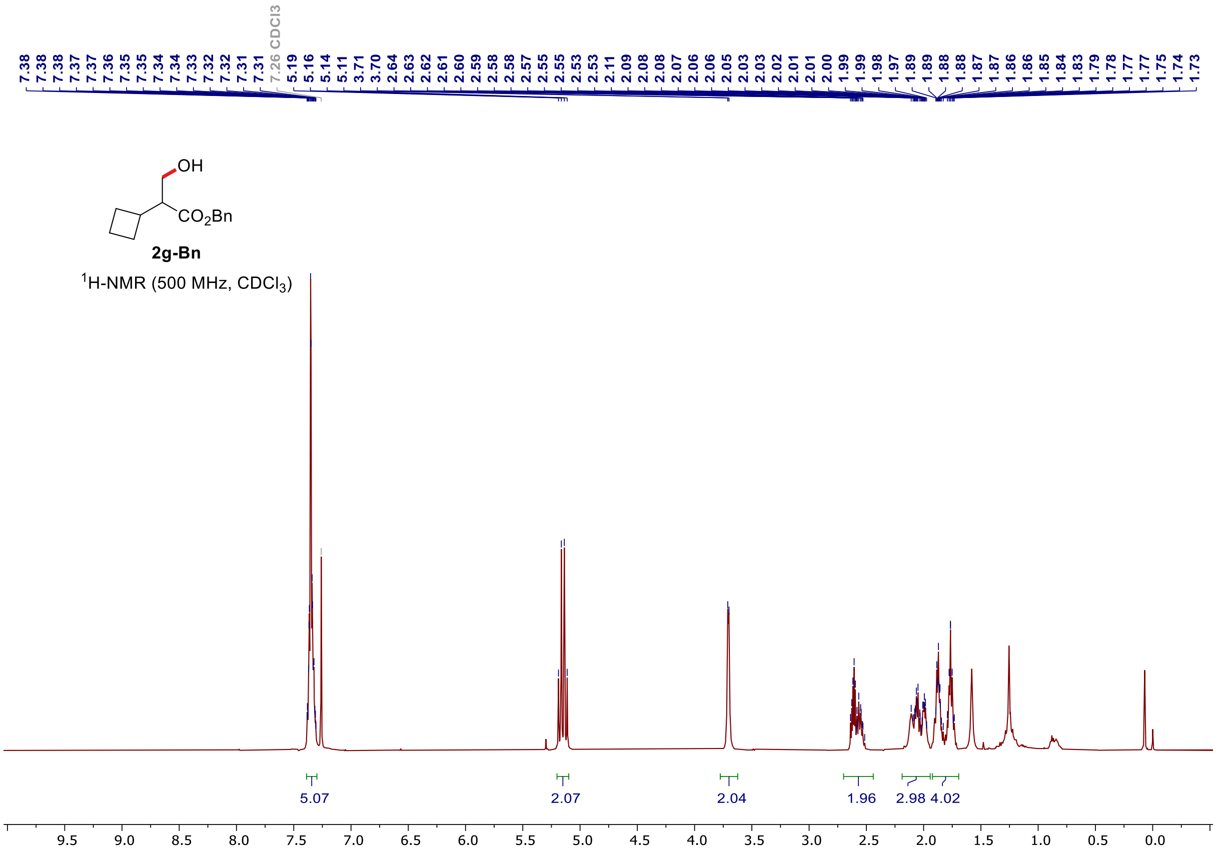
7.38 7.38 7.37 7.37 7.36 7.35 7.35 7.34 7.34 7.33 7.32 7.32 7.31 7.31 7.26 CDCl3 5.19 5.16 5.14 5.11 3.71 3.70 2.64 2.63 2.62 2.61 2.60 2.59 2.58 2.58 2.57 2.55 2.55 2.53 2.11 2.09 2.08 2.07 2.06 2.06 2.05 2.03 2.03 2.02 2.01 2.01 2.00 1.99 1.99 1.98 1.97 1.89 1.89 1.88 1.88 1.87 1.87 1.86 1.86 1.85 1.84 1.83 1.79 1.78 1.77 1.77 1.75 1.74 1.73
OH
CO_2Bn
2g-Bn
^{1}H-NMR (500 MHz, $CDCl_3$)
5.07 2.07 2.04 1.96 2.98 4.02
9.5 9.0 8.5 8.0 7.5 7.0 6.5 6.0 5.5 5.0 4.5 4.0 3.5 3.0 2.5 2.0 1.5 1.0 0.5 0.0

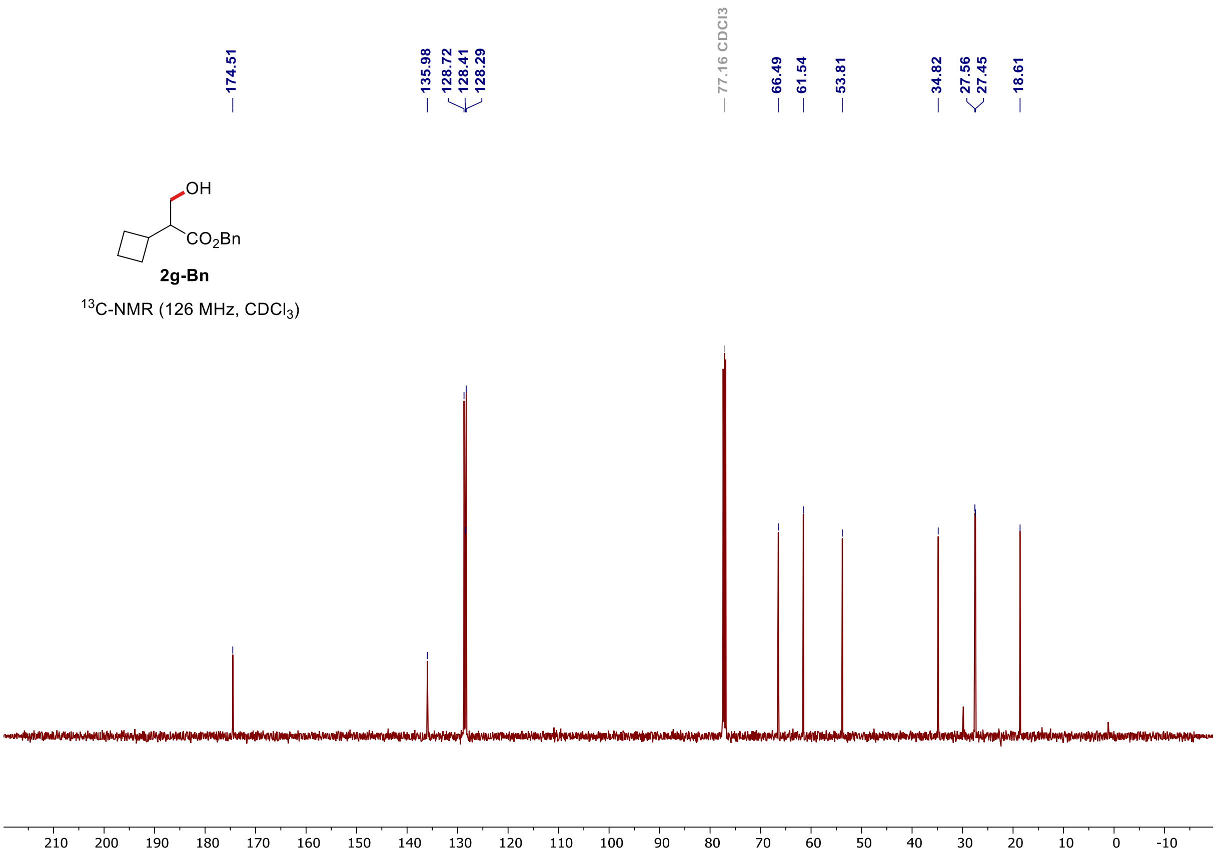
174.51 135.98 128.72 128.41 128.29 77.16 CDCl3 66.49 61.54 53.81 34.82 27.56 27.45 18.61
OH
CO_2Bn
2g-Bn
^{13}C-NMR (126 MHz, $CDCl_3$)
210 200 190 180 170 160 150 140 130 120 110 100 90 80 70 60 50 40 30 20 10 0 -10

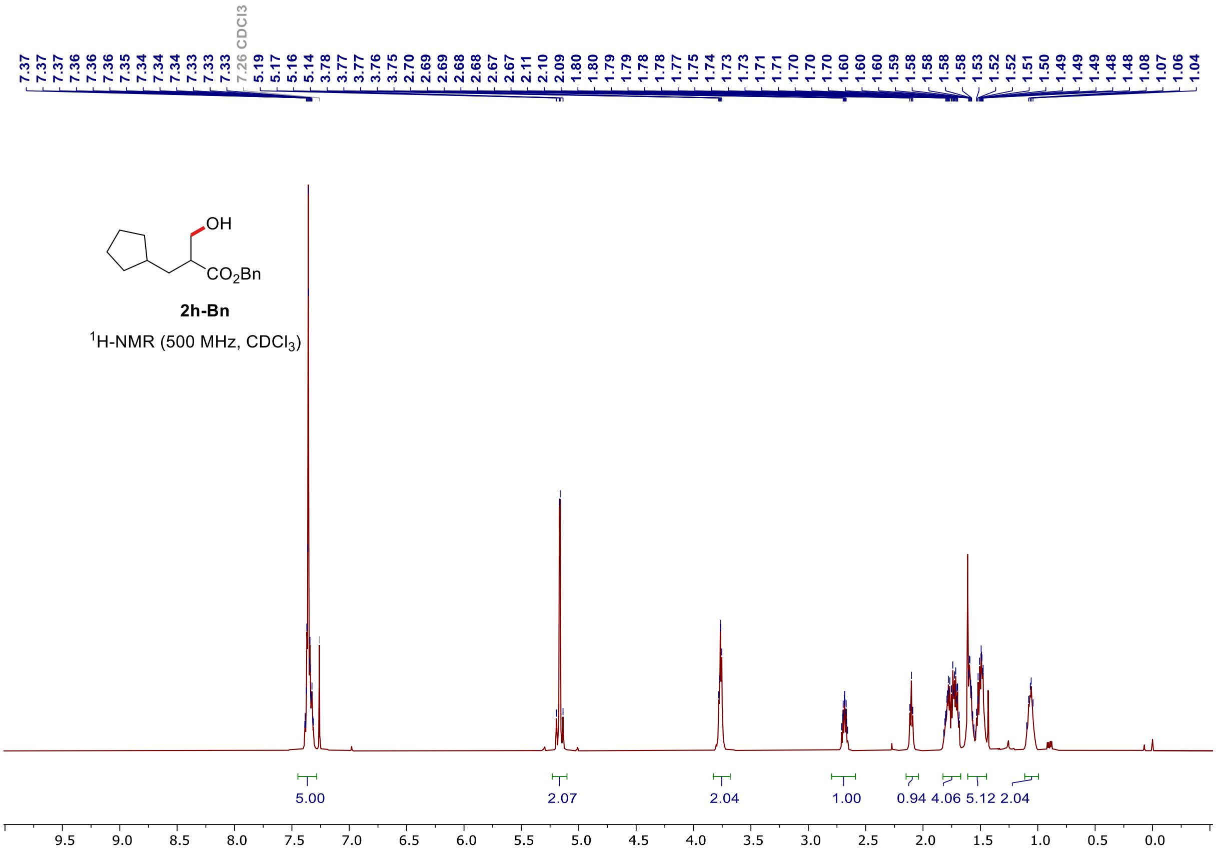
OH
CO2Bn
2h-Bn
1H-NMR (500 MHz, CDCl3)
5.00
2.07
2.04
1.00
0.94
4.06
5.12
2.04

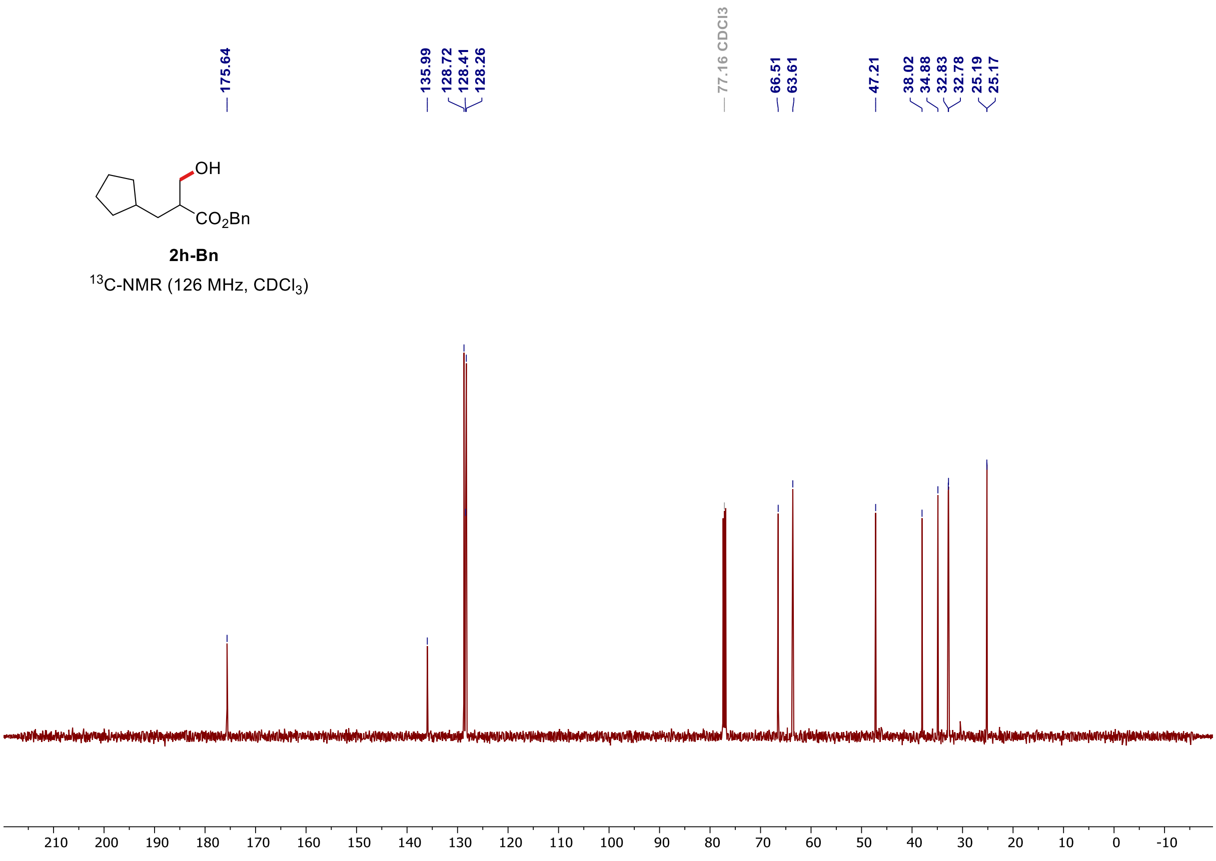
175.64
135.99
128.72
128.41
128.26
77.16 CDCl3
66.51
63.61
47.21
38.02
34.88
32.83
32.78
25.19
25.17
OH
CO2Bn
2h-Bn
13C-NMR (126 MHz, CDCl3)

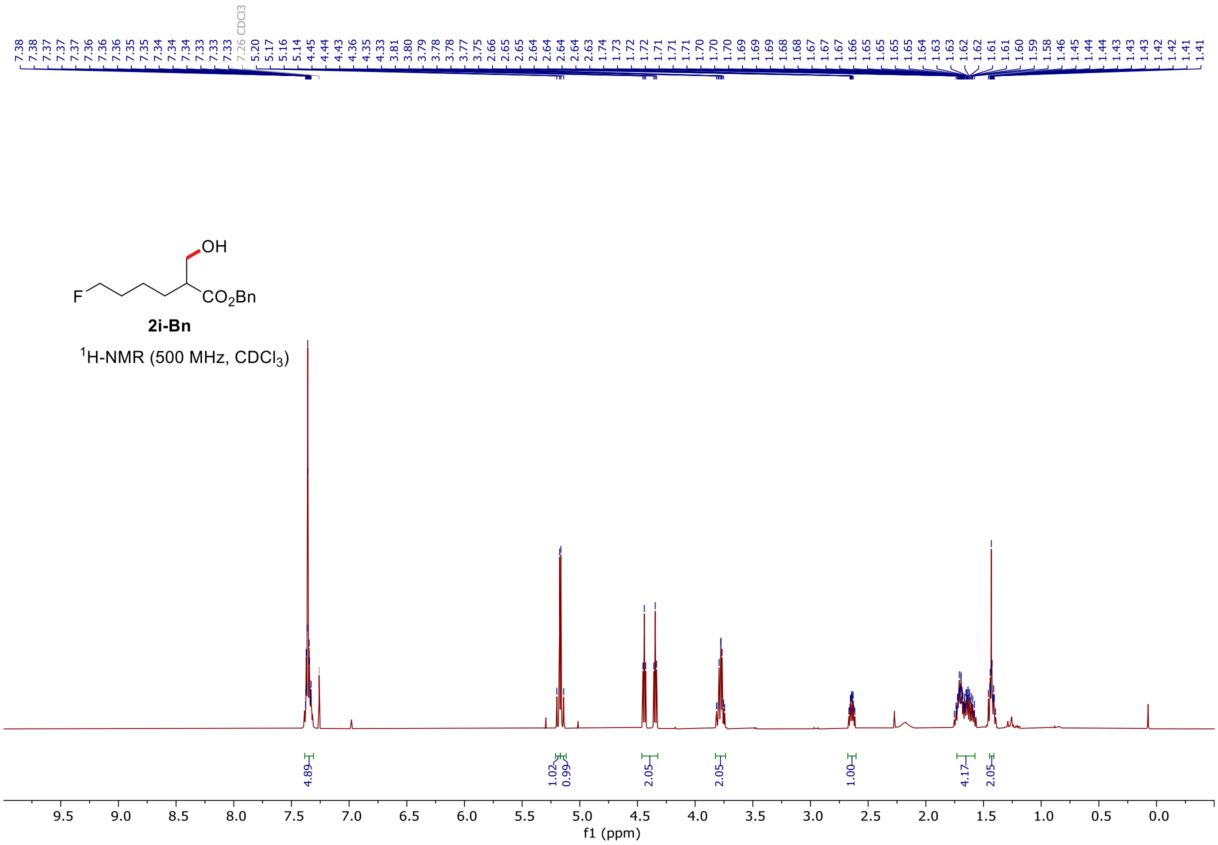
OH
F
CO2Bn
2i-Bn
1H-NMR (500 MHz, CDCl3)
f1 (ppm)

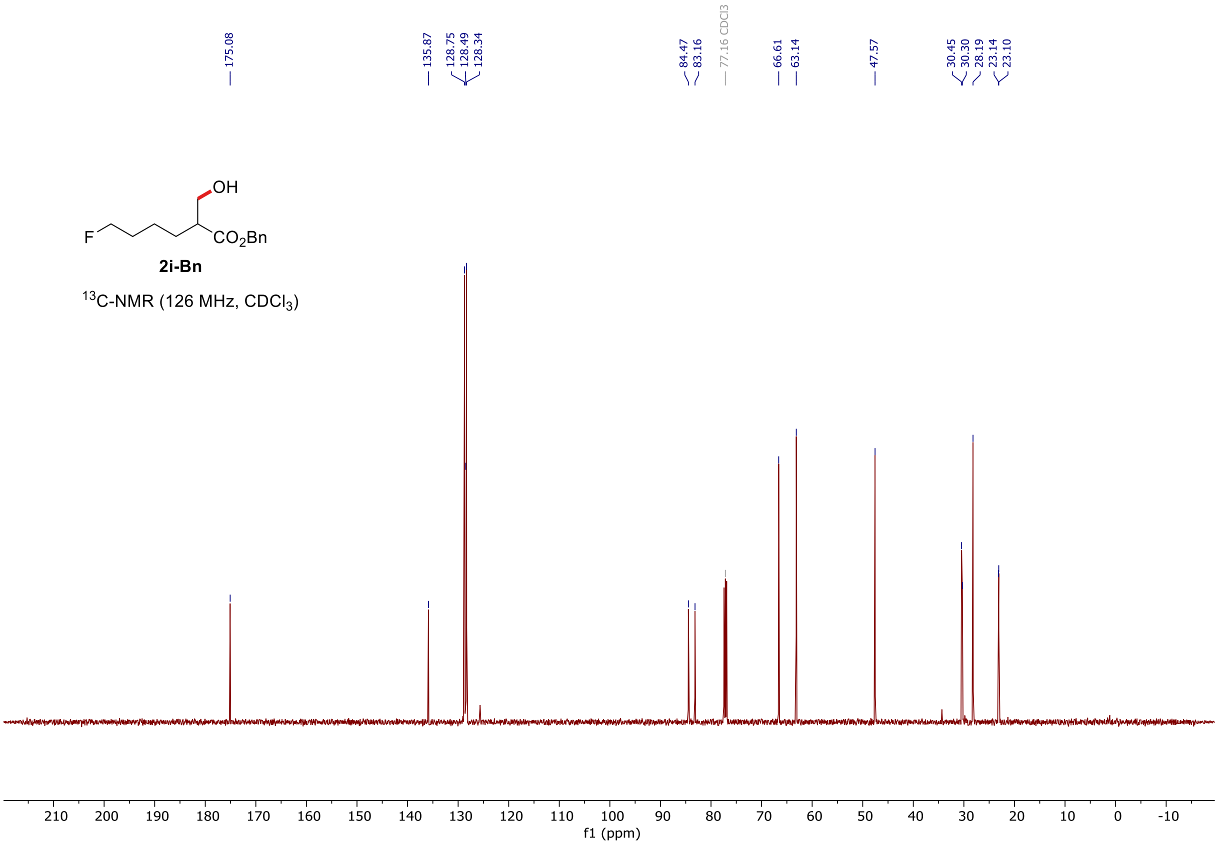
OH
F
CO2Bn
2i-Bn
13C-NMR (126 MHz, CDCl3)
f1 (ppm)

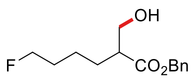

2i-Bn

^{19}F NMR (471 MHz, $CDCl_3$)

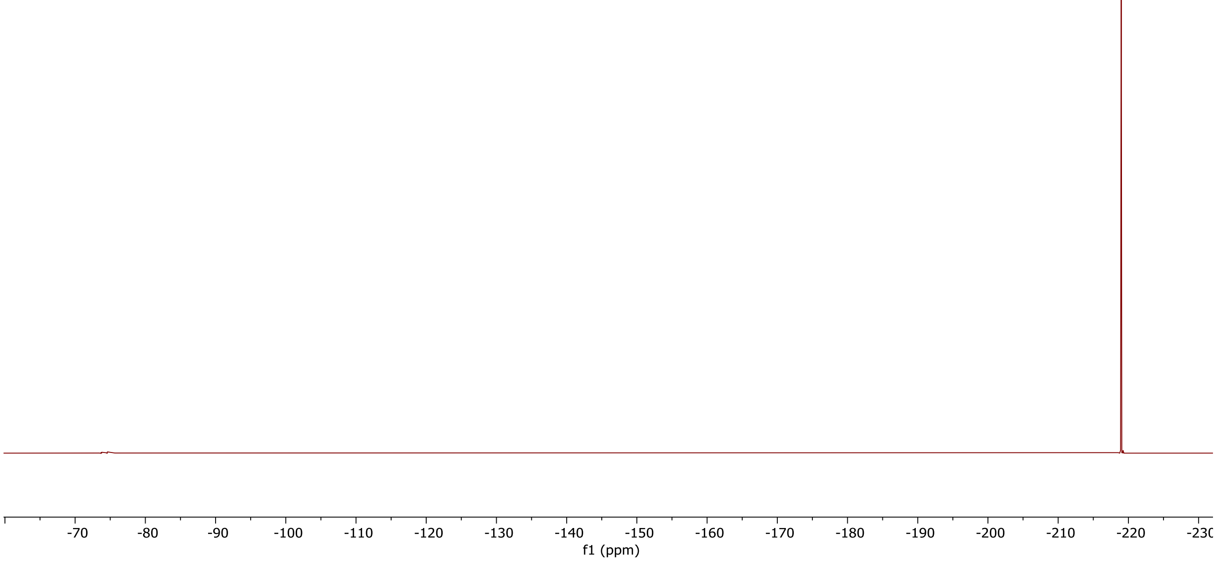

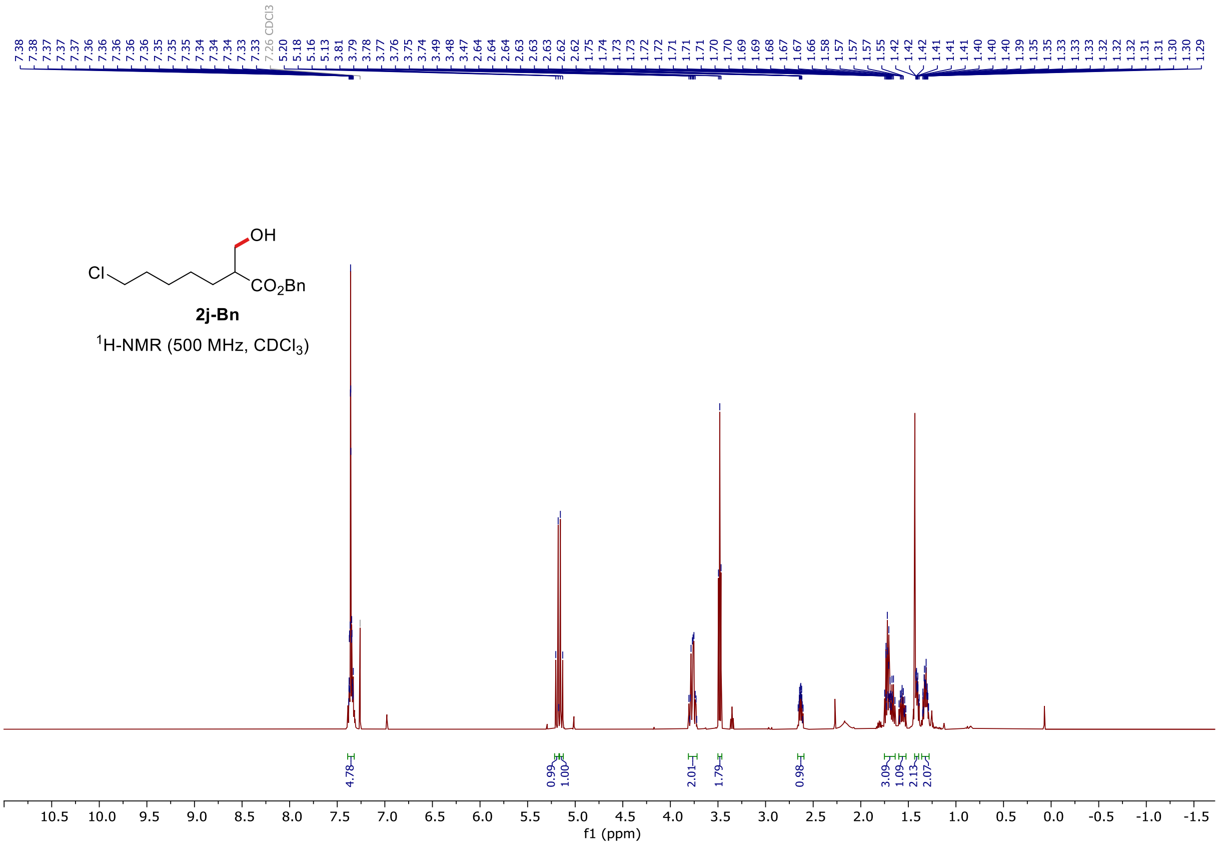
7.38 7.38 7.37 7.37 7.37 7.36 7.36 7.36 7.36 7.36 7.35 7.35 7.35 7.34 7.34 7.34 7.33 7.33 7.26 CDCl3 5.20 5.18 5.16 5.13 3.81 3.79 3.78 3.77 3.76 3.75 3.74 3.49 3.48 3.47 2.64 2.64 2.64 2.63 2.63 2.63 2.62 2.62 1.75 1.74 1.73 1.72 1.72 1.71 1.71 1.71 1.70 1.70 1.69 1.69 1.68 1.67 1.67 1.66 1.58 1.57 1.57 1.57 1.55 1.42 1.42 1.42 1.41 1.41 1.41 1.40 1.40 1.40 1.39 1.35 1.35 1.33 1.33 1.33 1.32 1.32 1.32 1.31 1.31 1.30 1.30 1.29
OH
Cl
CO_2Bn
2j-Bn
^{1}H-NMR (500 MHz, $CDCl_3$)
4.78 0.99 1.00 2.01 1.79 0.98 3.09 1.09 2.13 2.07
10.5 10.0 9.5 9.0 8.5 8.0 7.5 7.0 6.5 6.0 5.5 5.0 4.5 4.0 3.5 3.0 2.5 2.0 1.5 1.0 0.5 0.0 -0.5 -1.0 -1.5
f1 (ppm)

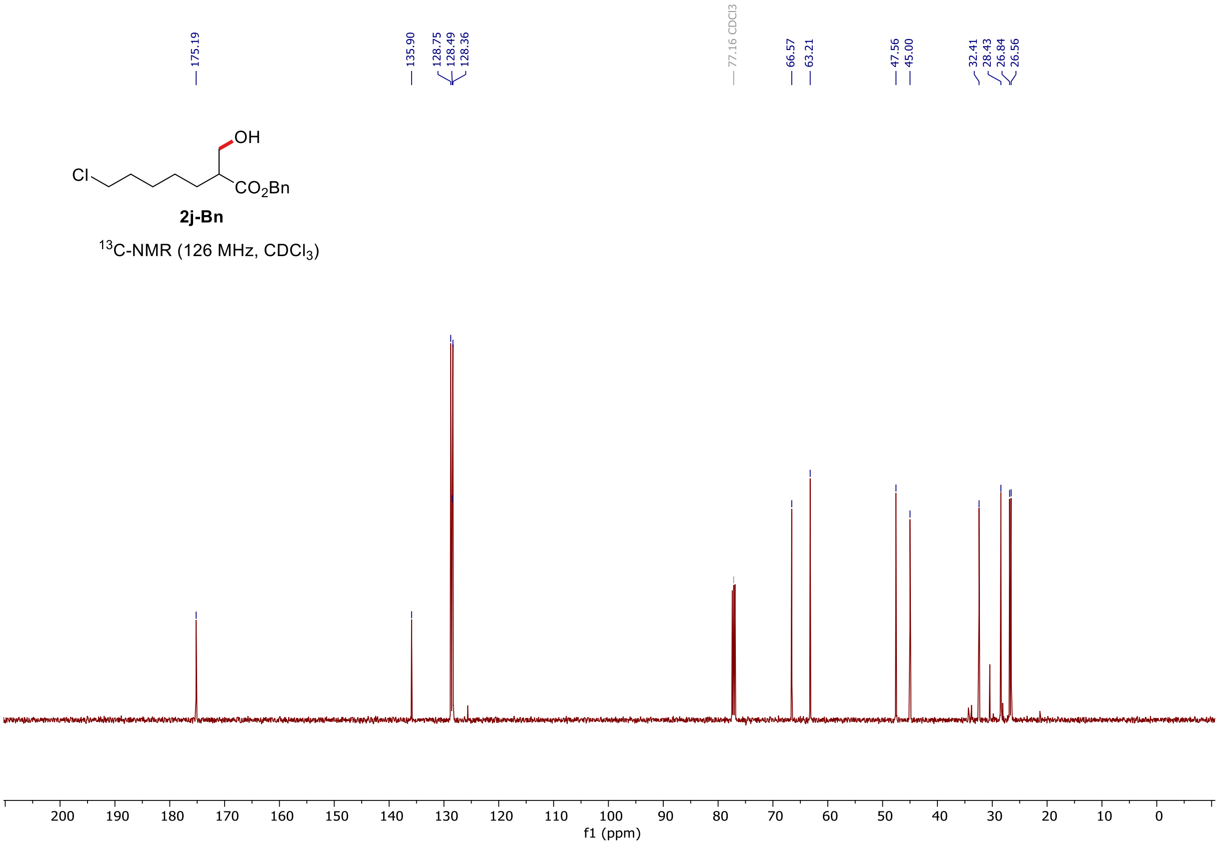
175.19
135.90
128.75 128.49 128.36
77.16 CDCl3
66.57
63.21
47.56
45.00
32.41 28.43 26.84 26.56
OH
Cl
CO_2Bn
2j-Bn
^{13}C-NMR (126 MHz, $CDCl_3$)
200 190 180 170 160 150 140 130 120 110 100 90 80 70 60 50 40 30 20 10 0
f1 (ppm)

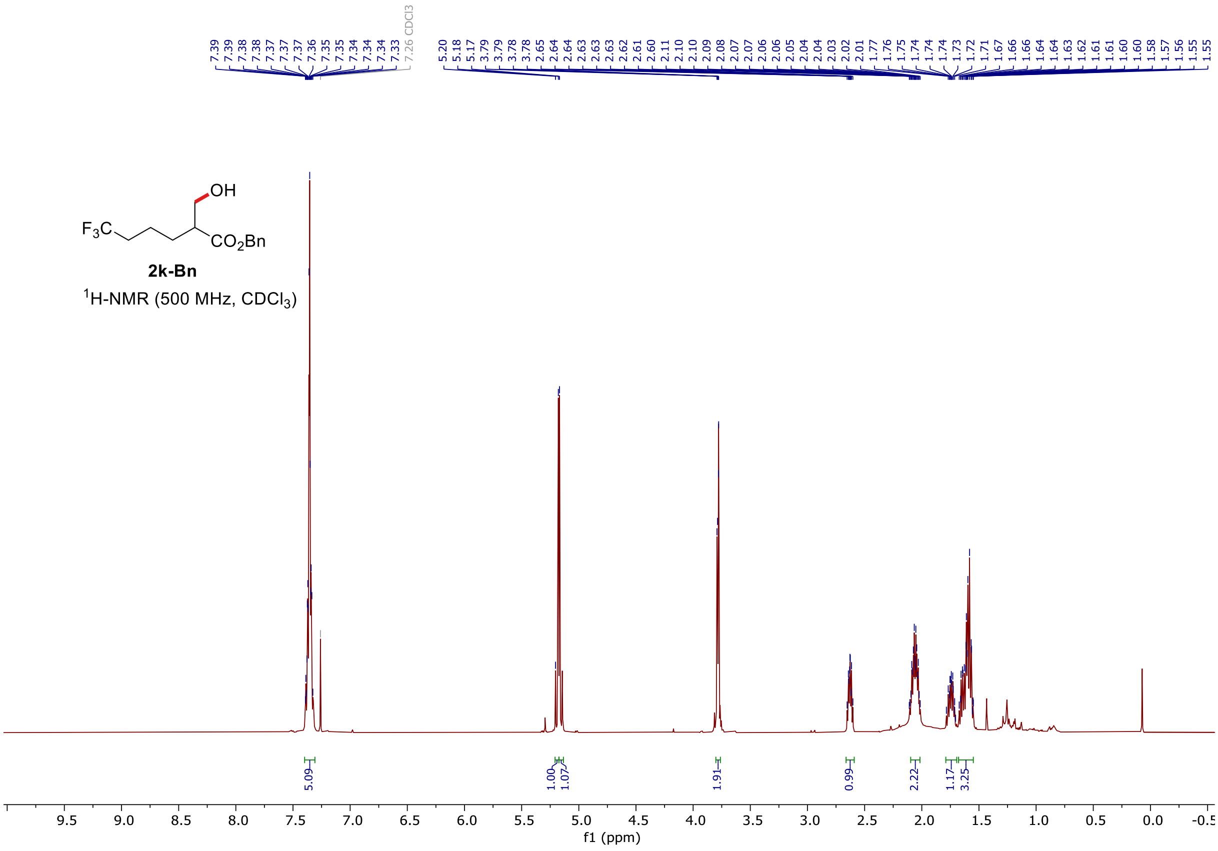
OH
F3C
CO2Bn
2k-Bn
1H-NMR (500 MHz, CDCl3)
f1 (ppm)

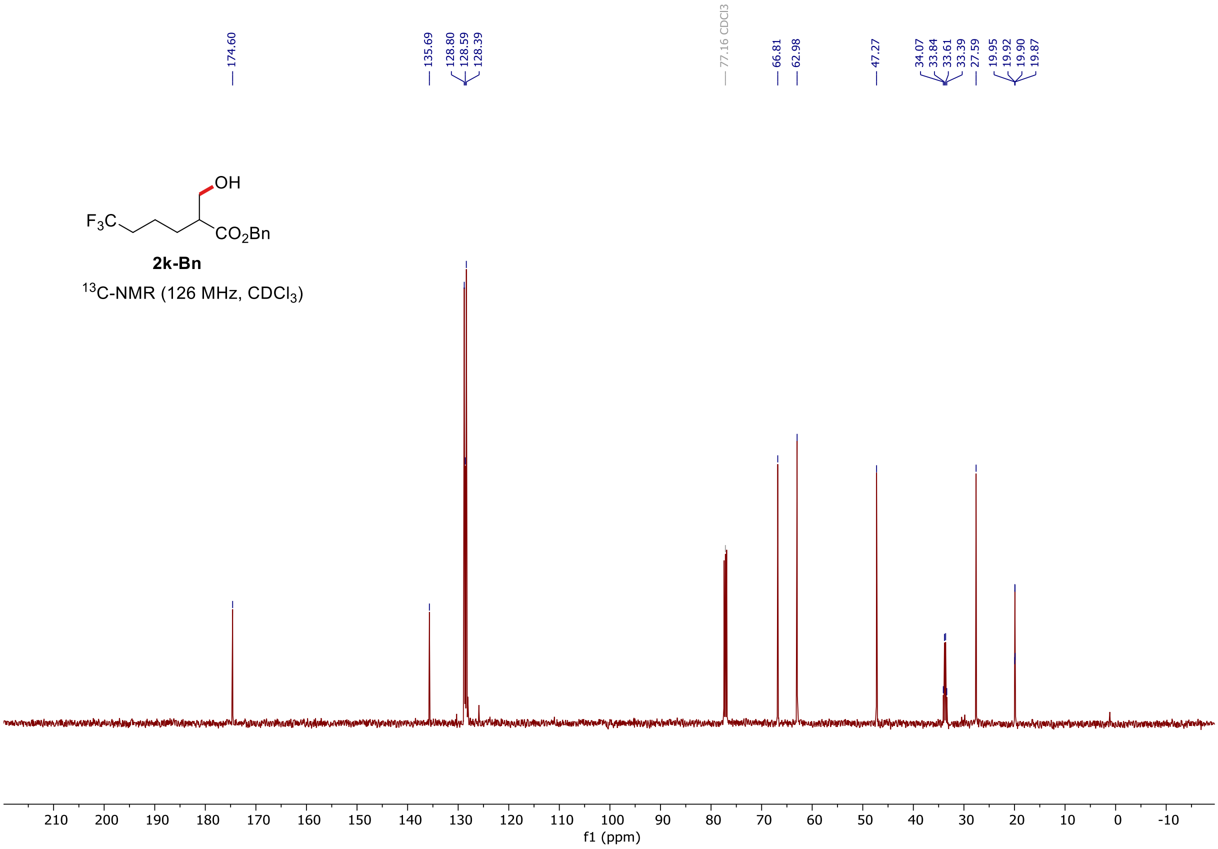
174.60
135.69
128.80
128.59
128.39
77.16 CDCl3
66.81
62.98
47.27
34.07
33.84
33.61
33.39
27.59
19.95
19.92
19.90
19.87
OH
F3C
CO2Bn
2k-Bn
13C-NMR (126 MHz, CDCl3)
f1 (ppm)

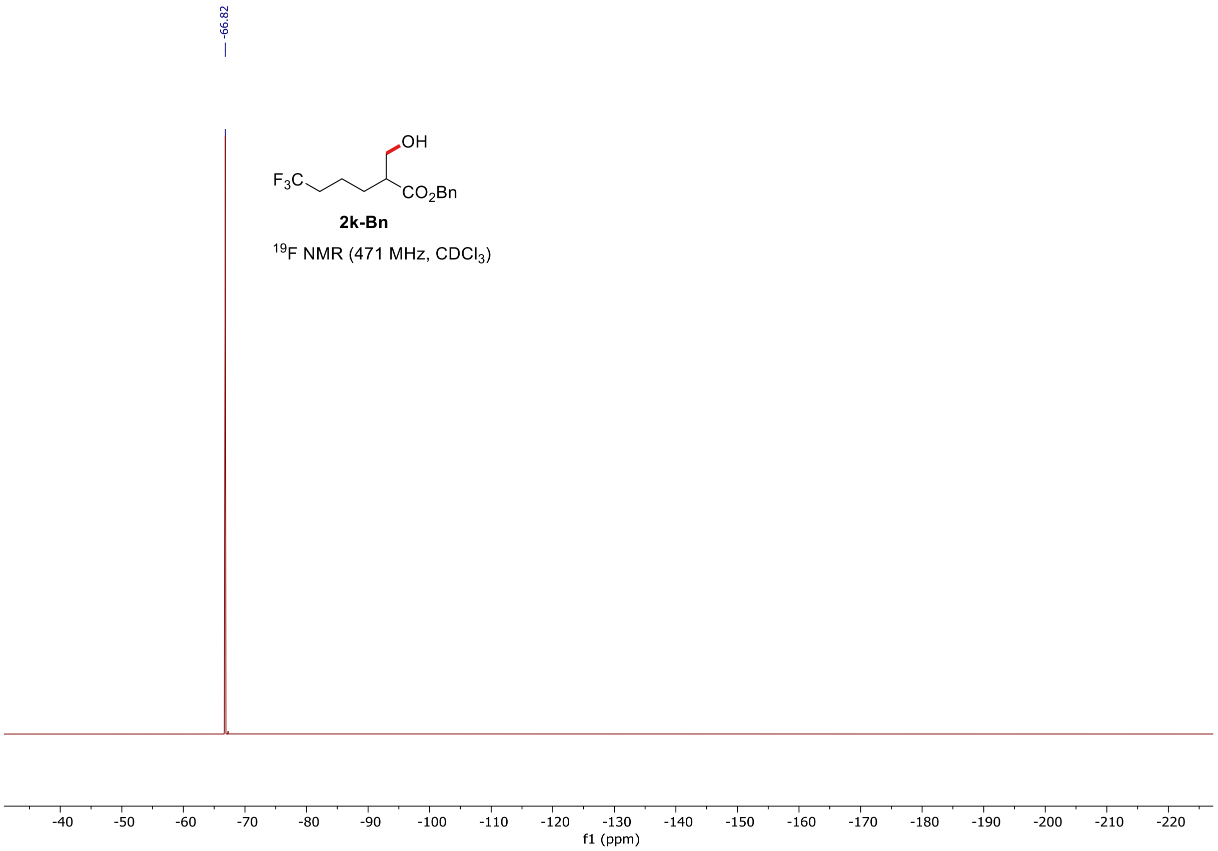
-66.82
F3C
OH
CO2Bn
2k-Bn
19F NMR (471 MHz, CDCl3)
-40
-50
-60
-70
-80
-90
-100
-110
-120
-130
-140
-150
-160
-170
-180
-190
-200
-210
-220
f1 (ppm)

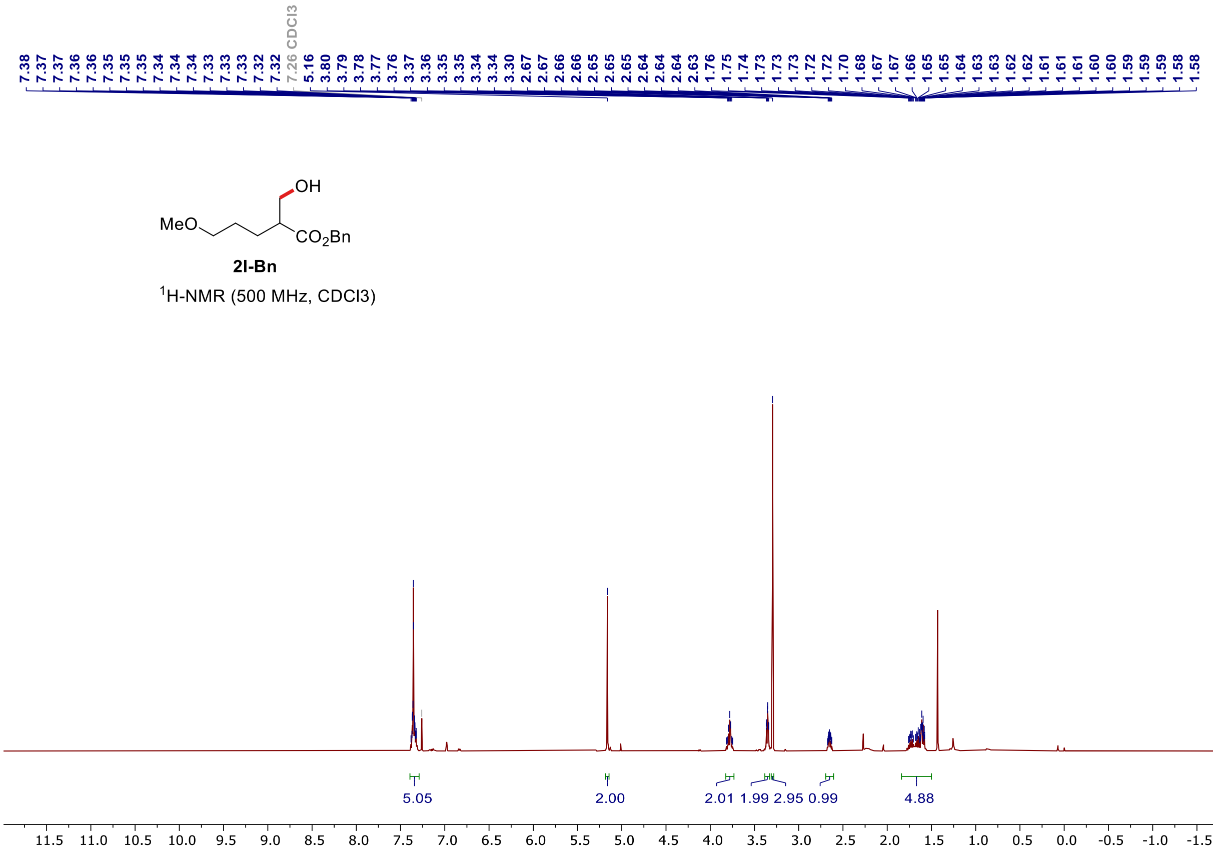
7.38 7.37 7.37 7.36 7.36 7.35 7.35 7.35 7.34 7.34 7.34 7.33 7.33 7.33 7.32 7.32 7.26 CDCl3 5.16 3.80 3.79 3.78 3.77 3.76 3.37 3.36 3.35 3.35 3.34 3.34 3.30 2.67 2.67 2.66 2.65 2.65 2.65 2.64 2.64 2.63 1.76 1.75 1.74 1.73 1.73 1.73 1.72 1.72 1.70 1.68 1.67 1.67 1.66 1.65 1.65 1.64 1.63 1.63 1.62 1.62 1.61 1.61 1.61 1.60 1.59 1.59 1.59 1.58
MeO
OH
CO2Bn
2l-Bn
1H-NMR (500 MHz, CDCl3)
5.05
2.00
2.01 1.99 2.95 0.99
4.88
11.5 11.0 10.5 10.0 9.5 9.0 8.5 8.0 7.5 7.0 6.5 6.0 5.5 5.0 4.5 4.0 3.5 3.0 2.5 2.0 1.5 1.0 0.5 0.0 -0.5 -1.0 -1.5

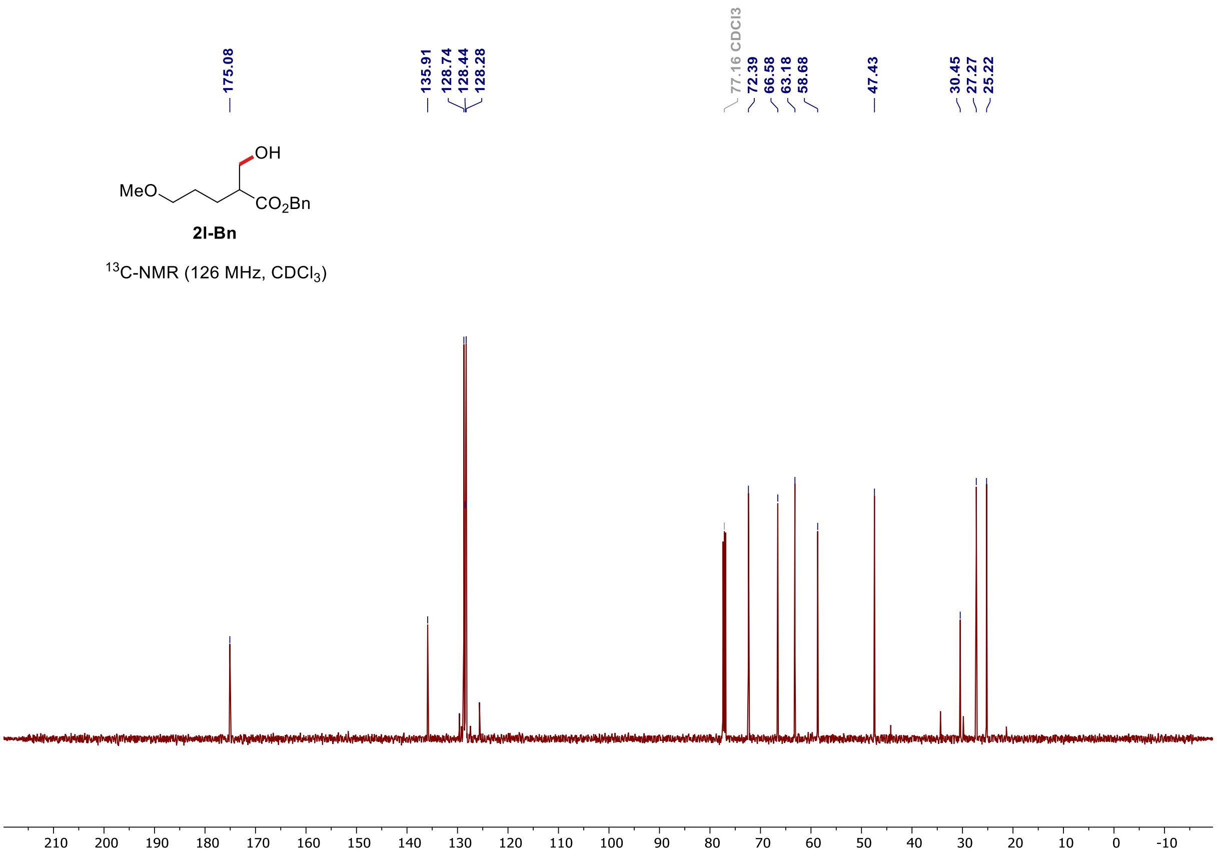
175.08
135.91 128.74 128.44 128.28
77.16 CDCl3 72.39 66.58 63.18 58.68
47.43
30.45 27.27 25.22
MeO
OH
CO2Bn
2l-Bn
13C-NMR (126 MHz, CDCl3)
210 200 190 180 170 160 150 140 130 120 110 100 90 80 70 60 50 40 30 20 10 0 -10

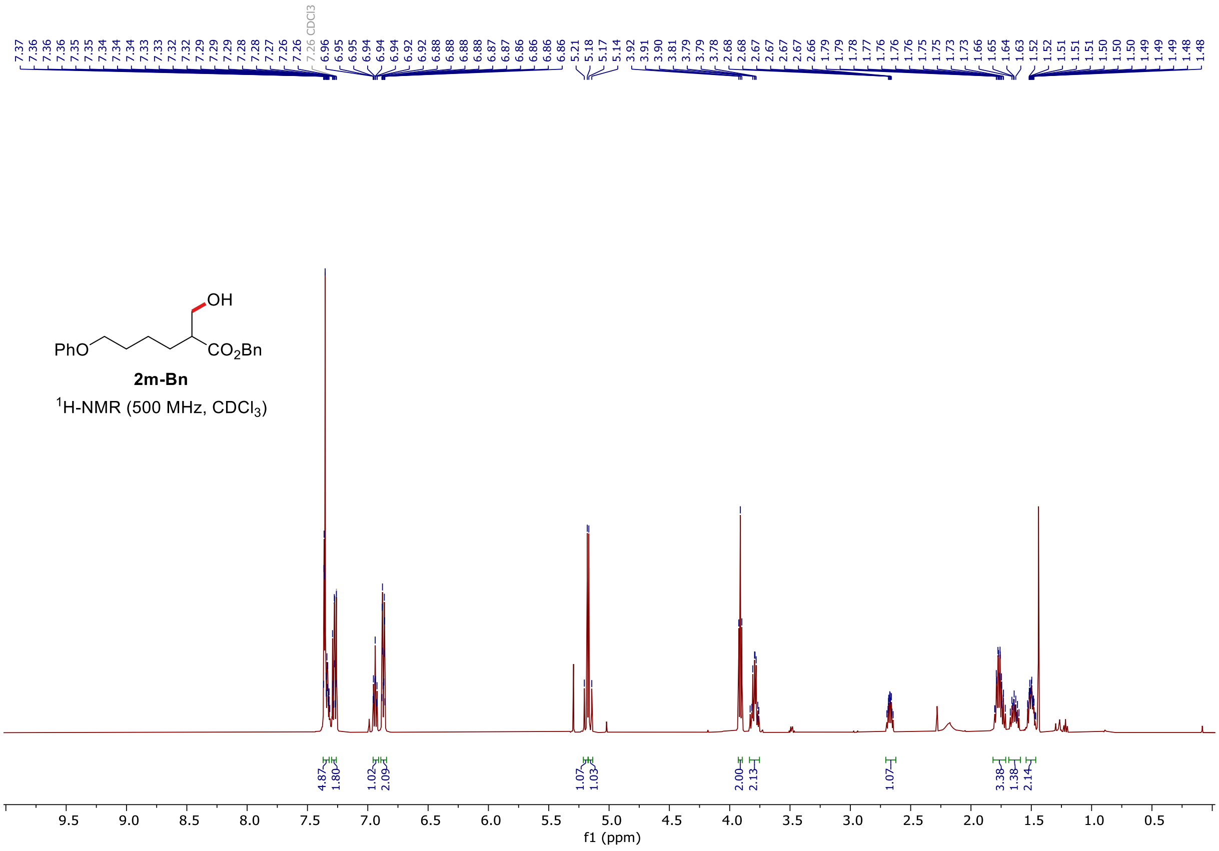
OH
PhO
CO2Bn
2m-Bn
1H-NMR (500 MHz, CDCl3)
f1 (ppm)

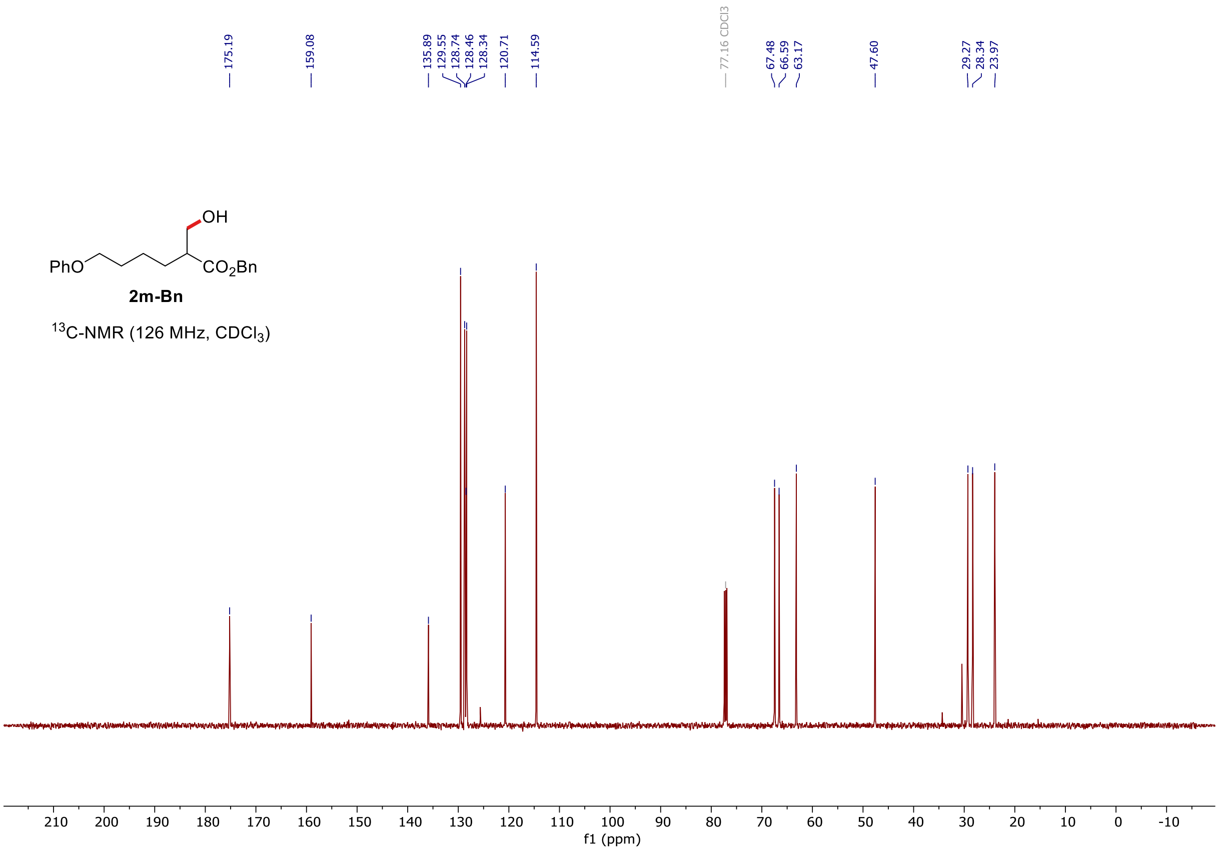
OH
PhO
CO2Bn
2m-Bn
13C-NMR (126 MHz, CDCl3)
175.19
159.08
135.89
129.55
128.74
128.46
128.34
120.71
114.59
77.16 CDCl3
67.48
66.59
63.17
47.60
29.27
28.34
23.97
f1 (ppm)

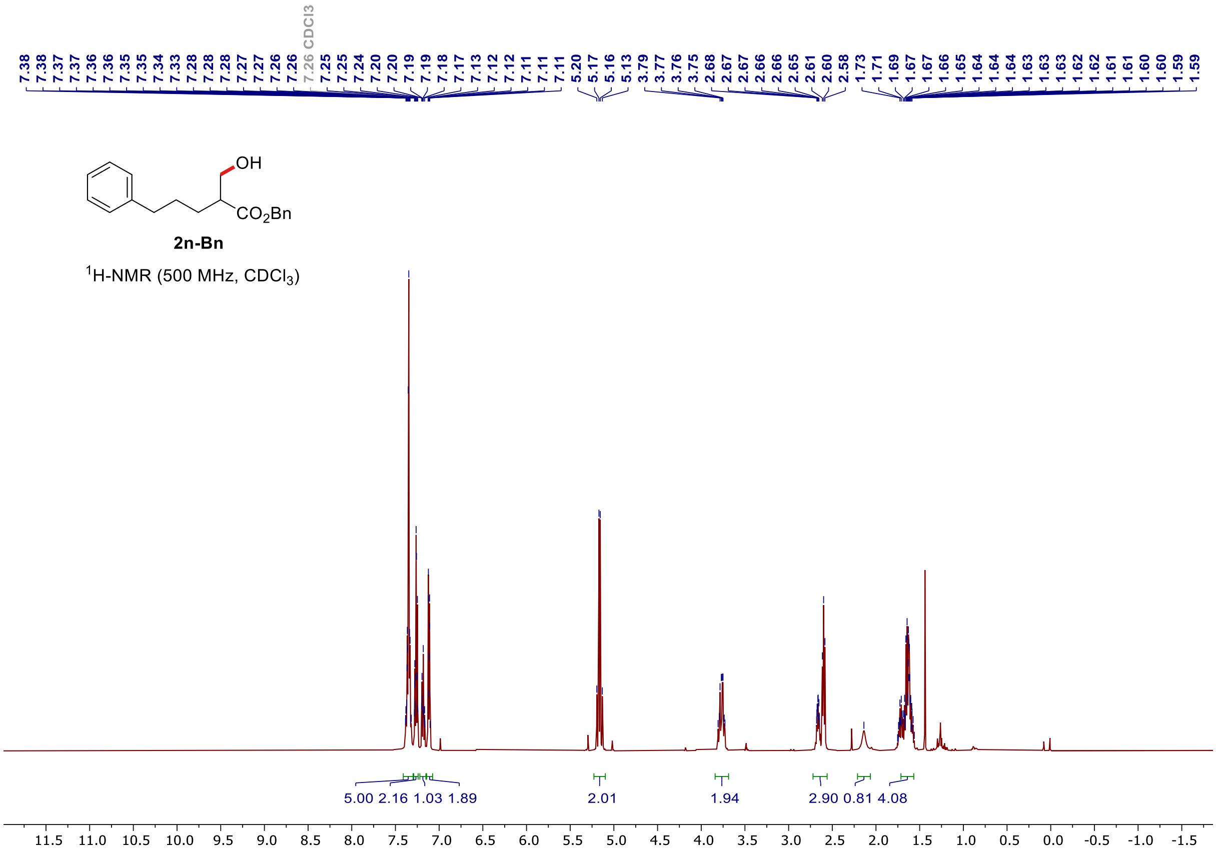
7.38 7.38 7.37 7.36 7.36 7.35 7.35 7.34 7.33 7.28 7.28 7.28 7.27 7.27 7.26 7.26 7.26 CDCl3 7.25 7.25 7.24 7.20 7.20 7.19 7.19 7.18 7.17 7.13 7.12 7.12 7.11 7.11 7.11 5.20 5.17 5.16 5.13 3.79 3.77 3.76 3.75 2.68 2.67 2.67 2.66 2.65 2.61 2.60 2.58 1.73 1.71 1.69 1.67 1.67 1.66 1.65 1.64 1.64 1.64 1.63 1.63 1.63 1.62 1.62 1.61 1.61 1.60 1.60 1.59 1.59
OH
CO2Bn
2n-Bn
1H-NMR (500 MHz, CDCl3)
5.00 2.16 1.03 1.89
2.01
1.94
2.90 0.81 4.08
11.5 11.0 10.5 10.0 9.5 9.0 8.5 8.0 7.5 7.0 6.5 6.0 5.5 5.0 4.5 4.0 3.5 3.0 2.5 2.0 1.5 1.0 0.5 0.0 -0.5 -1.0 -1.5

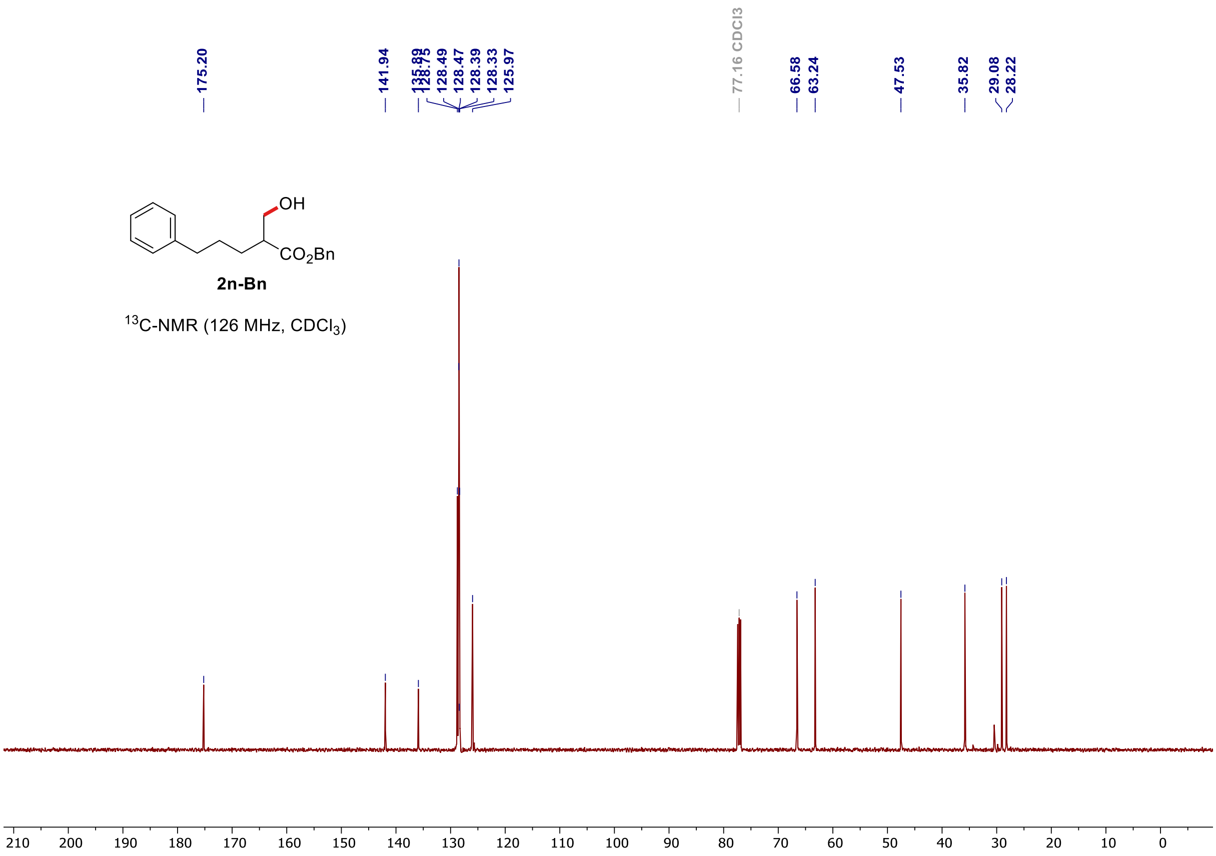
175.20
141.94
135.89 128.75 128.49 128.47 128.39 128.33 125.97
77.16 CDCl3
66.58
63.24
47.53
35.82
29.08 28.22
OH
CO2Bn
2n-Bn
13C-NMR (126 MHz, CDCl3)
210 200 190 180 170 160 150 140 130 120 110 100 90 80 70 60 50 40 30 20 10 0

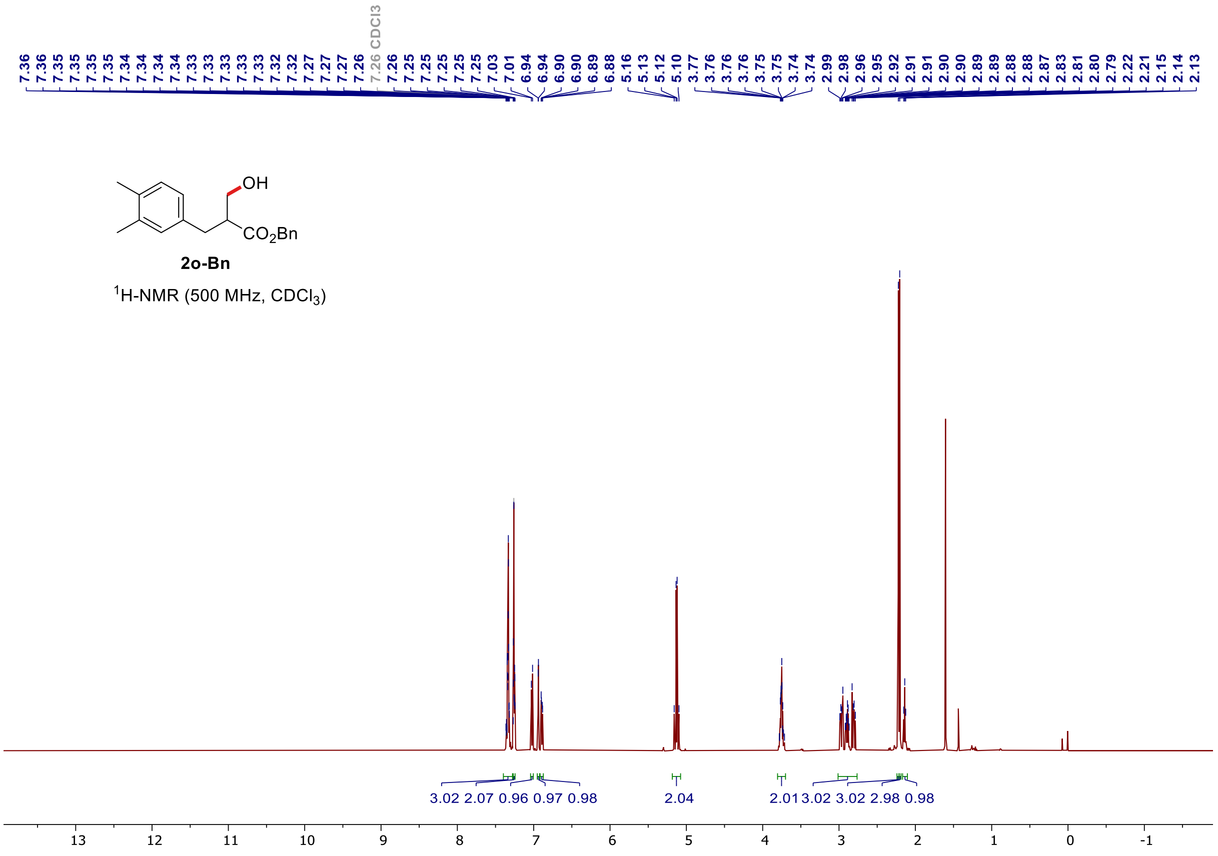

7.36 7.36 7.35 7.35 7.35 7.35 7.34 7.34 7.34 7.34 7.33 7.33 7.33 7.33 7.33 7.32 7.32 7.27 7.27 7.27 7.26 7.26 CDCl3 7.26 7.25 7.25 7.25 7.25 7.25 7.03 7.01 6.94 6.94 6.90 6.90 6.89 6.88 5.16 5.13 5.12 5.10 3.77 3.76 3.76 3.76 3.75 3.75 3.74 3.74 2.99 2.98 2.96 2.95 2.92 2.91 2.91 2.90 2.90 2.89 2.89 2.88 2.88 2.87 2.83 2.81 2.80 2.79 2.22 2.21 2.15 2.14 2.13
OH
CO_2Bn
2o-Bn
^{1}H-NMR (500 MHz, $CDCl_3$)
3.02 2.07 0.96 0.97 0.98
2.04
2.01 3.02 3.02 2.98 0.98
13 12 11 10 9 8 7 6 5 4 3 2 1 0 -1

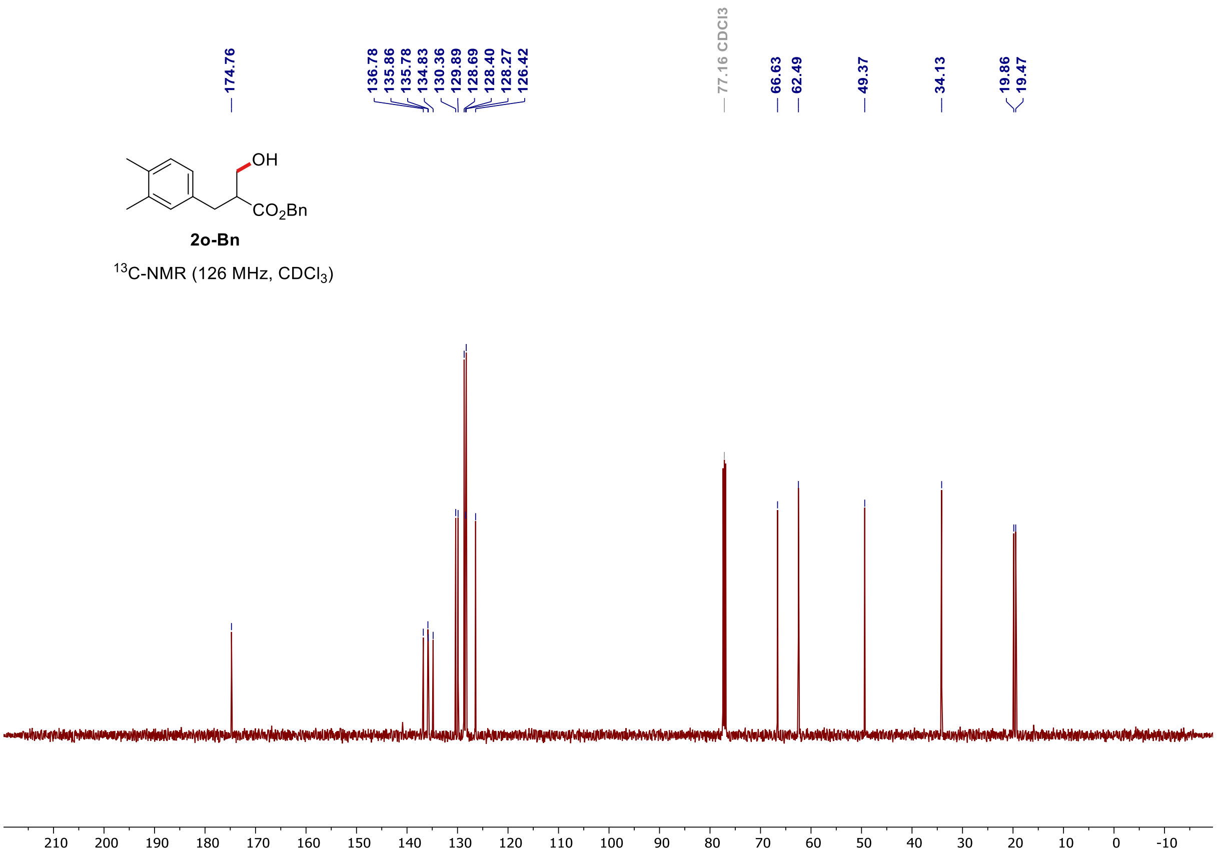

174.76
136.78 135.86 135.78 134.83 130.36 129.89 128.69 128.40 128.27 126.42
77.16 CDCl3
66.63 62.49
49.37
34.13
19.86 19.47
OH
CO_2Bn
2o-Bn
^{13}C-NMR (126 MHz, $CDCl_3$)
210 200 190 180 170 160 150 140 130 120 110 100 90 80 70 60 50 40 30 20 10 0 -10

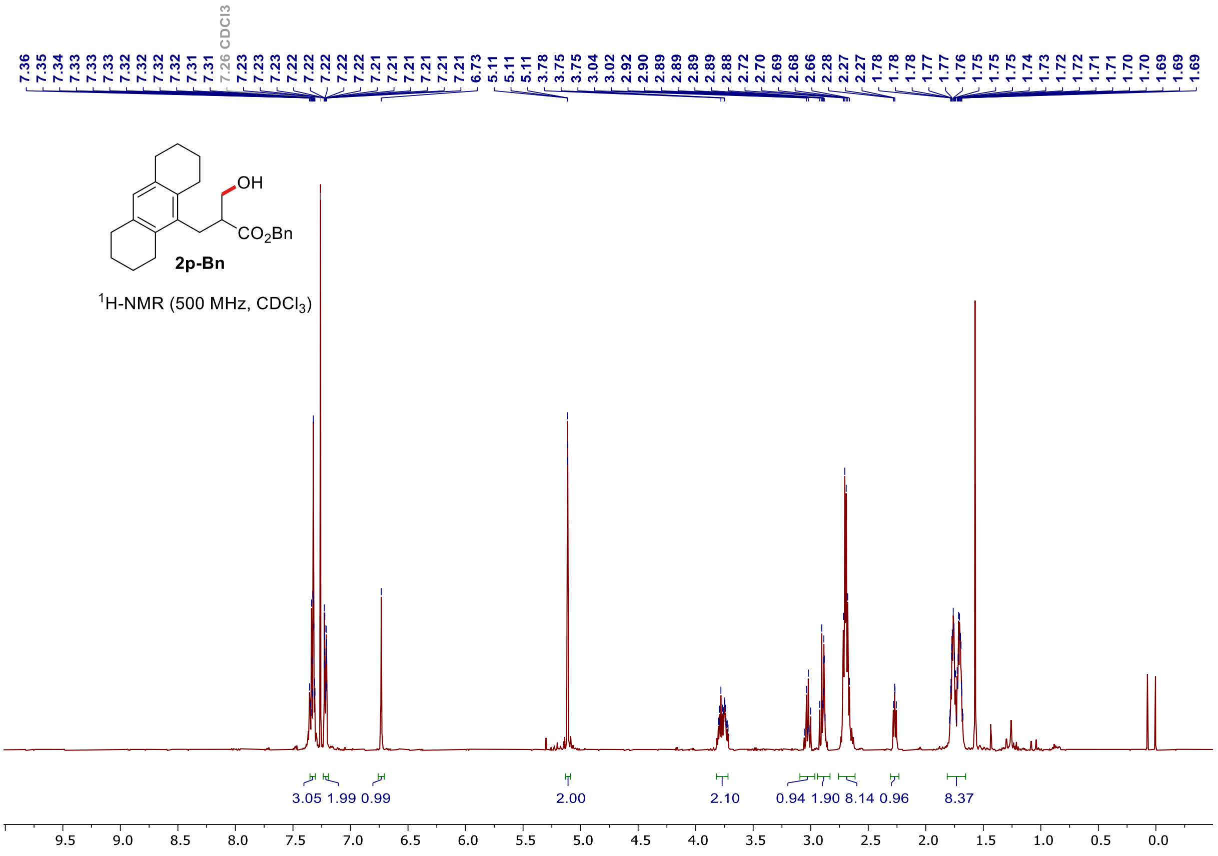

OH
CO_2Bn
2p-Bn
^{1}H-NMR (500 MHz, $CDCl_3$)
7.26 CDCl3
3.05 1.99 0.99
2.00
2.10
0.94 1.90 8.14 0.96
8.37

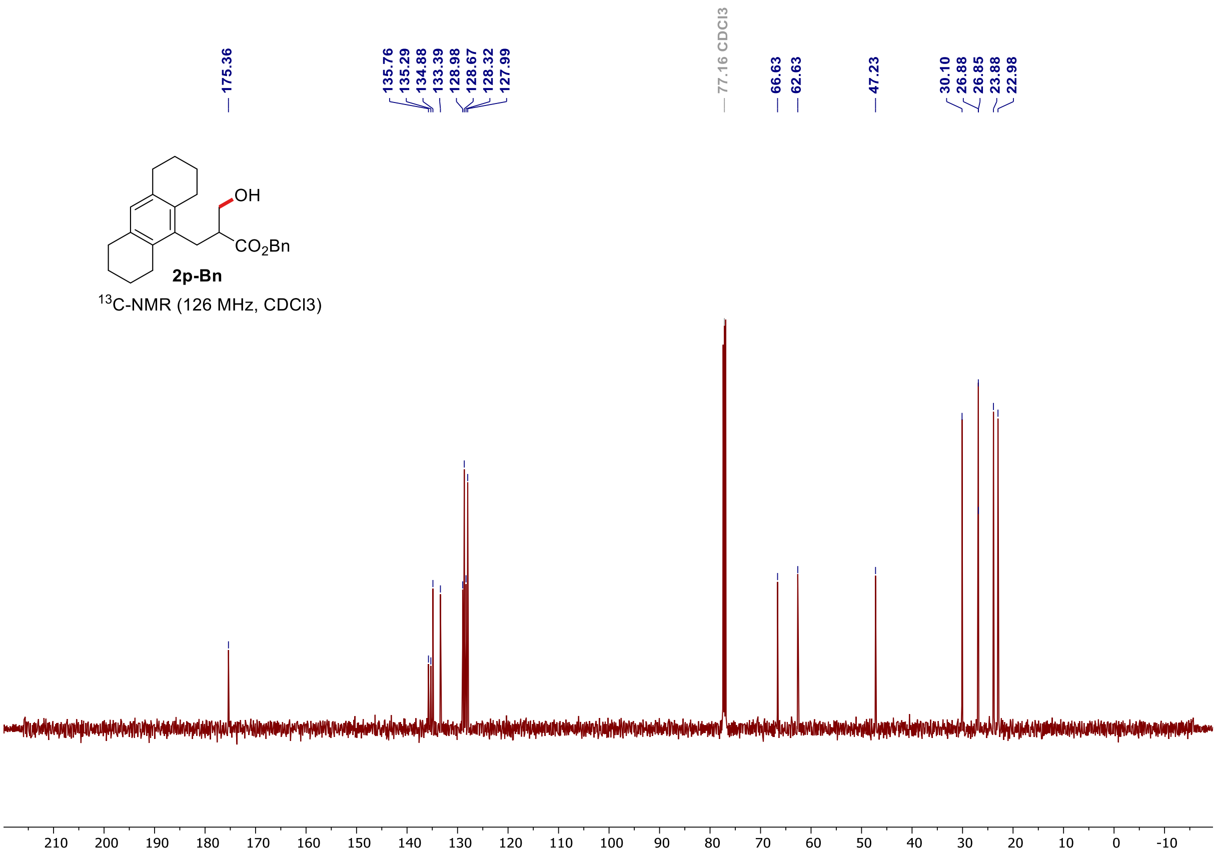

175.36
135.76 135.29 134.88 133.39 128.98 128.67 128.32 127.99
77.16 CDCl3
66.63
62.63
47.23
30.10 26.88 26.85 23.88 22.98
OH
CO_2Bn
2p-Bn
^{13}C-NMR (126 MHz, CDCl3)

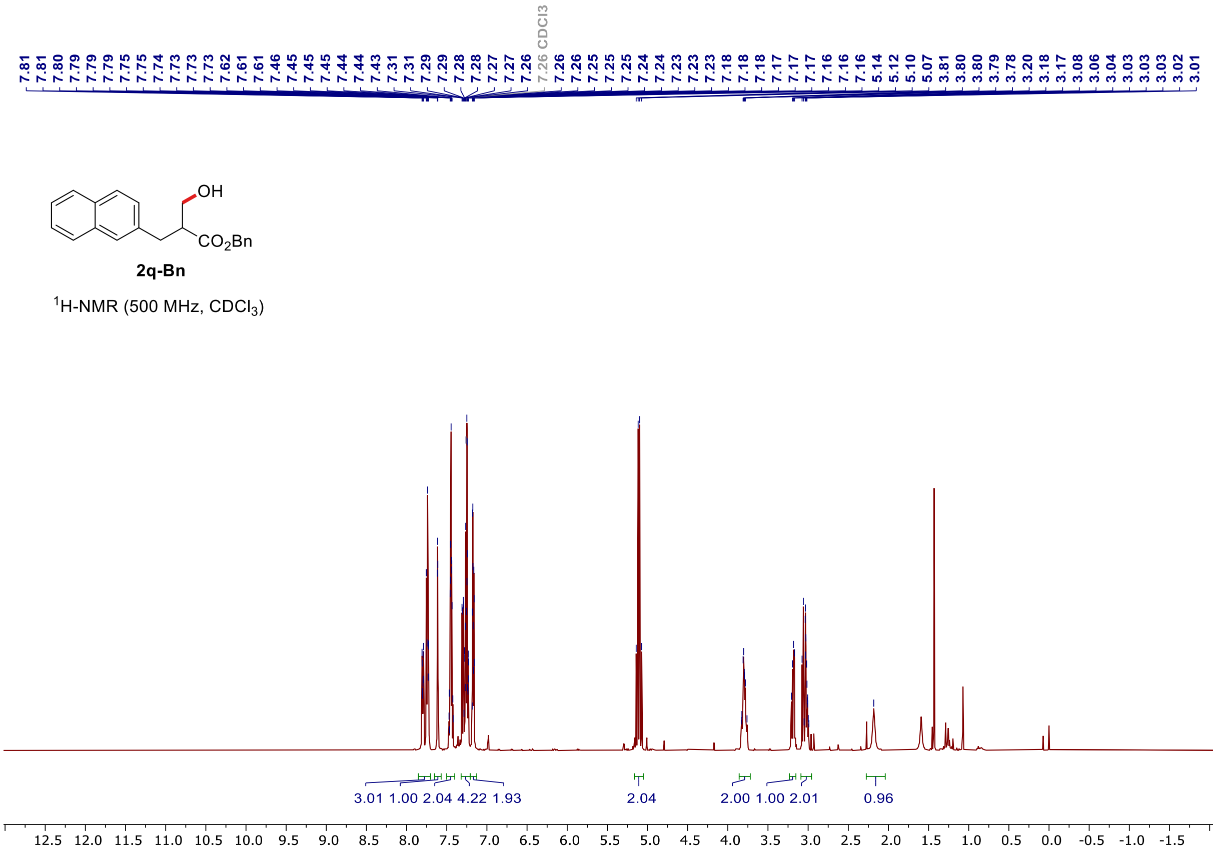
7.81 7.81 7.80 7.79 7.79 7.79 7.75 7.75 7.74 7.73 7.73 7.73 7.62 7.61 7.61 7.46 7.45 7.45 7.45 7.44 7.44 7.43 7.31 7.31 7.29 7.29 7.28 7.28 7.27 7.27 7.26
7.26 CDCl3
7.26 7.26 7.25 7.25 7.25 7.24 7.24 7.24 7.23 7.23 7.23 7.18 7.18 7.18 7.17 7.17 7.17 7.16 7.16 7.16 5.14 5.12 5.10 5.07 3.81 3.80 3.80 3.79 3.78 3.20 3.18 3.17 3.08 3.06 3.04 3.03 3.03 3.03 3.02 3.01
OH
CO_2Bn
2q-Bn
^{1}H-NMR (500 MHz, $CDCl_3$)
3.01 1.00 2.04 4.22 1.93
2.04
2.00 1.00 2.01
0.96
12.5 12.0 11.5 11.0 10.5 10.0 9.5 9.0 8.5 8.0 7.5 7.0 6.5 6.0 5.5 5.0 4.5 4.0 3.5 3.0 2.5 2.0 1.5 1.0 0.5 0.0 -0.5 -1.0 -1.5

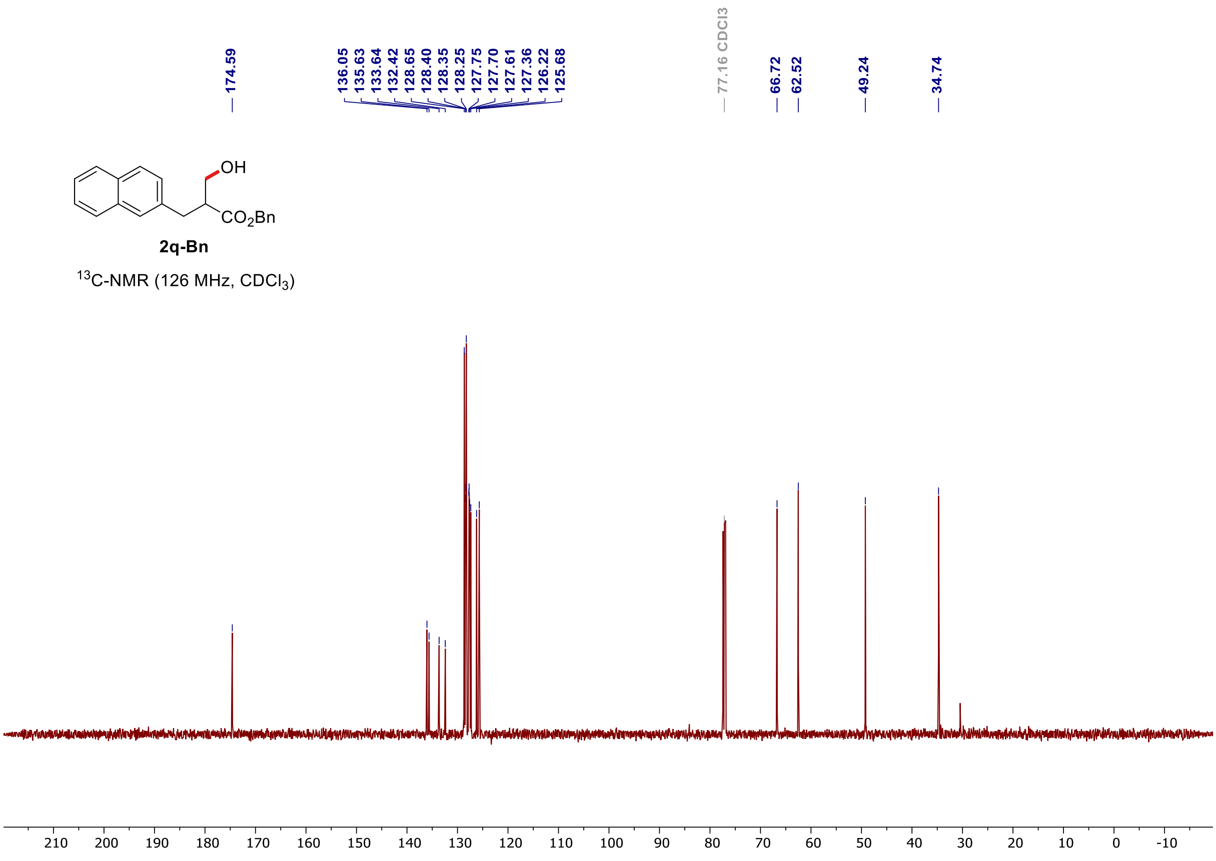
174.59
136.05 135.63 133.64 132.42 128.65 128.40 128.35 128.25 127.75 127.70 127.61 127.36 126.22 125.68
77.16 CDCl3
66.72
62.52
49.24
34.74
OH
CO_2Bn
2q-Bn
^{13}C-NMR (126 MHz, $CDCl_3$)
210 200 190 180 170 160 150 140 130 120 110 100 90 80 70 60 50 40 30 20 10 0 -10

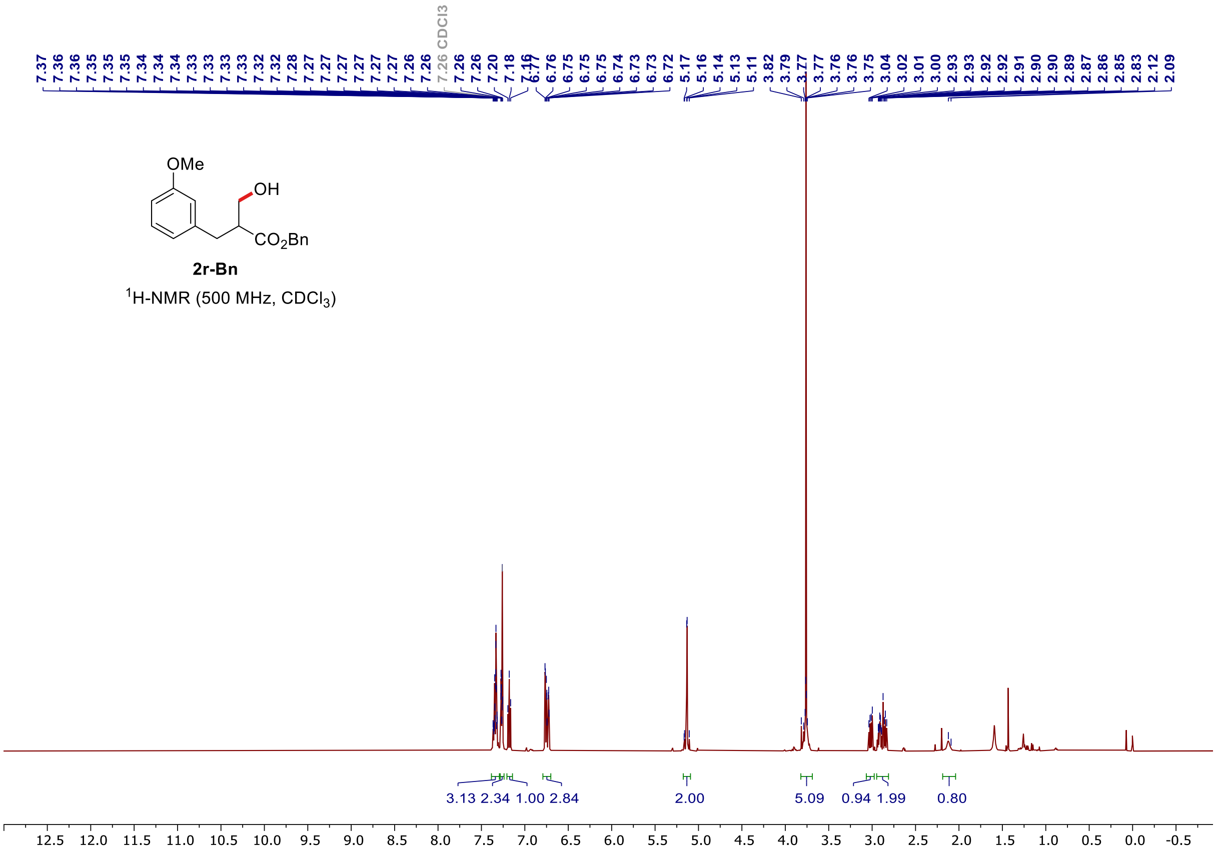
7.37 7.36 7.35 7.35 7.34 7.34 7.33 7.33 7.33 7.32 7.32 7.28 7.27 7.27 7.27 7.27 7.27 7.26 7.26 7.26 CDCl3 7.26 7.26 7.20 7.18 7.16 6.77 6.76 6.75 6.75 6.74 6.73 6.73 6.72 5.17 5.16 5.14 5.13 5.11 3.82 3.79 3.77 3.77 3.76 3.75 3.04 3.02 3.01 3.00 2.93 2.93 2.92 2.92 2.91 2.90 2.90 2.89 2.87 2.86 2.85 2.83 2.12 2.09
OMe
OH
CO2Bn
2r-Bn
1H-NMR (500 MHz, CDCl3)
3.13 2.34 1.00 2.84
2.00
5.09
0.94 1.99
0.80
12.5 12.0 11.5 11.0 10.5 10.0 9.5 9.0 8.5 8.0 7.5 7.0 6.5 6.0 5.5 5.0 4.5 4.0 3.5 3.0 2.5 2.0 1.5 1.0 0.5 0.0 -0.5

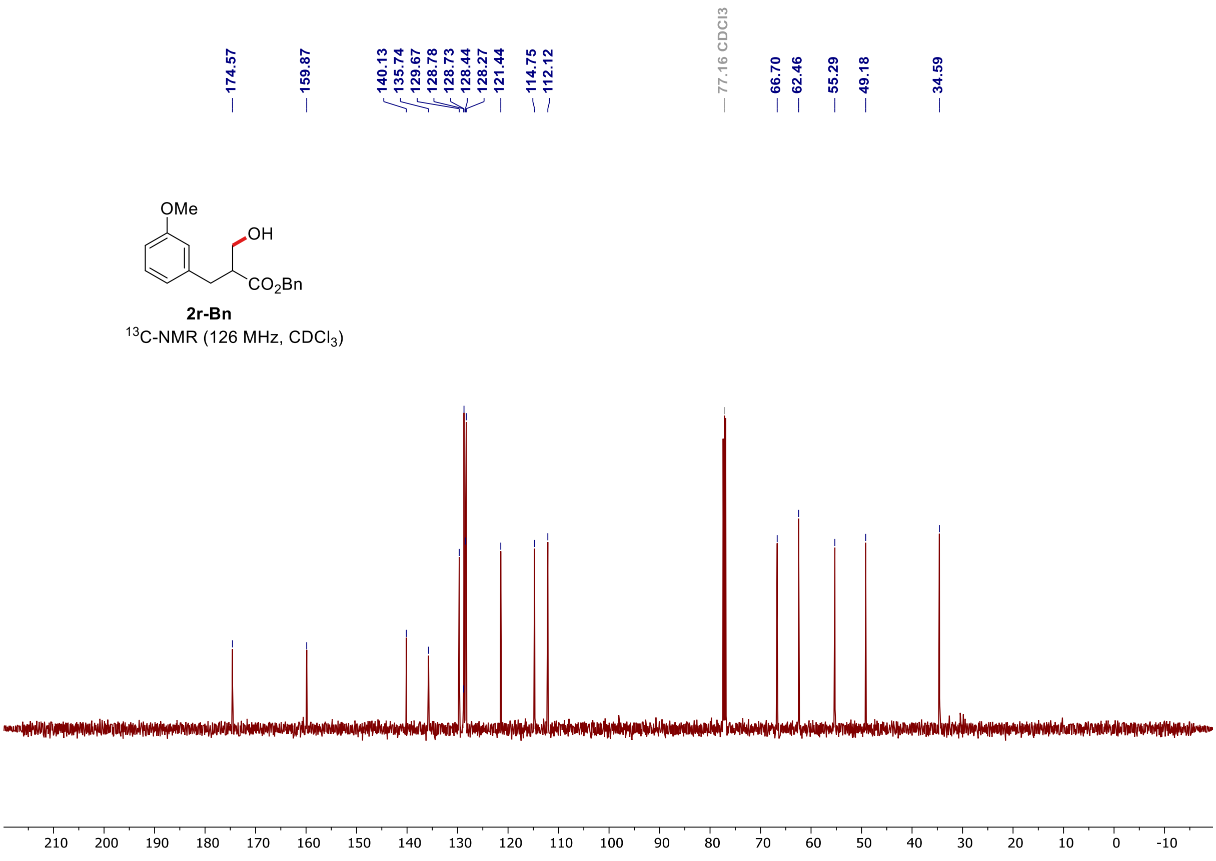
174.57
159.87
140.13 135.74 129.67 128.78 128.73 128.44 128.27 121.44
114.75 112.12
77.16 CDCl3
66.70 62.46 55.29 49.18
34.59
OMe
OH
CO2Bn
2r-Bn
13C-NMR (126 MHz, CDCl3)
210 200 190 180 170 160 150 140 130 120 110 100 90 80 70 60 50 40 30 20 10 0 -10

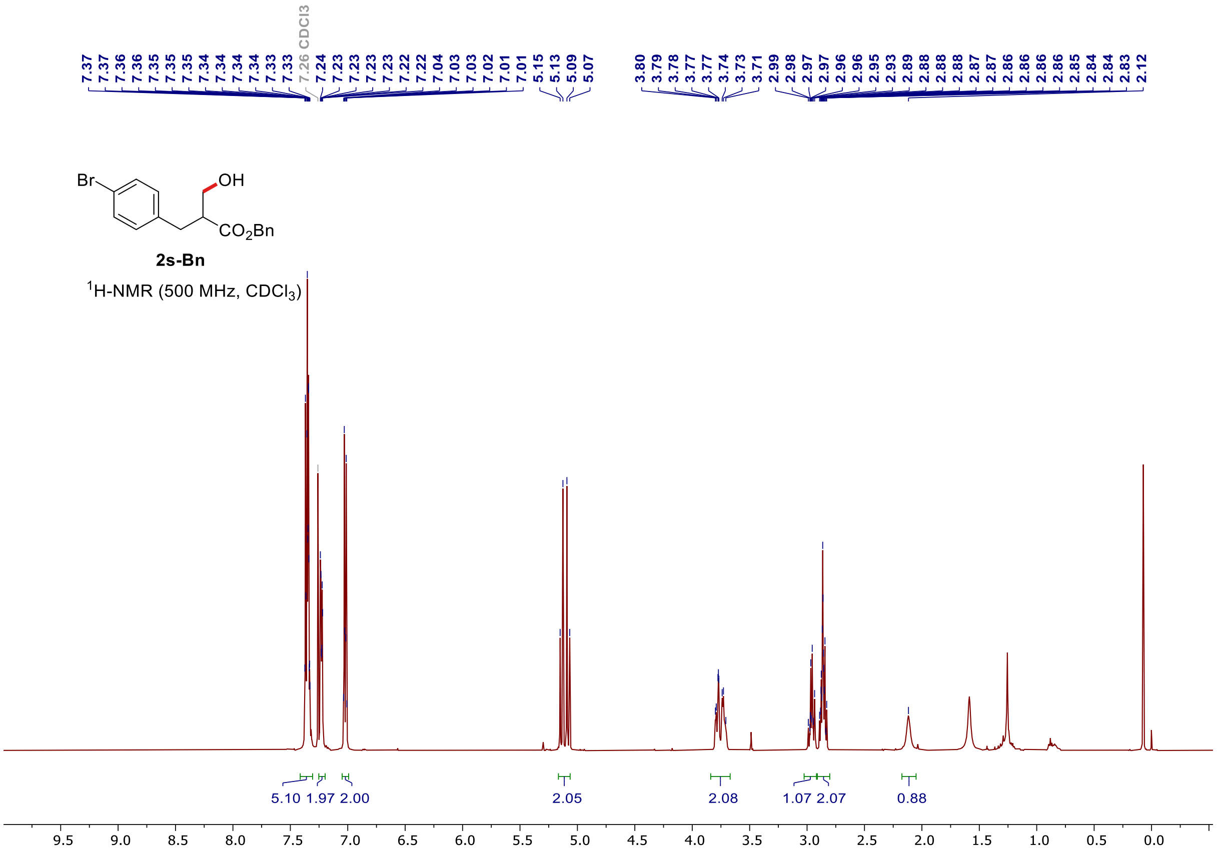
7.37 7.37 7.36 7.35 7.35 7.35 7.34 7.34 7.34 7.34 7.33 7.33 7.26 CDCl3 7.24 7.23 7.23 7.23 7.23 7.22 7.22 7.04 7.03 7.03 7.02 7.02 7.01 7.01 5.15 5.13 5.09 5.07
3.80 3.79 3.78 3.77 3.77 3.74 3.73 3.71 2.99 2.98 2.97 2.97 2.96 2.96 2.95 2.93 2.89 2.88 2.88 2.88 2.87 2.87 2.86 2.86 2.86 2.86 2.85 2.84 2.84 2.83 2.12
Br
OH
CO_2Bn
2s-Bn
^{1}H-NMR (500 MHz, $CDCl_3$)
5.10 1.97 2.00
2.05
2.08
1.07 2.07
0.88
9.5 9.0 8.5 8.0 7.5 7.0 6.5 6.0 5.5 5.0 4.5 4.0 3.5 3.0 2.5 2.0 1.5 1.0 0.5 0.0

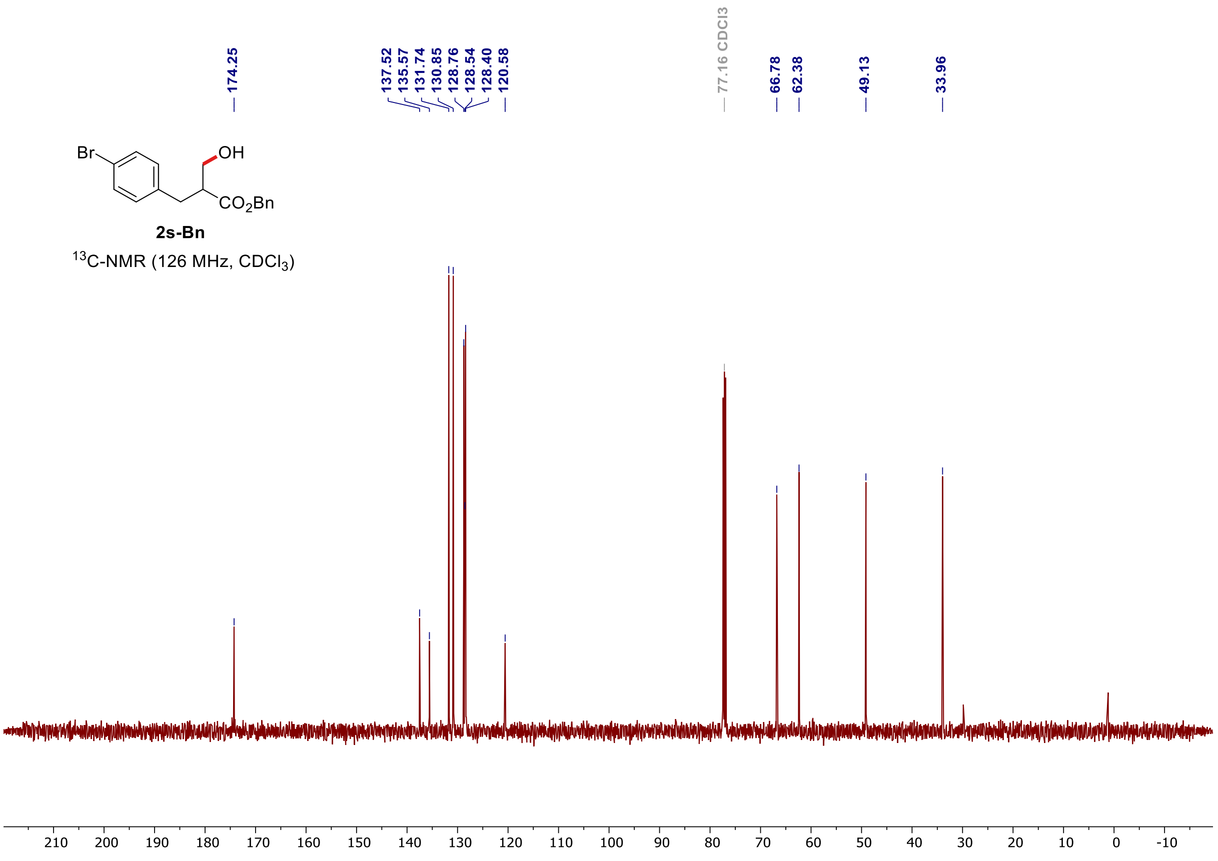
174.25
137.52 135.57 131.74 130.85 128.76 128.54 128.40 120.58
77.16 CDCl3
66.78
62.38
49.13
33.96
Br
OH
CO_2Bn
2s-Bn
^{13}C-NMR (126 MHz, $CDCl_3$)
210 200 190 180 170 160 150 140 130 120 110 100 90 80 70 60 50 40 30 20 10 0 -10

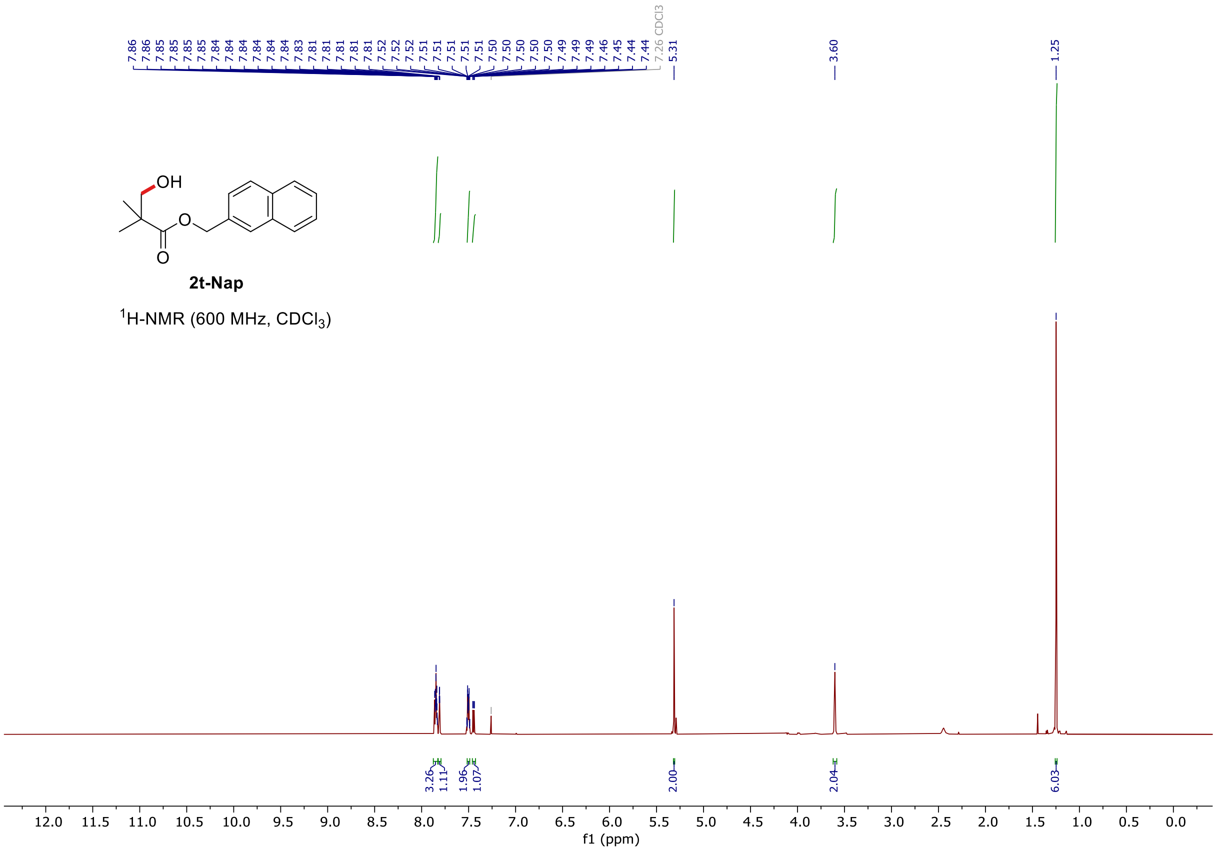

2t-Nap

^{1}H-NMR (600 MHz, $CDCl_3$)

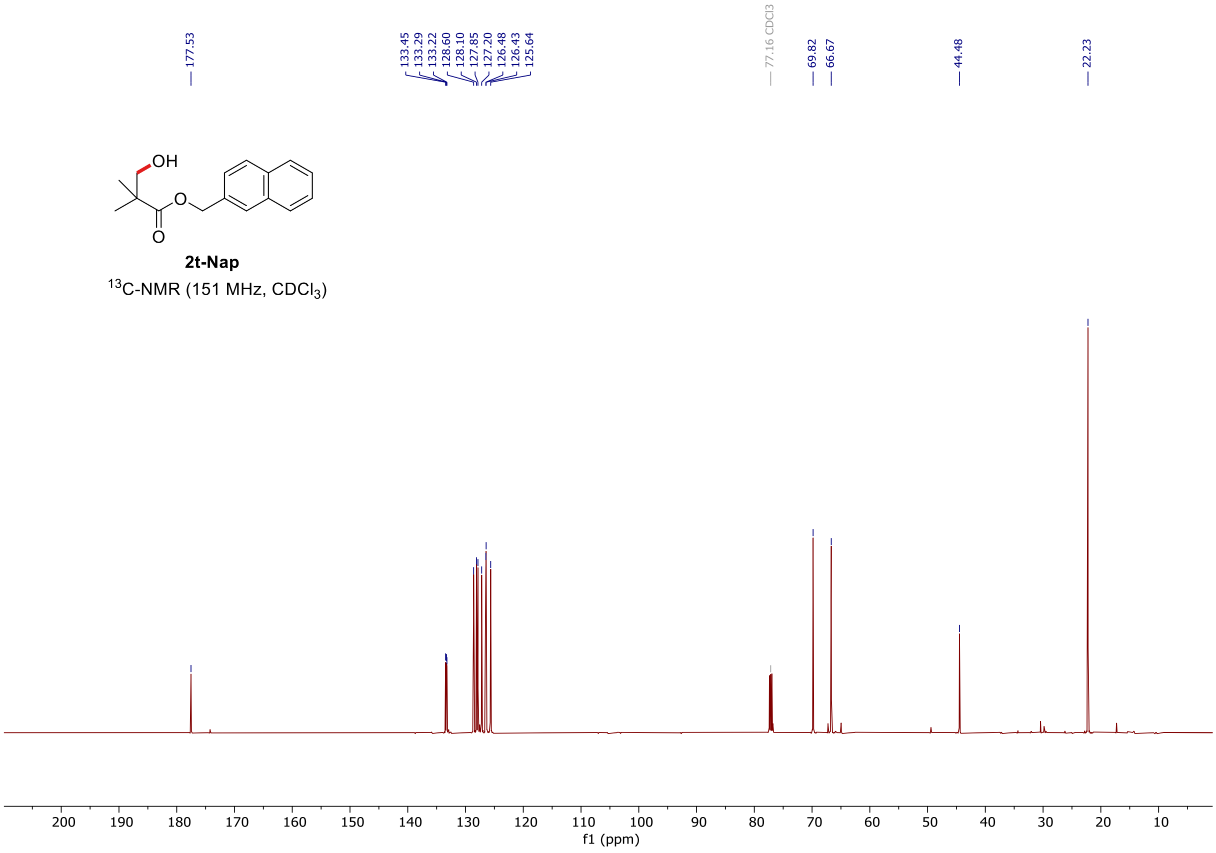

2t-Nap

^{13}C-NMR (151 MHz, $CDCl_3$)

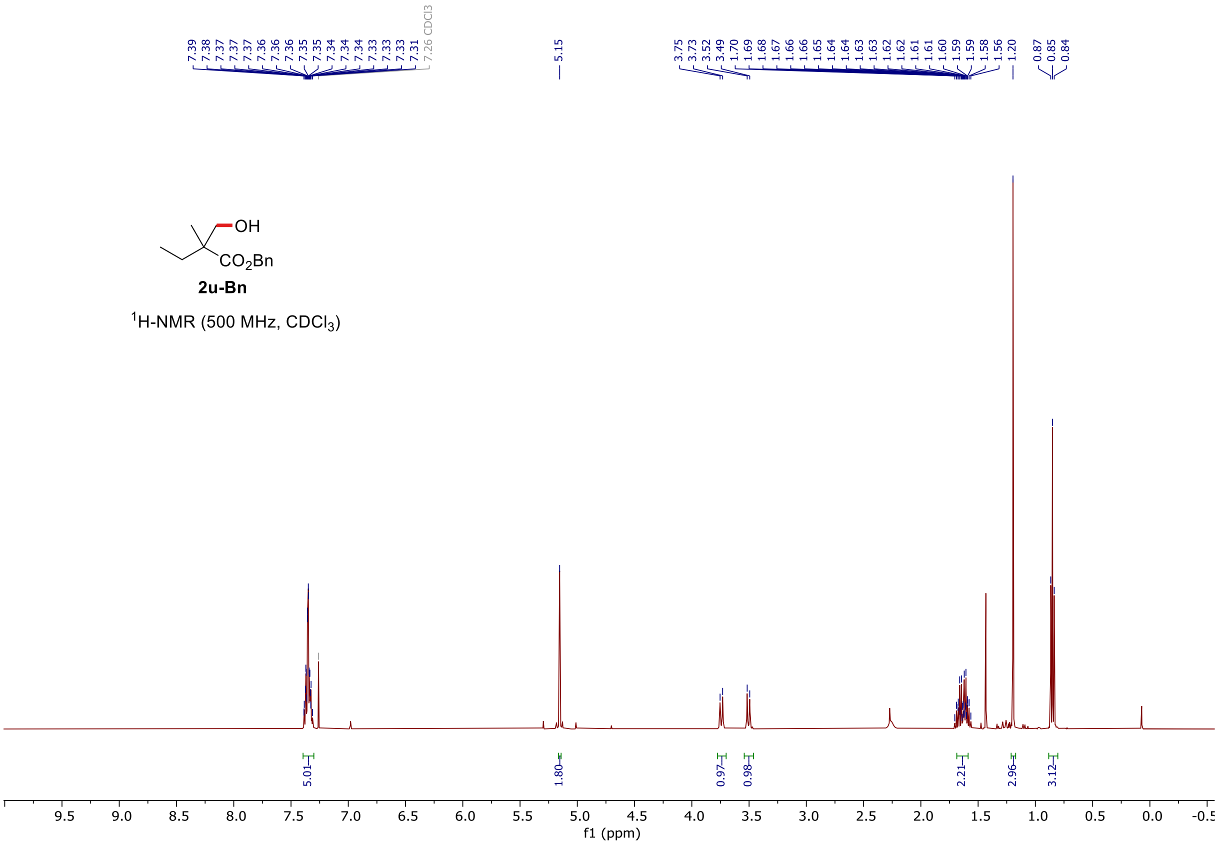
7.39 7.38 7.37 7.37 7.37 7.36 7.36 7.36 7.35 7.35 7.34 7.34 7.34 7.33 7.33 7.33 7.31 7.26 CDCl3
5.15
3.75 3.73 3.52 3.49 1.70 1.69 1.68 1.67 1.66 1.66 1.65 1.64 1.64 1.63 1.63 1.62 1.62 1.61 1.61 1.60 1.59 1.59 1.58 1.56 1.20 0.87 0.85 0.84
OH
CO2Bn
2u-Bn
1H-NMR (500 MHz, CDCl3)
5.01 1.80 0.97 0.98 2.21 2.96 3.12
f1 (ppm)

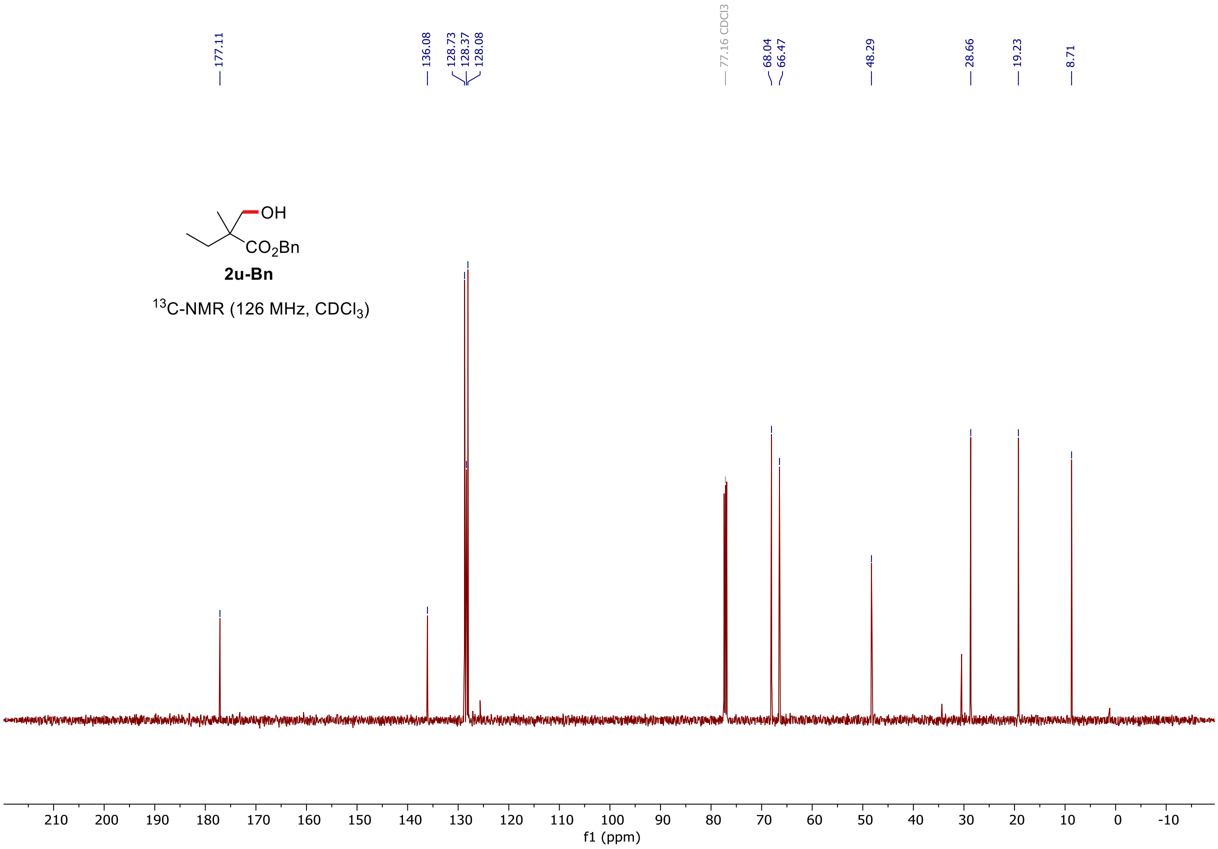
177.11
136.08 128.73 128.37 128.08
77.16 CDCl3
68.04 66.47
48.29
28.66
19.23
8.71
OH
CO2Bn
2u-Bn
13C-NMR (126 MHz, CDCl3)
f1 (ppm)

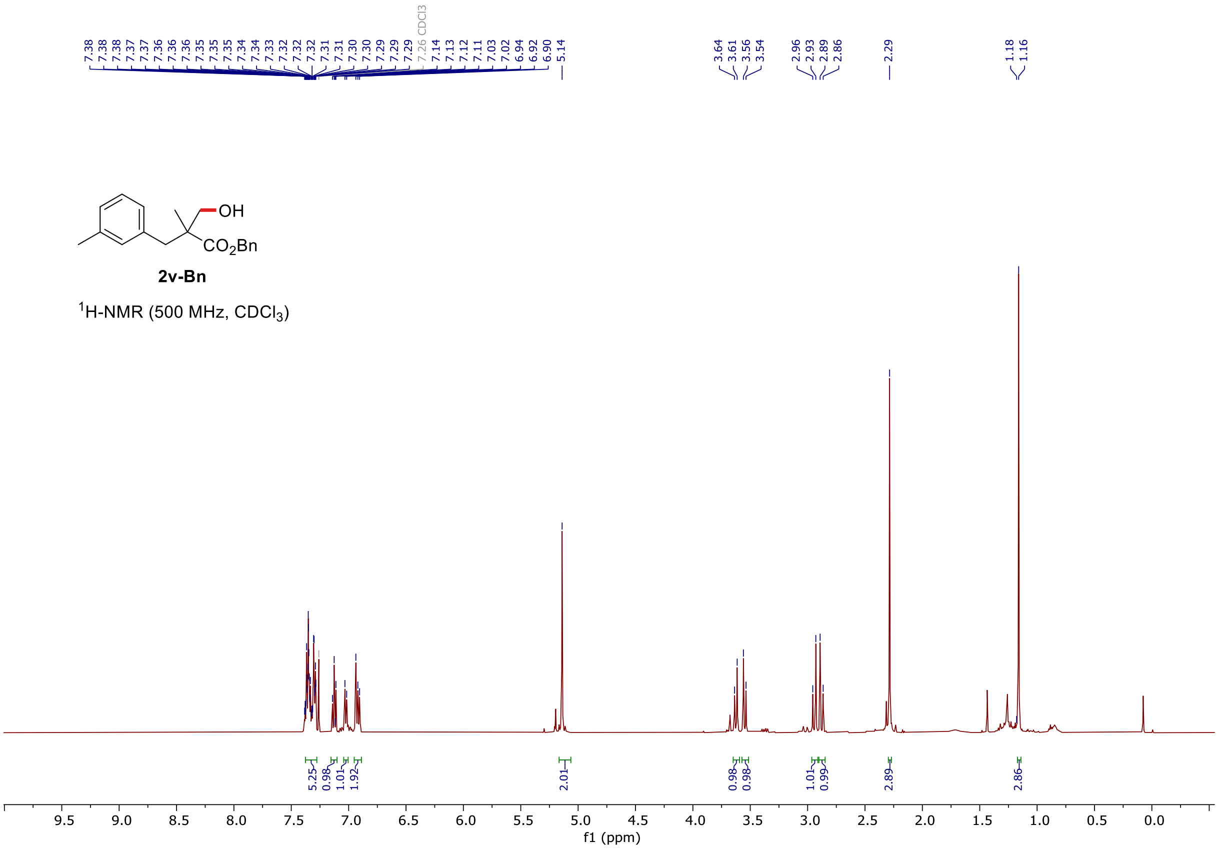
7.38 7.38 7.38 7.37 7.37 7.36 7.36 7.36 7.35 7.35 7.35 7.34 7.34 7.33 7.32 7.32 7.32 7.31 7.31 7.30 7.30 7.29 7.29 7.29 7.26 CDCl3 7.14 7.13 7.12 7.11 7.03 7.02 6.94 6.92 6.90 5.14
3.64 3.61 3.56 3.54 2.96 2.93 2.89 2.86 2.29 1.18 1.16
OH
CO2Bn
2v-Bn
1H-NMR (500 MHz, CDCl3)
5.25 0.98 1.01 1.92 2.01 0.98 0.98 1.01 0.99 2.89 2.86
f1 (ppm)

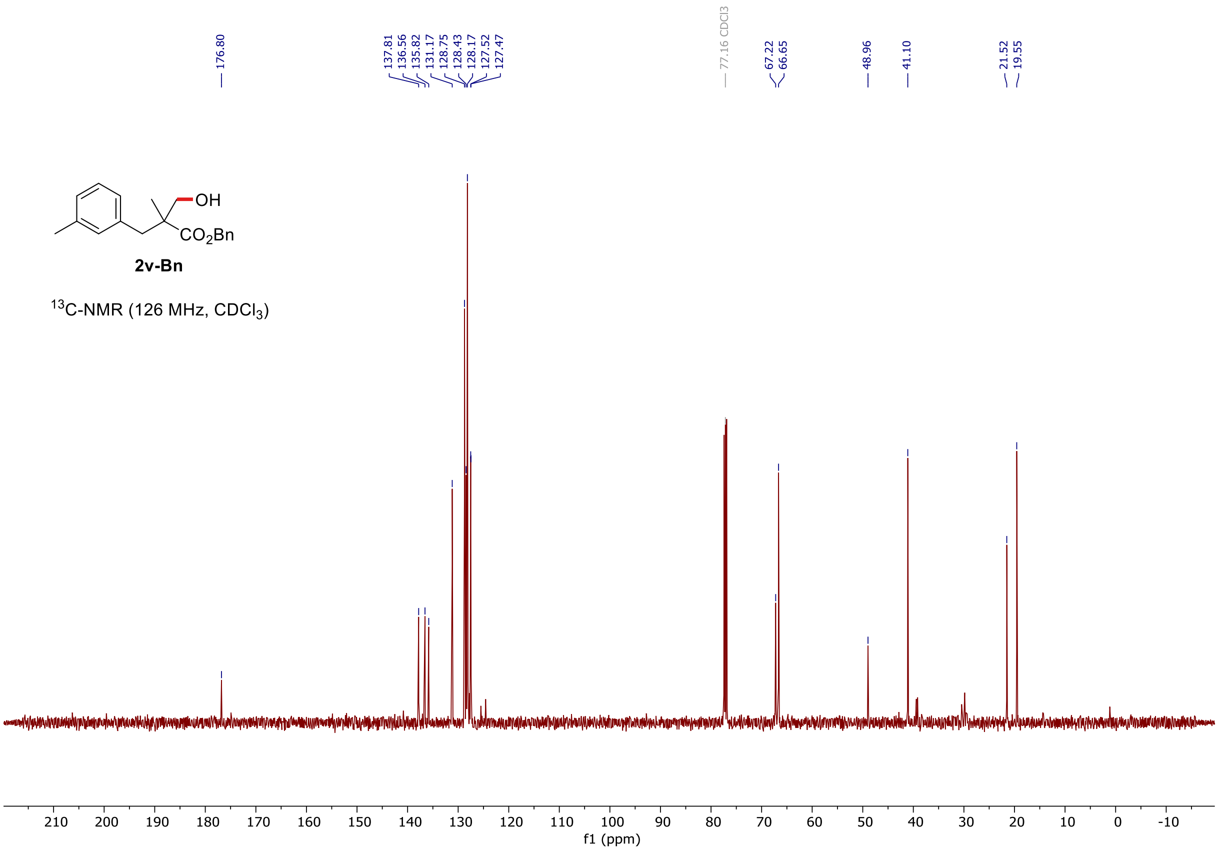
176.80
137.81 136.56 135.82 131.17 128.75 128.43 128.17 127.52 127.47
77.16 CDCl3
67.22 66.65 48.96 41.10 21.52 19.55
OH
CO2Bn
2v-Bn
13C-NMR (126 MHz, CDCl3)
f1 (ppm)

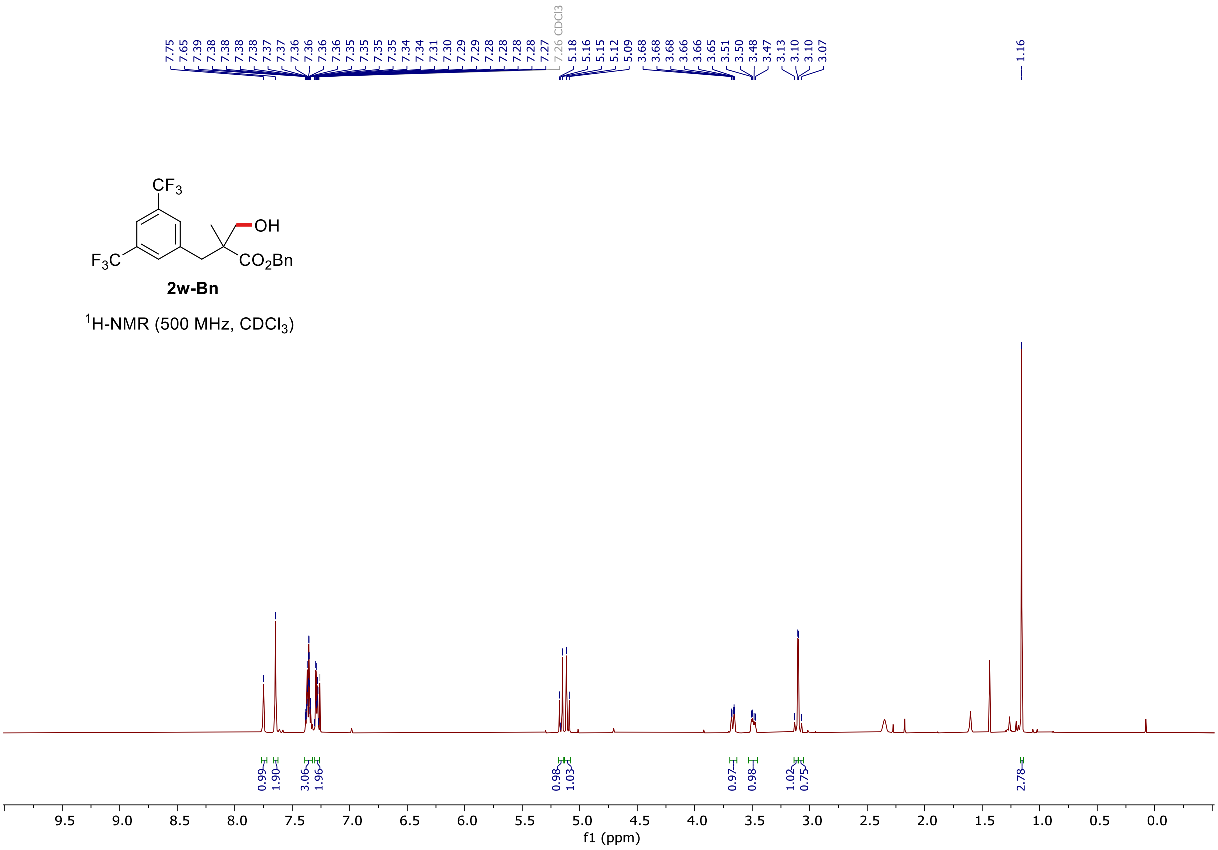
7.75 7.65 7.39 7.38 7.38 7.38 7.38 7.37 7.37 7.36 7.36 7.36 7.36 7.35 7.35 7.35 7.35 7.34 7.34 7.31 7.30 7.29 7.29 7.28 7.28 7.28 7.28 7.27
7.26 CDCl3
5.18 5.16 5.15 5.12 5.09 3.68 3.68 3.68 3.66 3.66 3.65 3.51 3.50 3.48 3.47 3.13 3.10 3.10 3.07
1.16
CF3
OH
F3C
CO2Bn
2w-Bn
1H-NMR (500 MHz, CDCl3)
0.99 1.90 3.06 1.96 0.98 1.03 0.97 0.98 1.02 0.75 2.78
9.5 9.0 8.5 8.0 7.5 7.0 6.5 6.0 5.5 5.0 4.5 4.0 3.5 3.0 2.5 2.0 1.5 1.0 0.5 0.0
f1 (ppm)

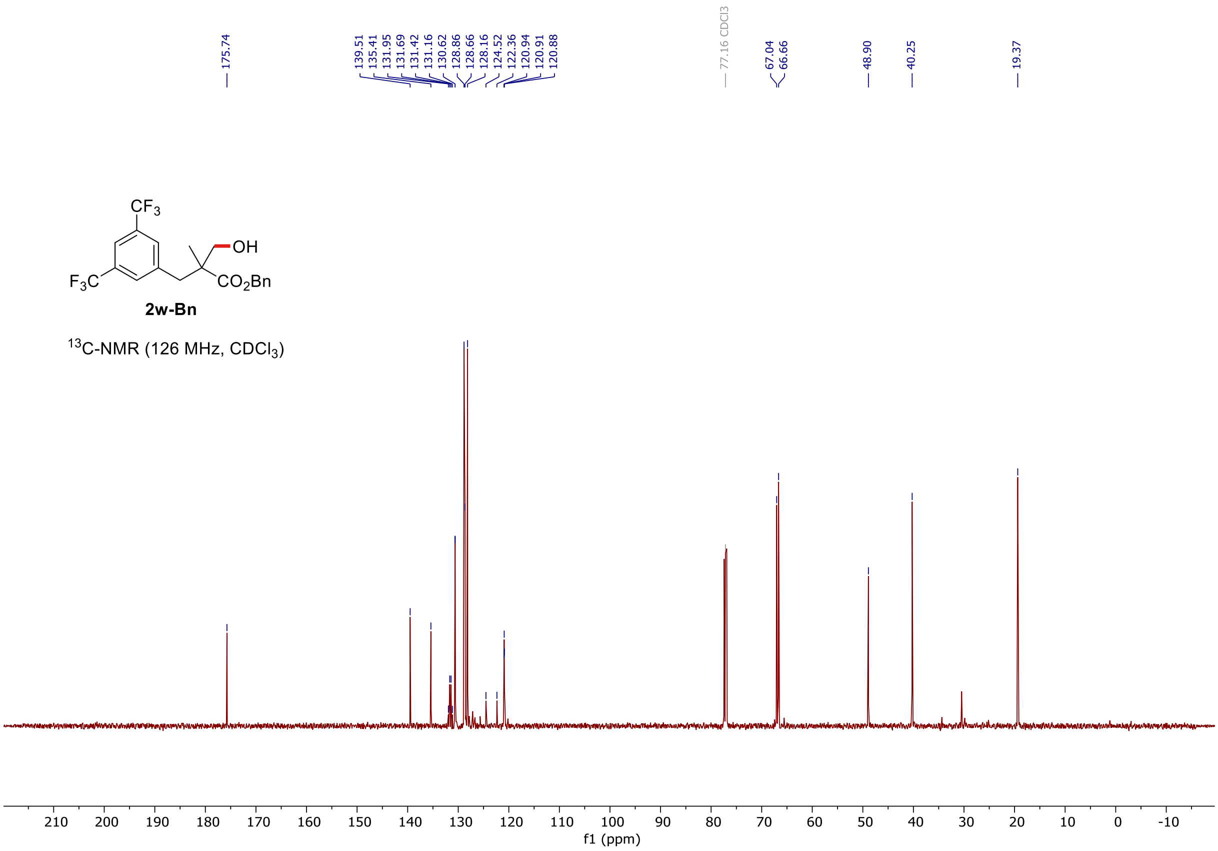
175.74
139.51 135.41 131.95 131.69 131.42 131.16 130.62 128.86 128.66 128.16 124.52 122.36 120.94 120.91 120.88
77.16 CDCl3
67.04 66.66
48.90
40.25
19.37
CF3
OH
F3C
CO2Bn
2w-Bn
13C-NMR (126 MHz, CDCl3)
210 200 190 180 170 160 150 140 130 120 110 100 90 80 70 60 50 40 30 20 10 0 -10
f1 (ppm)

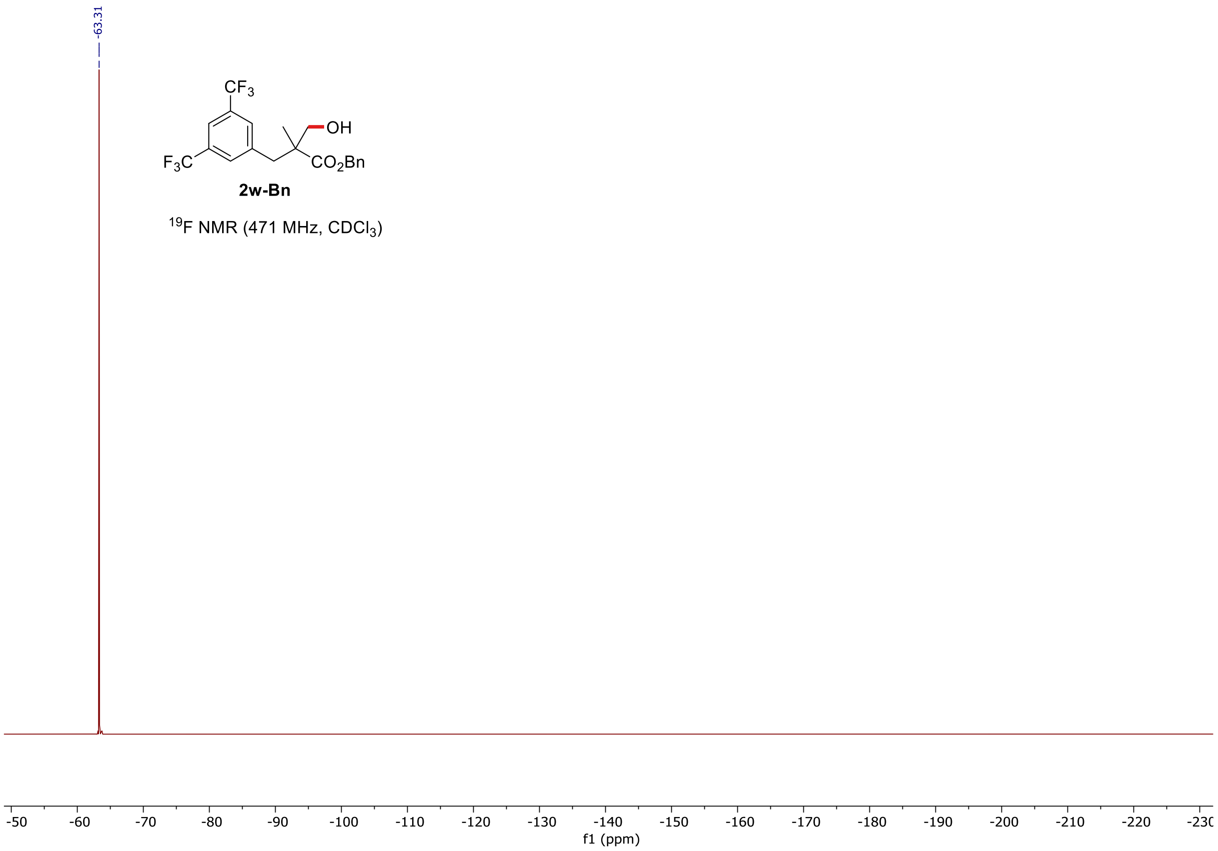

-63.31
CF3
OH
F3C
CO2Bn
2w-Bn
19F NMR (471 MHz, CDCl3)
-50
-60
-70
-80
-90
-100
-110
-120
-130
-140
-150
-160
-170
-180
-190
-200
-210
-220
-230
f1 (ppm)

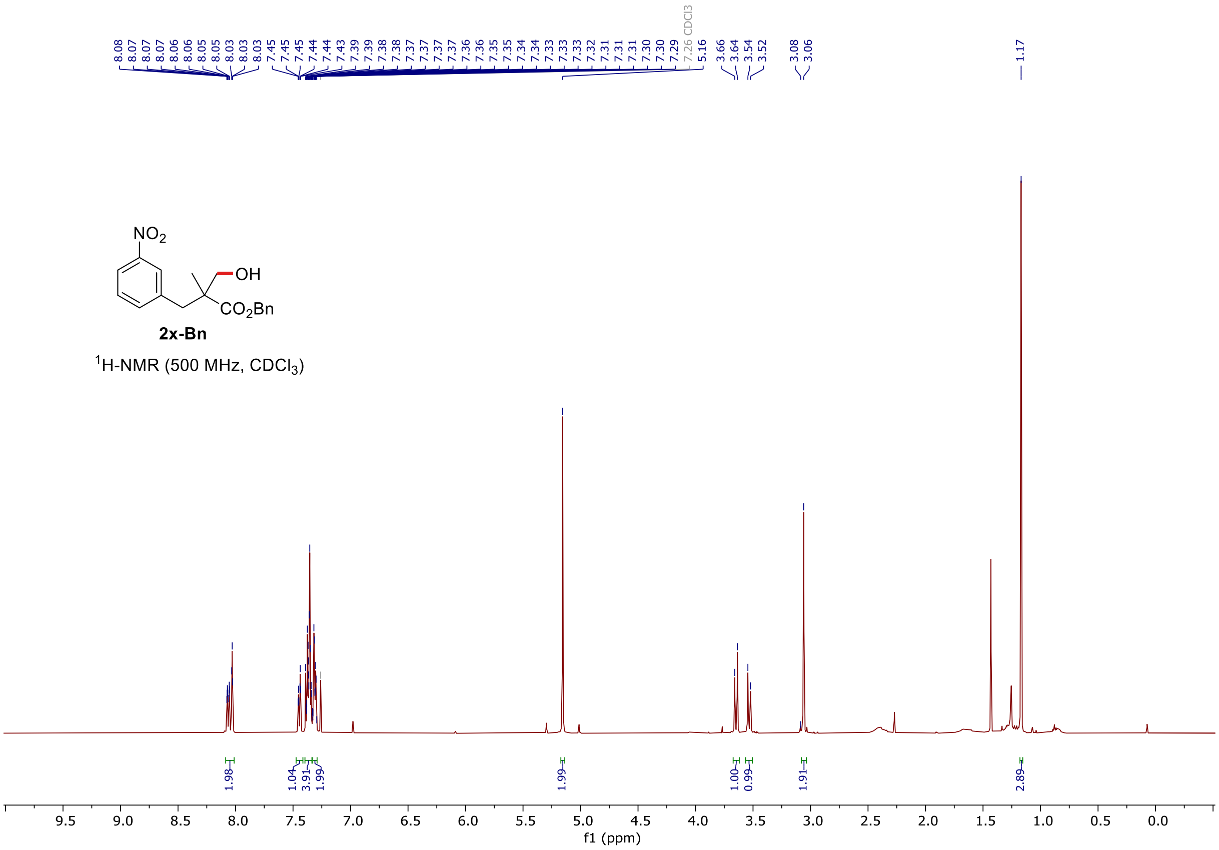

NO2
OH
CO2Bn
2x-Bn
1H-NMR (500 MHz, CDCl3)
f1 (ppm)

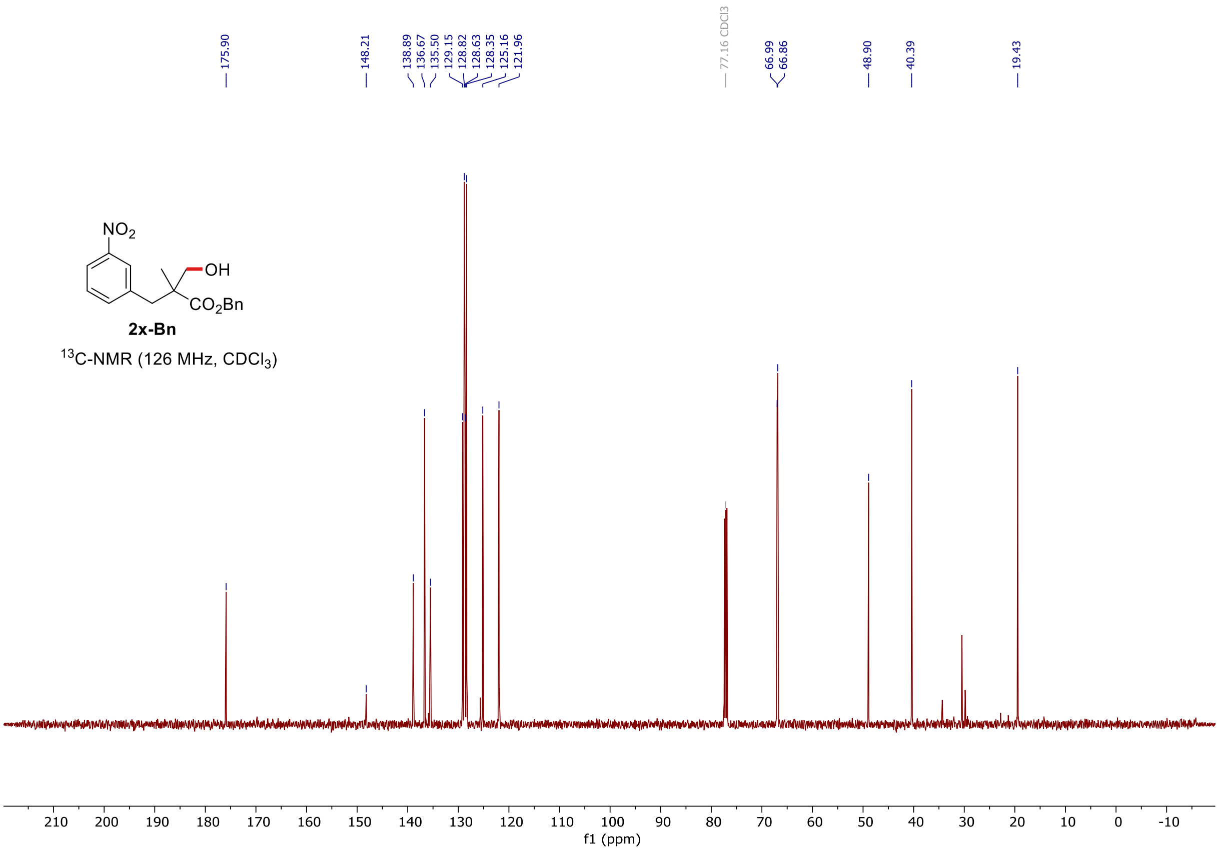

NO2
OH
CO2Bn
2x-Bn
13C-NMR (126 MHz, CDCl3)
f1 (ppm)

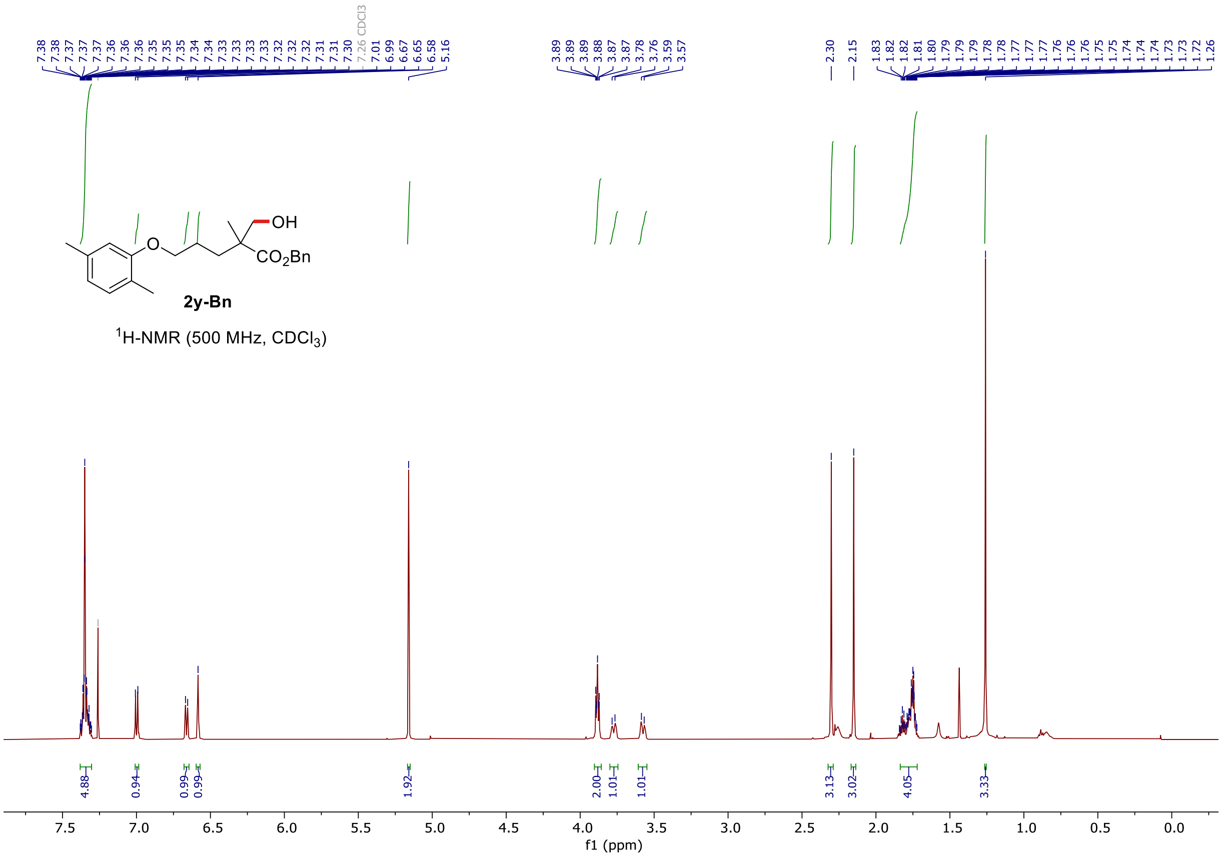
OH
CO_2Bn
2y-Bn
^{1}H-NMR (500 MHz, $CDCl_3$)
f1 (ppm)

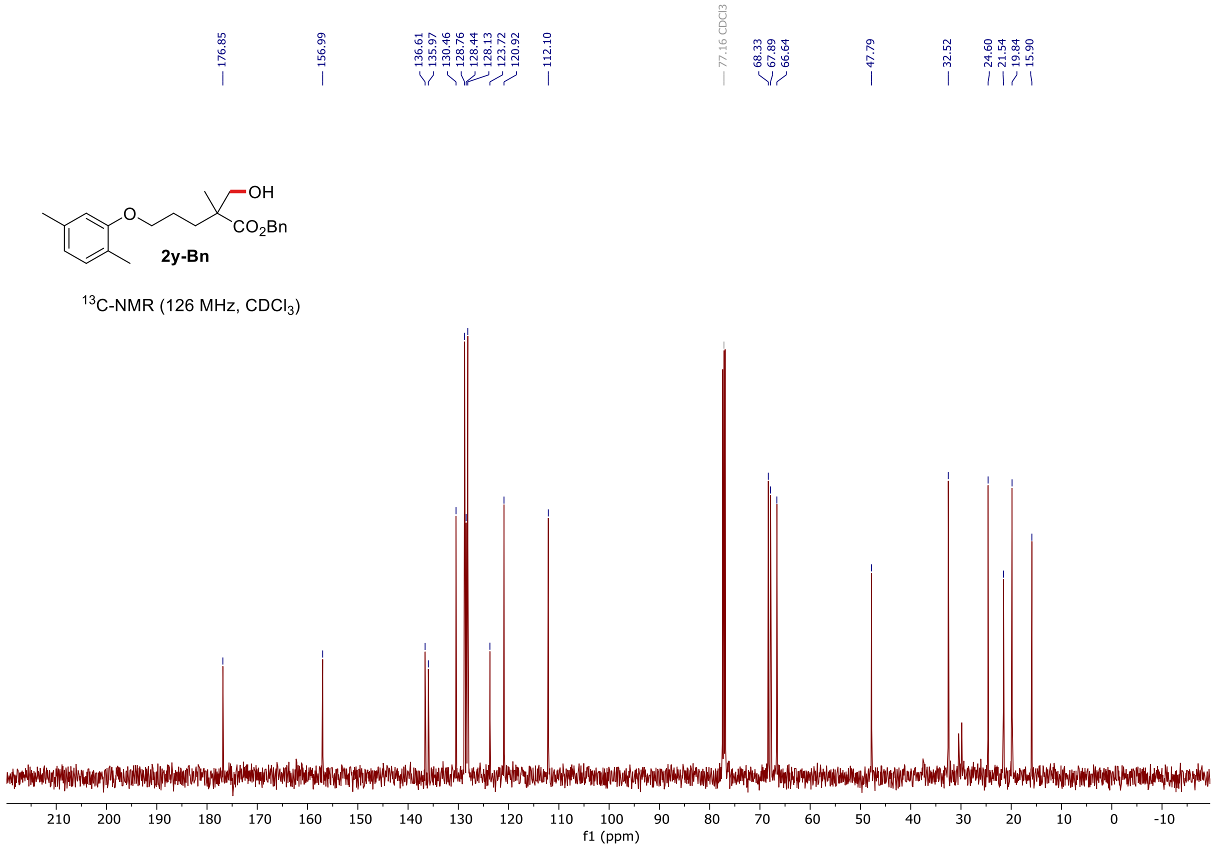
OH
CO_2Bn
2y-Bn
^{13}C-NMR (126 MHz, $CDCl_3$)
f1 (ppm)
